# Coral-Derived Endophytic Fungal Product, Butyrolactone-I, Alleviates Lps Induced Intestinal Epithelial Cell Inflammatory Response Through TLR4/NF-κB and MAPK Signaling Pathways: An *in vitro* and *in vivo* Studies

**DOI:** 10.3389/fnut.2021.748118

**Published:** 2021-10-01

**Authors:** Shengwei Chen, Yi Zhang, Xueting Niu, Sahar Ghulam Mohyuddin, Jiayin Wen, Minglong Bao, Tianyue Yu, Lianyun Wu, Canyin Hu, Yanhong Yong, Xiaoxi Liu, A. M. Abd El-Aty, Xianghong Ju

**Affiliations:** ^1^Department of Veterinary Medicine, Guangdong Ocean University, Zhanjiang, China; ^2^Shenzhen Institute of Guangdong Ocean University, Shenzhen, China; ^3^College of Food Science and Technology, Guangdong Ocean University, Zhanjiang, China; ^4^State Key Laboratory of Bio Based Material and Green Papermaking, College of Food Science and Engineering, Qilu University of Technology, Shandong Academy of Science, Jinan, China; ^5^Department of Pharmacology, Faculty of Veterinary Medicine, Cairo University, Giza, Egypt; ^6^Department of Medical Pharmacology, Faculty of Medicine, Atatürk University, Erzurum, Turkey

**Keywords:** butyrolactone-I, anti-inflammatory, intestinal barrier, IBD, TLR4/NF-κB, MAPK

## Abstract

Herein, we assessed the anti-inflammatory and intestinal barrier protective effects of butyrolactone-I (BTL-1), derived from the coral-derived endophytic fungus (*Aspergillus terreus*), using the LPS-induced IPEC-J2 inflammation model and the DSS-induced IBD model in mice. In IPEC-J2 cells, pretreatment with BTL-I significantly inhibited TLR4/NF-κB signaling pathway and JNK phosphorylation, resulting in the decrease of IL-1β and IL-6 expression. Interestingly, BTL-1 pretreatment activated the phosphorylation of ERK and P38, which significantly enhanced the expression of TNF-α. Meanwhile, BTL-1 pretreatment upregulated tight junction protein expression (ZO-1, occludin, and claudin-1) and maintained intestinal barrier and intestinal permeability integrity. In mice, BTL-1 significantly alleviated the intestinal inflammatory response induced by DSS, inhibited TLR4/NF-κB signaling pathway, and MAPK signaling pathway, thus reducing the production of IL-1, IL-6, and TNF-α. Further, the expression of tight junction proteins (ZO-1, occludin, and claudin-1) was upregulated in BTL-1 administrated mice. Therefore, it has been suggested that butyrolactone-I alleviates inflammatory responses in LPS-stimulated IPEC-J2 and DSS-induced murine colitis by TLR4/NF-κB and MAPK signal pathway. Thereby, BTL-1 might potentially be used as an ocean drug to prevent intestinal bowel disease.

**Graphical Abstract:**
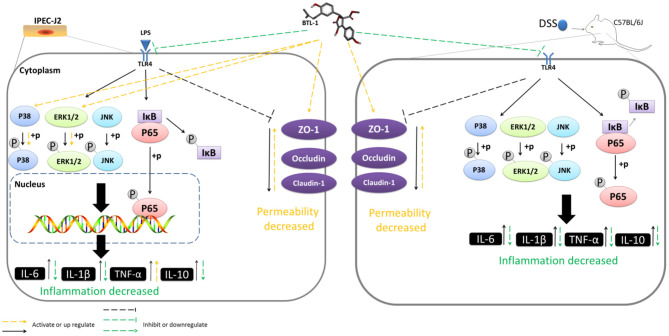
Butyrolactone-I alleviates inflammatory responses in LPS-stimulated IPEC-J2 and DSS-induced murine colitis by TLR4/NF-κB and MAPK signal pathway.

## Introduction

Inflammatory bowel diseases (IBD), including Crohn's disease (CD) and ulcerative colitis (UC), are a collection of chronically non-specific inflammatory disorders featured with chronic inflammation and abnormalities in the gastrointestinal epithelial barrier (1, 2). Although the cause of IBD has not yet been fully elucidated, current research assumed that it is mainly caused by the interaction of multiple factors, such as genetics, immunity, and gut microbes (3, 4). Presently, IBD has a high prevalence rate worldwide, and the incidence rate continues to increase. Its recurrence, persistence, and cancer risk significantly impact patients' lives. At present, there is no specific treatment for IBD. The ultimate goal of IBD treatment is to achieve clinical remission with sustained remission. IBD can be treated with glucocorticoids and immunosuppressant's; however, these drugs have substantial adverse effects (such as nephrotoxicity, hemolytic anemia, osteoporosis, insomnia, and pancreatitis) and poor clinical outcomes (5). Therefore, developing a new medicine with fewer side effects is warranted for treating IBD.

Previous studies have shown that TLR4 is the primary membrane signal receptor causing IBD inflammation. Several standard signaling' pathways, including the nuclear factor-κ B (NF-κB) and mitogen-activated protein kinases (MAPKs) pathways, play a potential role in this process (6). NF-κB is a ubiquitous transcriptional factor promoting the production of pro-inflammatory cytokines and chemokines and is essential in inflammation and immune response. IBD and other autoimmune diseases have also been linked to the NF-κB protein (7). MAPKs are composed of various serine and threonine kinases that induce inflammation (8). Other evidence suggests that inhibiting the NF-κB and MAPK signaling pathways is vital in IBD treatment (9).

Impaired epithelial barrier function is a typical symptom of IBD. Inflammation can damage the intestinal barrier integrity, resulting in the translocation of pathogens and infiltration of apical tight junction proteins (10). As a critical player in the epithelial barrier function of inflammatory bowel disease, tight junction proteins are essential for maintaining the epithelial barrier function and controlling the paracellular permeability (11). Thus, far, the relationship between the imbalance of tight junction (TJ) proteins and the occurrence of intestinal inflammation is still unclear. However, studies have pointed out that the imbalance of tight junction protein expression may precede intestinal inflammation (12), suggesting that the treatment of IBD by restoring the integrity of the epithelial barrier could be the future trend in treatment (13).

Butyrolactone-I (BTL-1) is derived from *Aspergillus terreus* C23-3, an endophytic fungus isolated from Puko Shore coral (*Porites pukoensis*) in Xuwen (Guangdong, China) (14). Previous studies have found that butyrolactone-I, as a cyclin CDC-2 kinase and CDK-2 kinase inhibitor (15), can prevent oocytes from decelerating division, inhibit tumor cell proliferation, induce cancer cell apoptosis, and prevent the apoptosis of central and peripheral neurons (16–21). Presently, researchers have discovered that it has antibacterial and anti-inflammatory activities and inhibits the secretion of NO and TNF-α in macrophages and microglia (22). However, whether BTL-1 can protect the intestinal barrier integrity and be used as a potential therapeutic drug for IBD is not fully elucidated. Herein, we established an LPS induced-inflammation model in IPEC cell and DSS-induced IBD model in mice and investigated the inhibitory effect of BTL-1 on intestinal inflammation and its protective effect on intestinal barrier integrity.

## Materials and Methods

### Chemicals and Reagents

Lipopolysaccharide (LPS) and Triton-X-100 were procured from Sigma-Aldrich (St. Louis, MO, USA). Cell Counting Kit-8 (CCK8) was secured from APExBIO Technology (Houston, USA). The commercial-specific complete Dulbecco's Modified Eagle Medium/Nutrient Mixture F-12 (DMEM/F12), penicillin, and streptomycin were acquired from GIBCO (Carlsbad, CA, USA). The ECL kit was provided by Tanon (Shanghai, China). In addition, 40, 6-diamidino-2-phenylindole (DAPI) was purchased from Beyotime (Shanghai, China). Primary and secondary antibodies used for western blotting were brought from Cell Signaling Technology (Danvers, MA, USA). Biotin-conjugated anti-Rabbit IgG antibody and DyLight 594-conjugated Avidin were picked up from Jackson ImmunoResearch (Pennsylvania, USA).

### Preparation of Butyrolactone-I

Butyrolactone-I (BTL-1) was prepared according to the method described elsewhere (23). The producer strain C23-3 was fermented at 28°C for 1 week in a 40 × 1 L flask on a rotary shaker at 150 rpm. The culture was filtered to isolate the broth and mycelium and then extracted with ethyl acetate and a combination of ethyl acetate and chloroform-methanol (1:1, *v/v*) after fermentation. The two extracts were mixed and concentrated in vacuo to make a crude extract. The extract was separated into nine fractions using a Sephadex LH-20 column treated with chloroform-methanol (1:1, *v/v*) (Fr 1 to Fr 9). Fr 7 was further purified using a Sephadex LH-20 column eluted with methanol and a preparative RP-18 HPLC eluted with a 7:3 methanol-water mixture, yielding pure compound BTL-1 (14).

### Induction of Experimental Colitis

**Figure 1:**
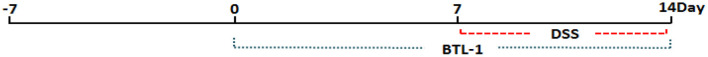
Mice treatment scheme.

Male C57BL/6J mice weighing 6–8 weeks (19–21 g) were procured from the Guangzhou Dean Gene co., ltd (Guangzhou, China) and reared in a specific pathogen-free environment with controlled temperature (26 ± 1°C) and 12-h light/dark cycle in the animal hospital of Guangdong Ocean University. The animals were acclimatized for 1 week before experimental work and given *ad libitum* access to food and water. They were fed *ad libitum* DSS (MW 36,000–50,000, Dalian Meilun Biotechnology Co., Ltd, Dalian, China) in drinking water (3.5%, *v/w*) for 7 days to develop colitis (24). The animals were randomly allocated to one of the following five groups (*n* = 5): normal control (only given food and water without DSS), DSS model (administered DSS in drinking water), low-concentration BTL-1 (administered 1 mg/kg BTL-1, p.o), middle-concentration BTL-1 (taken 2 mg/kg BTL-1, p.o), and high-concentration BTL-1 group (given 5 mg/kg BTL-1, p.o) group. A schematic overview of the experiment design is shown in Figure 1. The body weight, feed intake, and disease activity index (DAI) were recorded 3 days before feeding BTL-1. The DAI, includes changes in the consistency of stool (0, none; 1 and 2, loose stool; 3 and 4, diarrhea), bleeding (0, none; 1, trace of fecal occult blood; 2, mild occult blood; 3, obvious occult blood; 4, gross bleeding), and weight loss (0, none; 1, 0–5%; 2, 5–10%; 3, 10–20%; 4, > 20%) (25). After the mice were euthanized, the spleen, thymus, liver, kidney, colon, and colon were collected for further analysis.

### Assessment of Disease Activity Index (DAI) Score

Stool consistency, body weight, and blood in the stool were determined according to a previously published grading system (26) to assess the severity of colitis. Briefly, blood in stool was scored as follows: score 0, normal; score 2, slight bleeding; and score 4, gross bleeding. Diarrhea was scored as follows: score 0, normal; score 2, loose stools; and score 4, watery diarrhea. Weight loss was scored as follows: score 0, none; score 1, 1–5%; score 2, 5–10%; score 3, 10–20%; and score 4, >20%.

### Cell Culture and Viability Assay

The IPEC-J2 cells obtained from the Guoqiang Zhu of Yangzhou University (Yangzhou, China) were grown at 37°C in a humidified % CO_2_ incubator in DMEM/F12 supplemented with 10% FBS and 1% penicillin/streptomycin. An IPEC-J2 cell viability experiment was carried out using the Cell Counting Kit-8. Briefly, IPEC-J2 cells were scattered at density 1 × 10^4^ cells/mL in 96-well plates and were treated with BTL-1 (10, 20, 50, and 100 μM) for 24 h. After that, 10 μL CCK-8 solution was added and incubated for another 1 h. The optical density at 450 nm was used to measure the cell viabilities using a microplate reader (BioTek, Vermont, USA).

### Cytokine Assay

IPEC-J2 cells were seeded at a density of 5 × 10^6^ cells/well in 6-well plates to investigate the inhibitory effect of BTL-1 on inflammatory cytokines. The cells were pretreated for 24 h with BTL-1 (10, 20, and 50 μM) and then activated for 1 h with LPS (50 μg/mL). The tissue waste (culture medium) was collected and centrifuged at 3,000 rpm for 10 min. Serum was treated the same as the culture medium. For cytokine detection, colon tissue was ground thoroughly and centrifuged at 3,000 rpm for 10 min. The level of IL-1β, TNF-α, IL-6, and IL-10 in the culture medium, serum, and colonic tissue were quantified using ELISA kits (Meimian, Shanghai, China).

### Western Blotting Analysis

Protein levels were measured using a BCA reagent after IPEC-J2 cells, and colonic tissues were homogenized (Cwbio, Beijing, China). According to the manufacturer's recommendations, the cytoplasmic and nuclear extractions were carried out (keygen, Jiangsu, China). SDS-PAGE was used to load and electrophorese the samples, which were subsequently transferred through polyvinylidene fluoride (PVDF) membranes. After 30 min of blocking in QuickBlock™ Blocking Buffer (Beyotime, Shanghai, China), the membranes were incubated with primary antibodies. The following primary antibodies were used: TLR4, P65, phospho-P65, IκBα, phospho-IκBα, phospho-ERK1/2, ERK 1/2, phospho-P38, P38, phospho-JNK, JNK, occludin, ZO-1, claudin1, and β-actin at 1:1,000 dilution. Secondary antibodies include horseradish peroxidase (HRP)-conjugated goat anti-rabbit, and anti-mouse IgG at 1:1,000 dilution (Cell Signaling Technology, Danvers, MA, USA).

### Total RNA Extraction and qPCR

RNA was extracted using TRIZOL followed by cDNA synthesis *via* a reverse transcriptase. Quantitative RT-PCR analyses were performed using SYBR Green (TransGen Biotech, Beijing, China). The results were evaluated using the 2–^ΔΔ^CT method and normalized to β-actin expression. The following primers were used (Table 1).

**Table 1:** Primer sequence.

**Gene ID**	**Primer**	**Sequence (5'-3')**
414396	pig β-actin Forward	GCTGTCCCTGTATGCCTCT
	pig β-actin Reverse	GATGTCACGCACGATTTCC
399500	pig IL-6 Forward	ATAAGGGAAATGTCGAGGCTGTGC
	pig IL-6 Reverse	GGGTGGTGGCTTTGTCTGGATTC
397122	pig IL-1β Forward	CAAGCCAGAGAAGCAAGGTGTCC
	pig IL-1β Reverse	GCCGTCCTCAGCAGCAAGAAG
397106	pig IL-10 Forward	AGCCAGCATTAAGTCTGAGAACAGC
	pig IL-10 Reverse	GGTCAGCAACAAGTCGCCCATC
397086	pig TNF-α Forward	AAAGGACACCATGAGCACGGAAAG
	pig TNF-α Reverse	CGCCACGAGCAGGAATGAGAAG
399541	pig TLR4 Forward	GCCATCGCTGCTAACATCATC
	pig TLR4 Reverse	CTCATACTCAAAGATACACCATCGG
100135665	pig P65 Forward	CTGAGGCTATAACTCGCTTGGTGAC
	pig P65 Reverse	CATGTCCGCAATGGAGGAGAAGTC
100157586	pig IκB Forward	GGCAGTGACATGAGTGGCAGATAC
	pig IκB Reverse	CTTCCTTGAGGCTGTGCTTCTTCC
445013	pig ERK1 Forward	ACGTCATTGGCATCCGAGACATTC
	pig ERK1 Reverse	GAGGAAGTAGCAGATGTGGTCGTTG
100626611	pig P38 Forward	CGCCTGTGAAGACCTCCTTGAAC
	pig P38 Reverse	TTCCTGTCCTCCACCTTCCGAAG
396610	pig JNK Forward	ACTACAGAGCACCTGAGGTCATCC
	pig JNK Reverse	ATTTCTCCCATAATGCACCCCACAG
397236	pig Occludin Forward	CAGTGGTAACTTGGAGGCGTCTTC
	pig Occludin Reverse	CGTCGTGTAGTCTGTCTCGTAATGG
100736682	pig ZO-1 Forward	CCAGGGAGAGAAGTGCCAGTAGG
	pig ZO-1 Reverse	TTTGGTGGGTTTGGTGGGTTGAC
100625166	pig Claudin1 Forward	AGAAGATGCGGATGGCTGTCATTG
	pig Claudin1 Reverse	ACCATACCATGCTGTGGCAACTAAG
11461	mouse β-actin Forward	GTGACGTTGACATCCGTAAAGA
	mouse β-actin Reverse	GCCGGACTCATCGTACTCC
16193	mouse IL-6 Forward	CTCCCAACAGACCTGTCTATAC
	mouse IL-6 Reverse	CCATTGCACAACTCTTTTCTCA
16176	mouse IL-1β Forward	GAAATGCCACCTTTTGACAGTG
	mouse IL-1β Reverse	TGGATGCTCATCAGGACAG
16153	mouse IL-10 Forward	TTCTTTCAAACAAAGGACCAGC
	mouse IL-10 Reverse	GCAACCCAAGTAACCCTTAAAG
21926	mouse TNF-α Forward	ATGTCTCAGCCTCTTCTCATTC
	mouse TNF-α Reverse	GCTTGTCACTCGAATTTTGAGA
21898	mouse TLR4 Forward	GCCATCATTATGAGTGCCAATT
	mouse TLR4 Reverse	AGGGATAAGAACGCTGAGAATT
19697	mouse P65 Forward	TGCGATTCCGCTATAAATGCG
	mouse P65 Reverse	ACAAGTTCATGTGGATGAGGC
12675	mouse IκB Forward	TTGGGTTATGCCAAAGATGTTG
	mouse IκB Reverse	GCTGTGTACGGCTTATTTTCAA
26417	mouse ERK1 Forward	CAGCTCAACCACATTCTAGGTA
	mouse ERK1 Reverse	TCAAGAGCTTTGGAGTCAGATT
26416	mouse P38 Forward	AGGAATTCAATGACGTGTACCT
	mouse P38 Reverse	AGGTCCCTGTGAATTATGTCAG
26419	mouse JNK1 Forward	TTGAAAACAGGCCTAAATACGC
	mouse JNK1 Reverse	GTTTGTTATGCTCTGAGTCAGC
18260	mouse Occludin Forward	TGCTTCATCGCTTCCTTAGTAA
	mouse Occludin Reverse	GGGTTCACTCCCATTATGTACA
21872	mouse ZO-1 Forward	CTGGTGAAGTCTCGGAAAAATG
	mouse ZO-1 Reverse	CATCTCTTGCTGCCAAACTATC
12737	mouse Claudin1 Forward	AGATACAGTGCAAAGTCTTCGA
	mouse Claudin1 Reverse	CAGGATGCCAATTACCATCAAG

### Immunofluorescence

IPEC-J2 cells were seeded on slide cover glass at 5 × 10^5^ cells/mL, fixed in 4% paraformaldehyde at 4°C for 30 min, and then rinsed with phosphate-buffered saline (PBS). Cells were permeabilized with 0.2 % Triton-X-100, then rinsed with PBS before being blocked with 5% BSA. After that, the cells were incubated overnight with primary antibodies *p*-P65 and ZO-1 at 1:100 dilution and incubated with a biotin-conjugated anti-Rabbit IgG antibody for 1 h. Afterward, the cells were washed with PBS and treated for 1 h with DyLight 594-conjugated Avidin. Fluorescence microscopy was used to examine the nuclei stained with DAPI (Olympus, Tokyo, Japan).

### *In vitro* Intestinal Paracellular Permeability Assay

The IPEC-J2 cells were seeded onto transwell at a density of 2 × 10^5^ cells/mL and reached confluence. Cells were cultured for 16 days before use. Then, IPEC-J2 cells were washed three times with PBS to remove the antibiotic media. The treatment is the same with section Induction of Experimental Colitis. The Trans Epithelial Electric Resistance (TEER) was measured by a transmembrane resistance meter (MRRCK, NJ, USA). After TEER was measured, barrier function was determined by upper compartment to lower compartment flux of 4 kDa fluorescein isothiocyanate-labeled dextran (FITC-dextran). The upper compartment was filled with FITC-dextran (0.5 mL, 1 mg/mL) (27), and 1 h later, the concentrations of FITC-dextran in the lower compartment were determined using a fluorometer (Thermo, MA, USA; excitation, 427 nm; emission, 536 nm).

### *In vivo* Intestinal Permeability Assay

This assay was based on the intestinal permeability toward 4 kDa FITC-dextran as described elsewhere (28). Briefly, rats fasted for 8 h were given 4 kDa FITC-dextran (MRRCK, NJ, USA) by gavage (40 mg/100 g body weight, 100 mg/mL). The blood was collected from the submandibular venous plexus 4 h later and centrifuged at 4°C at 4,000 rpm for 10 min. Plasma was diluted with an equal volume of PBS (pH 7.4) and analyzed for FITC-dextran concentration using a fluorometer (Thermo, MA, USA; excitation, 427 nm; emission, 536 nm).

### H&E Staining

The colon was embedded in paraffin, sectioned, and placed on a microscope slide after being preserved in 4% phosphate-buffered paraformaldehyde. Hematoxylin and eosin (H & E) were used to stain the slides, and the histopathological changes were evaluated using an optical microscope (Olympus, Tokyo, Japan).

### Molecular Docking

We used Chembiodraw Ultra 14.0 to draw the structure of BTL-1. After that, we used Chembio3d Ultra 14.0 to transform it into a 3D structure and optimized it with an mmff94 force field. 3D structure of Toll-like receptor (TLR4) (PDB ID: 2z64) was obtained from RCSB protein data bank (www.rcsb.org). Both TLR4 and BTL-1 were transformed into pdbqt format by autodock tools 1.5.6 (29, 30). In this project, autodock Vina 1.1.2 (31) was used for molecular docking. The coordinate of TLR4 active site is: Center_ x = −28.441, center_ y = −17.705, center_ z = −22.582, size_ x = 20, size_ y = 20, size_ z = 20. To increase the accuracy of the calculation, we set the parameter exhaust to 100. Except for special instructions, other parameters use default values. Finally, the conformation with the highest score was selected and analyzed with PyMOL 1.7.6.

### Identification of BTL-1

Butyrolactone-I was obtained in the form of yellow oil after repeated column chromatography of the extract. Its NMR and specific optical rotation data listed below are identical to previous reports (14, 32).

**Figure 2:**
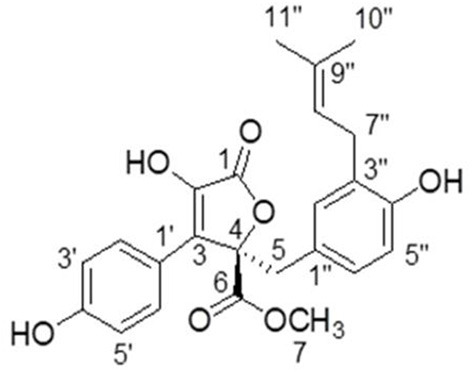
The structure of butyrolactone-I.

^**1**^**H NMR** (CD_3_OD, 700 M*Hz*): δ_H_ 7.59 (H-2'and H-6', d, 8.4), 6.87 (H-3' and H-5', d, 9.1), 6.54 (H-6”, dd, 8.1, 2.1), 6.50 (H-5”, d, 8.4), 6.41 (H-2”, d, 2.1), 5.07 (H-8”, m), 3.78 (H-7, s),3.43 (H-5, d, 14.0), 3.08 (H-7”, m), 1.67 (H-10”, s), 1.57 (H-11”, s). ^**13**^**C-NMR NMR** (CD_3_OD, 175 M*Hz*):δ_C_ 171.41 (C-6), 170.11 (C-1), 159.13 (C-4'), 154.88 (C-4”), 139.46 (C-2), 132.78 (C-9”), 132.19 (C-2”),130.19 (C-2' and C-6'), 129.56 (C-3”), 129.01 (C-3), 128.23 (C-6”), 124.85 (C-1”), 123.36 (C-8”),122.94 (C-1'), 116.41 (C-3' and C-5'), 114.83 (C-5”), 86.59 (C-4), 53.64 (C-7), 39.41 (C-5), 28.48 (C-7”), 25.75 (C-10”), 17.56 (C-11”) (Figure 2). [α]${\left[\alpha \right]}_{D}^{20}$ 108.85°(c 0.2, MeOH).

### Statistical Analyses

All experimental results were obtained from at least three independent experiments. For statistical analyses, the values are expressed as mean ± SD (*n* = 3). One-way ANOVA and *t*-test determined the statistical significance. A value of *P* < 0.05 was considered statistically significant. IBM SPSS Statistics 26 was used for all statistical analyses, and GraphPad Prism 7.0 (GraphPad Software) was used for all column and line charts.

## Results

### BTL-1 Decreases IL-6 and IL-1β and Increases TNF-α in IPEC-J2 Exposed to LPS

**Figure 3:**
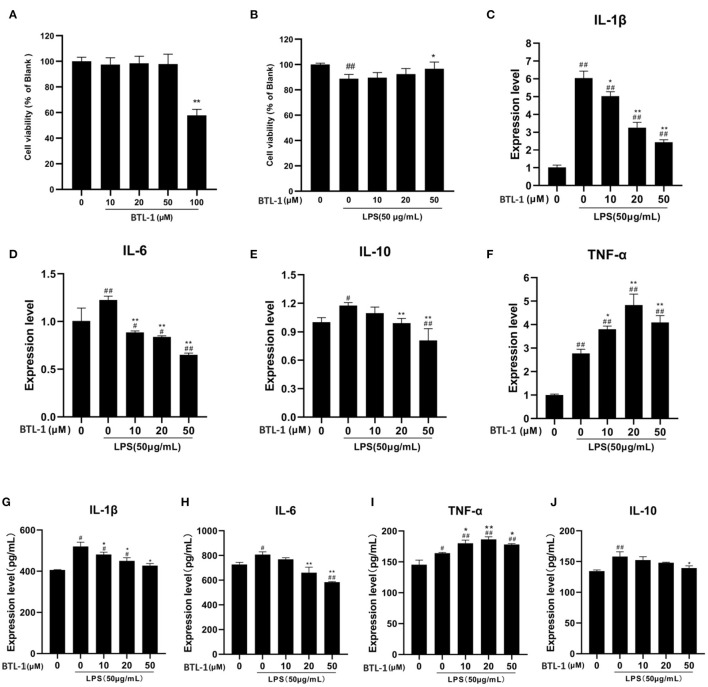
Impact of BTL-1 on inflammatory cytokines production in LPS-induced IPEC-J2 cell. The outcome of BTL-1 on IPEC-J2 cell viability **(A,B)**. The consequences of BTL-1 on the expression of cytokines were determined by ELISA **(C–F)** and qPCR **(G–J)**. The consequences of BTL-1 on the expression of cytokines were determined by ELISA. The cells were treated with BTL-1 (10, 20, and 50 μM) for 24 h and then treated with LPS (50 μg/mL) for another 1 h. The data shown are representative of three independent experiments. The results were expressed as the means ± SEM. ^#^*P* < 0.05, ^##^*P* < 0.01, compared to non-treated group, ^*^P < 0.05, ^**^P < 0.01 compared to LPS group.

The toxicity of BTL-1 on IPEC-J2 cells was first examined by CCK8 assay. After treatment with several concentrations of BTL-1 for 24 h, the cell proliferation rate was dramatically decreased when exposed to 100 μM BTL-1; thereby, the highest concentration for *in vivo* study was set at 50 μM (Figure 3A). Three concentrations (10, 20, and 50 μM) were used in subsequent experiments. As shown in Figure 3B, LPS considerably decreased the cell proliferation rate of IPEC-J2, whereas BTL-1 increased that in a dose-dependent manner. Further, LPS significantly upregulates the expression of IL-6, IL-10, IL-1β, and TNF-α at mRNA and protein levels, whereas BTL-1 decreased IL-10 and IL-1β in a dose-dependent way (Figures 3C–J). Interestedly, BTL-1 significantly increased the expression of TNF-α. In short, these findings suggest that BTL-1 may have a protective impact on the IPEC-J2 cell model of LPS-induced inflammation.

### BTL-1 Inhibits TLR4/NF-κB Signaling Pathway in IPEC-J2 Cells Exposed to LPS

**Figure 4:**
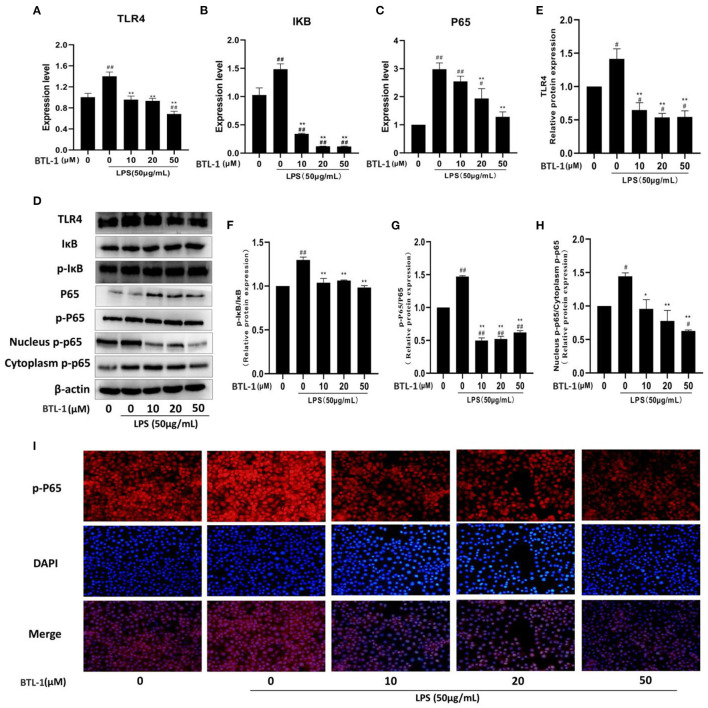
Inhibitory effects of BTL-1 on TLR4/NF-κB signaling pathway. BTL-1 inhibits TLR4/NF-κB signaling pathway as determined by qPCR **(A–C)** and western blotting **(D–G)**. BTL-1 inhibits the translocation of phosphorylated P65 as determined by western blotting **(D,H)** and IF **(I)**. The data are representative of three independent experiments. The results were expressed as the means ± SEM. ^#^*P* < 0.05, ^##^*P* < 0.01, compared to non-treated group, ^*^P < 0.05, ^**^P < 0.01 compared to LPS group.

TLR4, IκB, P65, and the phosphorylated IκB and P65 were measured to evaluate the sequel of BTL-1 on the TLR4/NF-κB signaling pathway. As shown in Figures 4A–C, the mRNA expressions of TLR4, p65, and IκB were significantly upregulated after LPS treatment. However, they have downregulated in a dose-dependent manner after BTL-1 pretreatment. Meanwhile, when the cells were exposed to LPS, TLR4 and the phosphorylated P65 and IκB were greatly enhanced; however, they were significantly decreased when the cells were pretreated with BTL-1 (Figures 4D–G).

Western blotting and IF measured translocation of phosphorylated P65 from the cytoplasm into the nucleus. As shown in Figures 4D,H, LPS significantly increased the translocation of phosphorylated P65 from the cytoplasm to the nucleus; however, the translocation was inhibited when the cells were pretreated with BTL-1. In IPEC-J2 cells, immunofluorescence was used to observe the site of phosphorylated P65. In the blue channel, the nucleus was stained with DAPI, and the phosphorylated P65 was stained red. The phosphorylated P65 was found outside the nucleus with weak immunofluorescence in the control group. Still, the immunofluorescence became brighter and approached the nucleus when the cells were exposed to LPS, suggesting that LPS enhances translocation of phosphorylated P65 from the cytoplasm to the nucleus. When cells were pretreated with BTL-1, the translocation of phosphorylated P65 was inhibited. The above results denote that BTL-1 can inhibit the TLR4/NF-κB signaling pathway by downregulating the expression of TLR4, the phosphorylation of IκB and P65, and reduces the translocation of phosphorylated P65 (Figure 4I).

### BTL-1 Upregulates TNF-α *via* MAPK Signaling Pathway in IPEC-J2 Exposed to LPS

**Figure 5:**
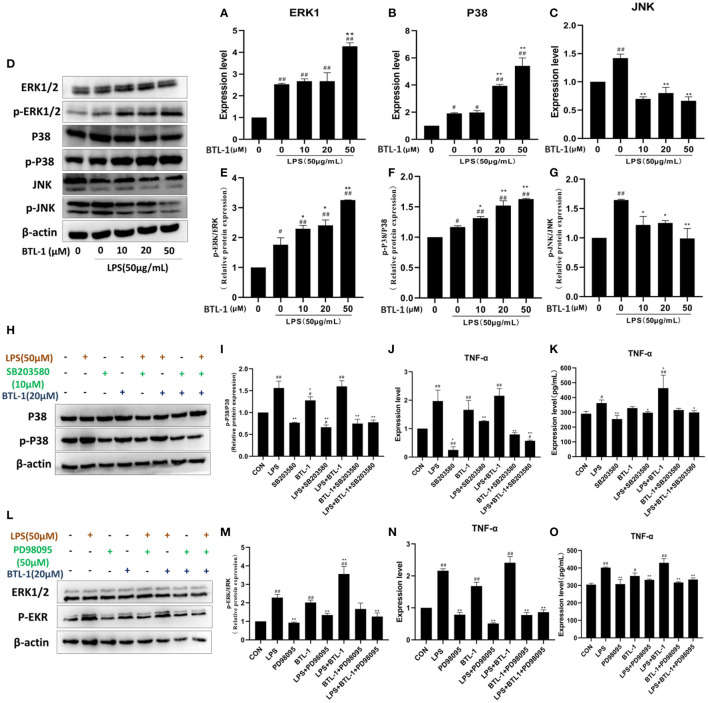
Effect of BTL-1 on MAPK signaling pathway. The impact of BTL-1 on the MAPK signaling pathway determined by qPCR **(A–C)**. The outcome of BTL-1 on the MAPK signaling pathway determined by western blotting **(D–G**). Effect of P38 inhibitor (SB203580, 10 μM, 1 h) on the phosphorylation of P38. **(H,I)** determined by western blotting. Impact of P38 inhibitor on the mRNA and protein of TNF-α determined by qPCR and ELISA. **(J,K)**. The outcome of ERK1/2 inhibitor (PD98095,50 μM, 1 h) on the phosphorylation of ERK1/2 **(L,M)** determined by western blotting analysis. Inhibitory effect of ERK1/2 inhibitor on the mRNA and protein of TNF-α determined by qPCR and ELISA **(N,O)**. The data shown are representative of three independent experiments. The inhibitors of ERK1/2 and P38 were added 1 h before LPS and BTL-1 treatment, and the cells were washed with PBS three times before adding LPS and BTL-1. The results were expressed as the means ± SEM. ^#^*P* < 0.05, ^##^*P* < 0.01, compared to non-treated group, ^*^P < 0.05, ^**^P < 0.01 compared to LPS group.

P38, ERK1/2, JNK, and the phosphorylation of P38, ERK1/2, and JNK were tested to assess the impact of BTL-1 on the MAPK signaling pathway. As shown in Figures 5A–C, the mRNA expressions of P38, ERK1, and JNK were significantly upregulated after LPS treatment. However, after treatment with BTL-1, the mRNA expression levels of ERK1 and P38 were further upregulated. In contrast, JNK was significantly downregulated after BTL-1 treatment. Additionally, the western blotting analysis showed that the phosphorylation of P38, ERK1/2, and JNK was dramatically increased when the cells were exposed to LPS. In contrast, phosphorylation of ERK1 and P38 were significantly decreased following BTL-1 treatment (Figures 5D–G). On the contrary, the phosphorylated JNK was downregulated considerably after BTL-1 treatment.

ERK1/2 and P38 inhibitors were added to the IPEC-J2 cell to investigate the mechanism contributing to increased TNF-α after BTL-1 treatment. As shown in Figures 5H,I, the phosphorylation of P38 was significantly inhibited by adding a P38 inhibitor (SB203580,10 μM). Meanwhile, the mRNA and protein expression of TNF-α was decreased when SB203580 was added (Figures 5J,K). Same as phosphorylated P38, the activity of phosphorylated ERK1/2 was reduced after adding inhibitor PD98095 (Figures 5L–O). Moreover, the mRNA and protein expression of TNF-α was significantly decreased after inhibition of ERK1/2 phosphorylation. The above findings indicate that the expression of TNF-α was closely associated with the phosphorylation of P38 and ERK1/2.

### BTL-1 Protects Intestinal Paracellular Permeability *via* Upregulating Tight Junction Proteins

**Figure 6:**
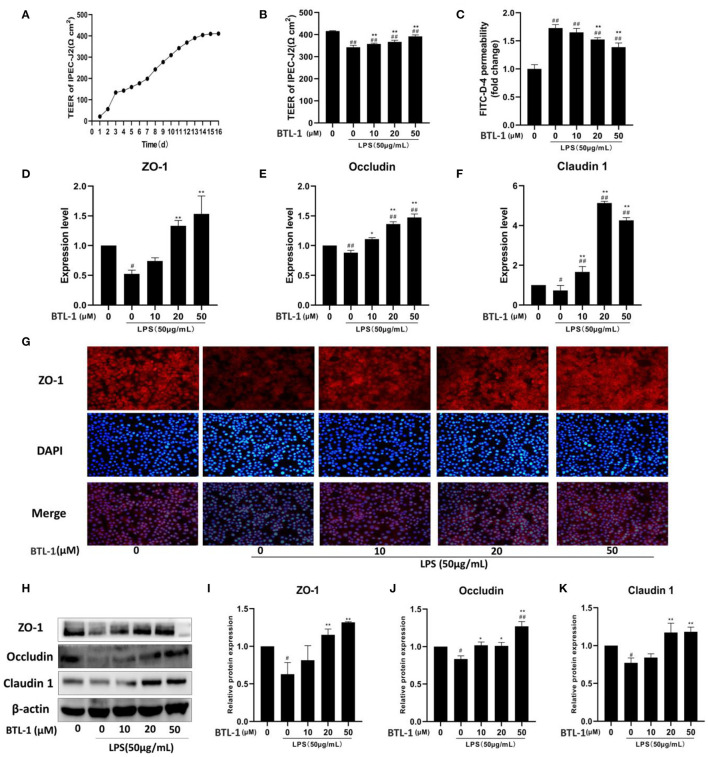
*In vitro* effect of BTL-1 on intestinal paracellular permeability. Transmembrane resistance of IPEC-J2 monolayer measured by IPEC-J2 cell resistance voltmeter **(A)**. The effect of BTL-1 on transmembrane resistance *in vitro* intestinal paracellular permeability was measured by a fluorometer **(B)**. The effect of BTL-1 on FITC-D fluorescence intensity in the lower compartment measured by a fluorescence microplate reader **(C)**. The effect of BTL-1 on TJ protein determined by qPCR **(D–F)**, IF **(G)**, and western blotting **(H–K)**. The data are representative of three independent experiments. The results were expressed as the means ± SEM. ^#^*P* < 0.05, ^##^*P* < 0.01, compared to non-treated group, ^*^P < 0.05, ^**^P < 0.01 compared to LPS group.

From the finding mentioned above, BTL-1 could exert an anti-inflammatory effect. Subsequently, we evaluated the intestinal barrier protection function of BTL-1 *in vitro*. The transepithelial resistance was stable on 14–16th day after cell culture (Figure 6A), followed by BTL-1 treatment and LPS stimulation. As shown in Figures 5B,C, exposure to LPS significantly decreased transmembrane resistance (TEER), 1 h post-exposure (Figure 6B). In addition, it increased the passage of FITC-dextran (FITC-D) (Figure 6C), indicating that LPS significantly impaired epithelial barrier integrity. Treatment of IPEC-J2 cells with BTL-1 significantly increased TEER and decreased the paracellular flux of FITC-D in a dose-dependent manner.

Subsequently, the expression of TJ proteins was determined. As shown in Figures 6D–F, the mRNA expressions of ZO-1, occludin, and claudin1 were significantly downregulated after LPS treatment; however, they were upregulated following BTL-1 treatment. Meanwhile, when the cells were exposed to LPS, ZO-1, occludin, and claudin1 were significantly reduced; nevertheless, they significantly increased when treated with BTL-1 (Figures 6H–K) protein level. The immunofluorescence analysis showed that ZO-1 expression was decreased after LPS treatment (Figure 6G). However, when the cells were treated with BTL-1, ZO-1 expression was significantly enhanced. These results suggest that LPS may inhibit the expression of tight junction proteins, thereby increasing intestinal paracellular permeability. In contrast, BTL-1 can increase the expression of TJ proteins in a dose-dependent manner to protect the integrity of the intestinal barrier.

### BTL-1 Protects DSS-Induced IBD in Mice

**Figure 7:**
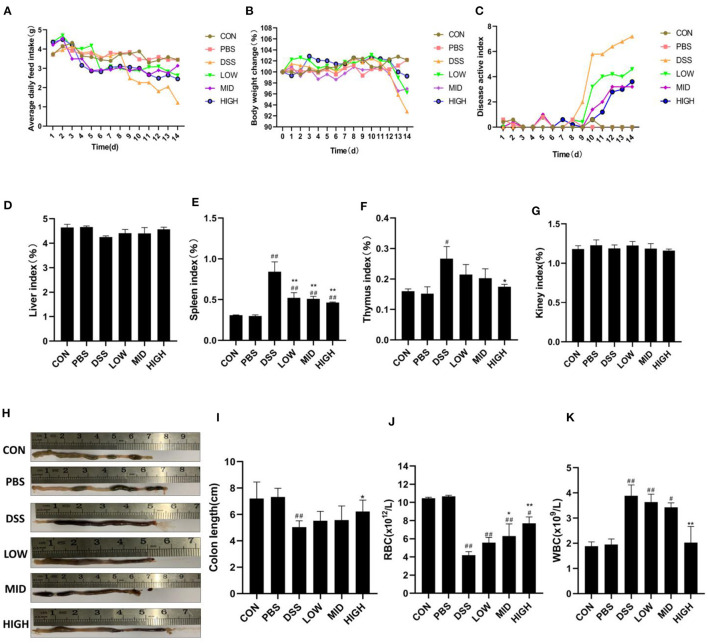
The effect of BTL-1 on DSS-induced IBD model in mice. Mice were given DSS for 7 days with or without BTL-1, the PBS group was given only PBS, and the control group was fed normally. The effect of BTL-1 on average daily feed intake **(A)**, average daily gain **(B)**, DAI **(C)**, organ index **(D–G)**, and colon **(H,I)** of IBD mouse model induced by DSS. The effects of BTL-1 on blood routine examination in DSS-induced IBD mice **(J,K)**. The results were expressed as the means ± SEM of *n* = 5. ^#^*P* < 0.05, ^##^*P* < 0.01, compared to control group, ^*^P < 0.05, ^**^P < 0.01 compared to DSS group.

Previous experiments confirmed that BTL-1 has a protective effect on the LPS-induced inflammation model in IPEC-J2 cells. Subsequently, we examined the impact of BTL-1 on the DSS-induced IBD model in mice. Before DSS treatment, intragastric administration of BTL-1 reduced the average daily feed intake (Figure 7A) of mice; however, it did not affect the body weight (Figure 7B) and disease activity index (DAI) (Figure 7C). After DSS treatment, the average daily gain of mice decreased significantly. Further, the symptoms of wilting, loose stool, bloody stool, and anemia (Figure 7J) were appeared, leading to the increase in the DAI. At the same time, DSS significantly increased the immune organ index (thymus index and spleen index) (Figures 7D–G) and the total number of white blood cells (Figure 7K), decreased the length of the colon (Figures 7H,I). Thus, BTL-1 could rescue the symptoms and pathological changes caused by DSS in a dose-dependent manner compared with the DSS group. These results denote that BTL-1 has a potential protective effect on DSS-Induced IBD of mice.

### BTL-1 Inhibits the Expression of Inflammatory Factors in DSS Induced IBD of Mice

**Figure 8:**
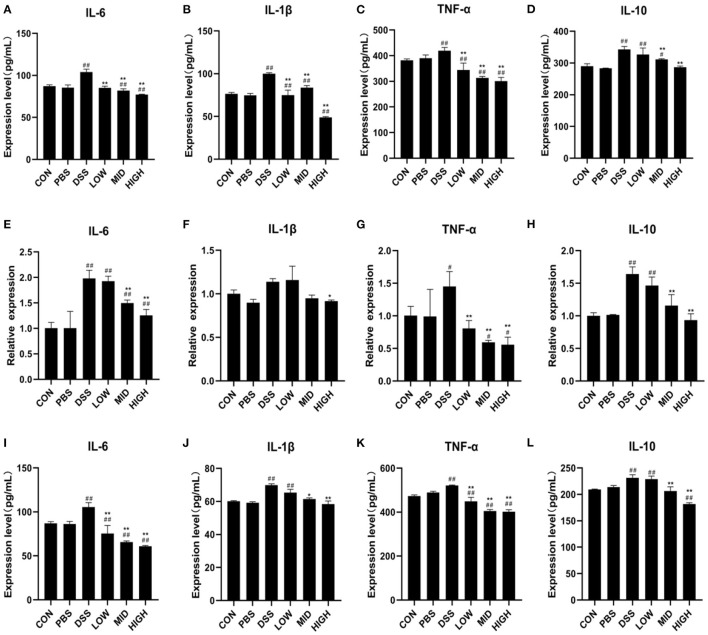
The effects of BTL-1 on blood routine examination and expression of inflammatory factors in DSS-induced IBD mice. The effects of BTL-1 on inflammatory factors in serum **(A–D)** and intestinal tissue as measured by qPCR **(D–H)** and ELIS A **(I–L)**. The results were expressed as the means ± SEM of *n* = 3. ^#^*P* < 0.05, ^##^*P* < 0.01, compared to control group, ^*^P < 0.05, ^**^P < 0.01 compared to DSS group.

The expression of inflammatory factors in the blood and intestinal tissues were measured to investigate the impacts of BTL-1 on blood indexes and intestinal inflammatory factors in the DSS-induced IBD mice model. Consistent with blood routine results, the ELISA results of serum inflammatory factors (Figures 8A–D) showed that DSS treatment results in a measurable increase in serum inflammatory factors (IL-10, IL-6, TNF-α, and IL-1β), and BTL-1 would significantly inhibit such increases. Furthermore, the changes in the mRNA and concentration of inflammatory factors in the intestinal tissue also showed the same trend (Figures 8E–L). These results suggest that DSS treatment can upregulate the inflammatory factors in mice and the occurrence of intestinal inflammation. At the same time, BTL-1 can effectively inhibit the expression of inflammatory factors, in turn, inflammation.

### BTL-1 Inhibits the Activity of TLR4/NF-κB and MAPK Signaling Pathways in the DSS-Induced IBD Model

**Figure 9:**
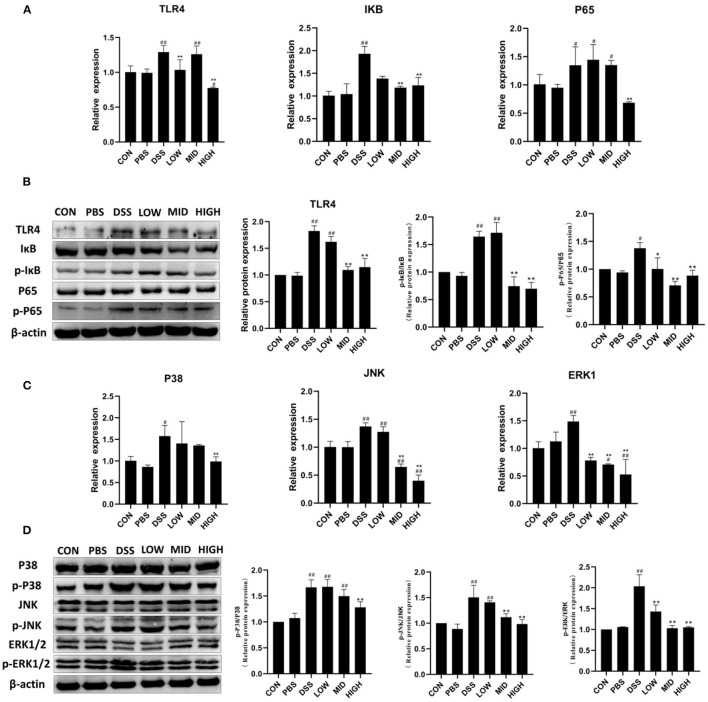
The effect of BTL-1 on the intestinal NF-κB and MAPK signaling pathways of IBD mouse model induced by DSS. The inhibitory effect of BTL-1 on TLR4/NF-κB signaling pathway determined by qPCR **(A)** and western blotting **(B)**. The inhibitory effect of BTL-1 on the MAPK signaling pathway determined by qPCR **(C)** and western blotting **(D)**. The results were expressed as the means ± SEM of *n* = 3. ^#^*P* < 0.05, ^##^*P* < 0.01, compared to control group, ^*^P < 0.05, ^**^P < 0.01 compared to DSS group.

The critical proteins of NF-κB and MAPK signaling pathways were measured by qPCR and WB to investigate the mechanism of BTL-1 inhibiting inflammation in the DSS-induced IBD mouse model. As shown in Figures 9A,B, DSS treatment increased TLR4, IκB, and P65's mRNA expression and upregulated the protein expression of TLR4 and the phosphorylation of IκB and P65. However, the expression and phosphorylation of key proteins (TLR4, IκB, and P65) of the NF-κB pathway were inhibited after BTL-1 was administered intragastrically. Furthermore, unlike the IPEC-J2 cell experiment, BTL-1 also significantly inhibited the phosphorylation of ERK, P38, and JNK in mice induced by DSS. In sum, the TNF-α in mice was downregulated considerably, indicating that BTL-1 regulates the expression of TNF-α through phosphorylation of ERK and P38 (Figures 9C,D).

### BTL-1 Alleviates DSS-Induced Intestinal Injury in Mice by Upregulating TJ Proteins

**Figure 10:**
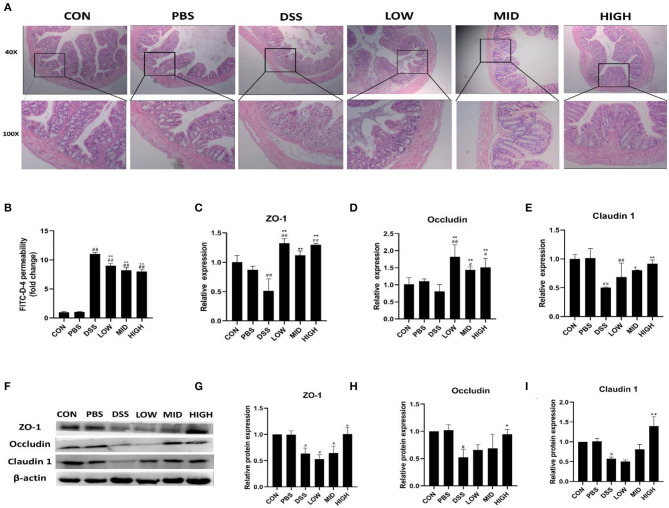
The effect of BTL-1 on the intestinal injury of IBD mouse model induced by DSS. Histopathological changes after DSS stimulation in the colon **(A)**. The *in vitro* intestinal barrier function evaluated by FITC-D fluorescence intensity **(B)**. Upregulation of BTL-1 on tight junction proteins as determined by qPCR **(C–E)** and western blotting **(F–I)**. The results were expressed as the means ± SEM of *n* = 3. ^#^*P* < 0.05, ^##^*P* < 0.01, compared to control group, ^*^P < 0.05, ^**^P < 0.01 compared to DSS group.

Intestinal permeability, intestinal structure, and tight junction proteins were measured to investigate the effect of BTL-1 on intestinal injury in the DSS-induced IBD mice model. As shown in Figure 10B, the intestinal permeability was increased after DSS treatment, and the serum FITC-D fluorescence intensity was significantly increased. Although the permeability of the intestine was not restored to normal levels following treatment with BTL-1, it is greatly improved compared with the DSS group. As shown in Figure 10A, there was no apparent colon injury in the control and PBS groups. On the contrary, the mucosa structure in the DSS group was not clear, and many inflammatory cells infiltrated in lamina propria and submucosa, accompanied by submucosal edema. Compared with the DSS group, the structural damage, inflammatory cell infiltration, and submucosal edema of each dose of the BTL-1 group were all improved. We also measured the expression of TJ proteins, as shown in Figures 10C–I; DSS treatment decreased the mRNA and proteins expression of ZO-1, occludin, and claudin1. However, the expression of TJ proteins (ZO-1, occludin, and claudin1) was upregulated after BTL-1 was administered intragastrically.

### Docking of BTL-1 With TLR4

**Figure 11:**
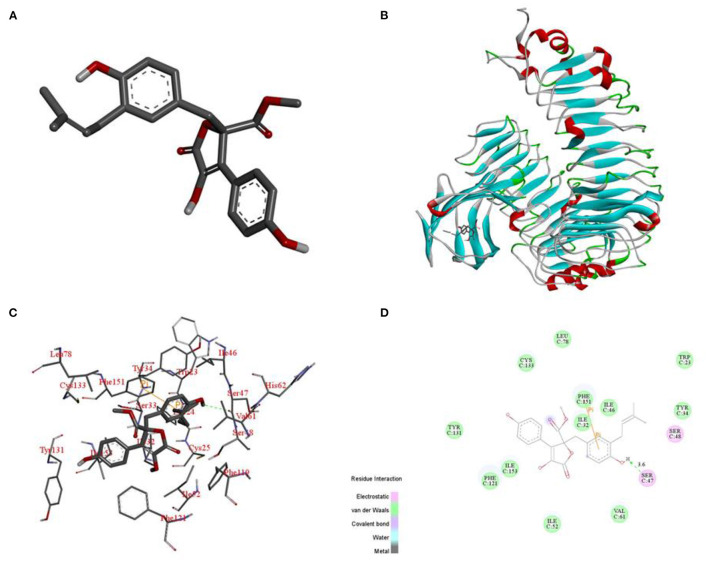
Binding mode of BTL-1 into the TLR4. 3D structure of BTL-1 **(A)**. Docking of BTL-1 to the active pocket of TLR4 (overall) **(B)**. Docking of BTL-1 to the active pocket of TLR4 (detail) **(C)**. Docking of BTL-1 to the active pocket of TLR4 (2D) **(D)**.

To elucidate the interaction mode between TLR4 and compound BTL-1 at the molecular level, we docking compound BTL-1 to the active pocket of TLR4 with an affinity of −8.8 kcal mol−1. The 3D structure of BTL-1 is shown in Figure 11A. The theoretical binding mode is shown in Figures 11B,C. It can be seen from Figure 11B that the active pocket of compound butyrolactone-I presents a compact binding mode. It can be seen from Figures 11B,C that BTL-1 is in a cavity composed of amino acids Ile-32, Ile-46, Ser-47, Ile-52, Val-61, Leu-78, Phe-121, Cys-133, Phe-151, and Ile-153, forming a robust hydrophobic interaction. It is crucial that one hydroxyl group of BTL-1 can form a hydrogen bond with amino acid Ser-47 to 3.6 Å (Figure 11D) and form a Pi-Pi bond with amino acid Phe-151, which is the most critical force between butyrolactone I and TLR4. All these interactions make BTL-1 and TLR4 form stable complexes. In conclusion, the above docking studies provide a reasonable explanation for the interaction between BTL-1 and TLR4.

## Discussion

The incidence rate of IBD has been increasing worldwide, which seriously threatens human health. Even though the pathogenic mechanism of IBD is still unknown, current data suggests that IBD symptoms are caused by abnormal immune responses and damage to the intestinal barrier. Therefore, the development of effective anti-inflammatory and intestinal barrier protective function agents may be a future treatment strategy for IBD (33). This study first reported that butyrolactone-I alleviates LPS induced inflammatory response in IPEC-J2 *via* TLR4/NF-κB and MAPK signaling pathways. Therefore, BTL-1 can be used as an ocean drug to prevent intestinal bowel disease.

Butyrolactone-I was first reported in 1977 (34). Butyrolactone-I has a furan ring system and two phenolic groups in terms of structure. Many studies have shown that phenolic compounds can inactivate toll-like receptors, NF-κB, MAPK, kappa kinase/c-Jun amino-terminal kinases (IKK/JNK), inhibiting pro-inflammatory genes expression (35, 36). Similarly, natural furan derivatives have many pharmacological activities, such as antibacterial, antioxidant, antiviral, anti-inflammatory, anti-tumor, analgesic, anti-convulsant, and anti-fungal effects (32, 37). Furthermore, it has been proved that butyrolactone-I has the functions of inhibiting mammalian oocyte division, inhibiting tumor cell proliferation, inducing cancer cell apoptosis, preventing neuron apoptosis, has antibacterial and anti-inflammatory effects on microglia (14, 16, 18, 21, 22, 38). However, there is still a gap in demonstrating its ability to inhibit intestinal inflammation and protect intestinal function at the cellular and animal levels.

Given the LPS-induced IPEC, the cell inflammation and DSS-induced IBD mouse models are widely accepted as experimental models for assessing intestinal inflammation and IBD (39). We used IPEC-J2 cells to create an LPS-induced inflammatory model *in vitro* and found IL-1, IL-6, and TNF- elevated. Meanwhile, after being exposed to LPS, cell viability was reduced. In *in vivo* study, DSS administration significantly reduced the body weight gain and increased the disease activity index in mice, denoting that the experimental model was successfully established.

Toll-like receptors play an essential role in innate immunity. It can recognize characteristics that are shared by many viruses and their pathogenic components (PAMP) and trigger a natural immune response. LPS first activates TLR4 to trigger the downstream of the MAPK and NF-κB signaling pathways, thereby regulating cytokine production and the expression of many inflammatory genes. In IPEC-J2, we found that BTL-1 could inhibit the phosphorylation and nuclear entry of P65 *via* inhibiting TLR4, thereby decreasing the expression of IL-1β and IL-6. Studies have pointed out that BTL-1 has an active site that binds to P65 (14), so it is not ruled out that BTL-1 can also directly bind to P65, inhibiting the expression of downstream inflammatory factors. At the same time, BTL-1 can regulate the expression of TNF-α by upregulating the phosphorylation of ERK1/2 and P38 when TLR4 was inhibited. In line with our results, studies have pointed out that ERK and P38 can regulate the expression of TNF-α (40). Unfortunately, we cannot explore how BTL-1 upregulates the phosphorylation of ERK1/2 and P38 in IPEC-J2. Although TNF-α was upregulated, the downregulation of anti-inflammatory factor IL-10 (41) and increased cell viability denote that BTL-1 had an anti-inflammatory effect.

In general, the improvement of barrier function was correlated with the increase of TEER and the decrease of paracellular permeability (42, 43). The three proteins ZO-1, occludin, and claudin-1, are crucial for the TJ of the intestine to maintain intestinal permeability (44, 45). We found that BTL-1 can increase TEER and decrease paracellular permeability by upregulating tight junction protein. Furthermore, it shows that BTL-1 can enhance the intestinal barrier function *in vitro*.

To explore the role of BTL-1 in preventing IBD, we also used a DSS-induced IBD mouse model. We found that BTL-1 can effectively alleviate DSS-induced decreased food intake, increased disease activity index, immune organ index, anemia, and total white blood cell count. Further, we found that BTL-1 effectively inhibited the NF-κB and the MAPK signaling pathways, indicating that BTL-1 can downregulate the expression of pro-inflammatory factors (IL-6, IL-1β, and TNF-α). On the other hand, BTL-1 can promote the phosphorylation of P38 and ERK in IPEC-J2 cells, thus regulating the expression of TNF-α. However, BTL-1 has different activities in the IBD mouse model, which inhibits the phosphorylation of ERK and P38, thus downregulating the expression of TNF-α. Our results are consistent with the results of related literature in inhibiting the expression of TNF-α in mice (46).

In terms of protecting the intestinal barrier, BTL-1 significantly increased the expression of TJ proteins and reduced the permeability of the intestinal tract. In addition, the histological changes of the intestinal mucosa were close to those of the control group. This finding denotes that BTL-1 can sustain the integrity of the mouse intestinal barrier by increasing the expression of TJ proteins.

Herein, BTL-1 showed different activities on the MAPK signaling pathway in IPEC-J2 and mouse models. In IPEC, BTL-1 upregulates TNF-α by activating the phosphorylation of ERK1/2 and P38. Whereas, in mice, BTL-1 downregulates TNF-α by inhibiting the phosphorylation of ERK1/2 and P38. Due to different animal species and cell types, the same component often shows various activities. In this context, Gong et al. found that cortisol inhibition of oocyte maturation could not be relieved by NR3C1 inhibitor RU486 in mice, but RU486 overcame it in pigs (47). Further, Simon et al. found that *Ascaris suum* adult body fluid (ABF) did not affect cytokine production in IPEC-J2 cells; however, it suppressed LPS-induced secretion of IL-6 and TNF-α in PBMC (41). Therefore, it is reasonable that BTL-1 has different activities on the MAPK signaling pathway in IPEC and mice. However, we did not explore the mechanism of this difference that necessitated further experimental works.

## Conclusion

In conclusion, BTL-1 showed an anti-inflammatory effect in LPS-induced IPEC-J2 inflammation and DSS-induced IBD mouse models *via* TLR4/NF-κB and MAPK signaling pathways, resulting in the inhibition of pro-inflammatory cytokines expression. Meanwhile, by upregulating tight junction proteins, BTL-1 helps maintain the integrity of the intestinal barrier. These beneficial effects of BTL-1 in colitis may suggest the applicability of BTL-1 as a functional food or a new treatment strategy for human IBD. Identifying a novel function for BTL-1 may provide a basis for developing L-arabinose and new approaches to promote or protect gut health.

## Data Availability Statement

The original contributions presented in the study are included in the article/Supplementary Material, further inquiries can be directed to the corresponding author/s.

## Ethics Statement

The animal study was reviewed and approved by the protocol was approved by the Committee on the Ethics of Animal Experiments at Guangdong Ocean University (Permit No.: 201–1231).

## Author Contributions

SC: conceptualization, methodology, validation, and writing. XN, SM, JW, MB, TY, LW, CH, YZ, YY, and XL: resources and supervision. AA: review and editing. XJ: funding acquisition, project administration, and writing—review and editing. All authors contributed to the article and approved the submitted version.

## Funding

This study was supported by the National Natural Science Foundation of China [grant numbers: 31472243, 31902314]; Natural Science Foundation of Guangdong Province, China [grant numbers: 2019A1515011142, 2018A030307046]; the Project of Enhancing School with Innovation of Guangdong Ocean University [grant number: GDOU230419057] and the Program for Scientific Research Start-Fund of Guangdong Ocean University [grant number: 101402/R17088]; the Basic Research Project of Shenzhen Science and Technology Innovation Commission [JCYJ20190813105005619, JCYJ20190813142005766]; Shenzhen Dapeng New District Industrial Development Fund [No. KY20180203]; Shenzhen Dapeng New District Scientific and Technological Research and Development Fund [No. KJYF202001-07]; The Special Project in Key Fields of Guangdong Provincial Higher Education Institutions (Biomedicine and Health Care) [2021ZDZX2064]; and The Innovation and Development Project about Marine Economy Demonstration of Zhanjiang City [XM-202008-01B1].

## Conflict of Interest

The authors declare that the research was conducted in the absence of any commercial or financial relationships that could be construed as a potential conflict of interest.

## Publisher's Note

All claims expressed in this article are solely those of the authors and do not necessarily represent those of their affiliated organizations, or those of the publisher, the editors and the reviewers. Any product that may be evaluated in this article, or claim that may be made by its manufacturer, is not guaranteed or endorsed by the publisher.
