# Corrigendum: Coral-derived endophytic fungal product, butyrolactone-I, alleviates LPS induced intestinal epithelial cell inflammatory response through TLR4/NF-κB and MAPK signaling pathways: An *in vitro* and *in vivo* studies

**DOI:** 10.3389/fnut.2023.1191154

**Published:** 2023-04-04

**Authors:** Shengwei Chen, Yi Zhang, Xueting Niu, Sahar Ghulam Mohyuddin, Jiayin Wen, Minglong Bao, Tianyue Yu, Lianyun Wu, Canyin Hu, Yanhong Yong, Xiaoxi Liu, A. M. Abd El-Aty, Xianghong Ju

**Affiliations:** ^1^Department of Veterinary Medicine, Guangdong Ocean University, Zhanjiang, China; ^2^Shenzhen Institute of Guangdong Ocean University, Shenzhen, China; ^3^College of Food Science and Technology, Guangdong Ocean University, Zhanjiang, China; ^4^State Key Laboratory of Bio Based Material and Green Papermaking, College of Food Science and Engineering, Qilu University of Technology, Shandong Academy of Science, Jinan, China; ^5^Department of Pharmacology, Faculty of Veterinary Medicine, Cairo University, Giza, Egypt; ^6^Department of Medical Pharmacology, Faculty of Medicine, Atatürk University, Erzurum, Turkey

**Keywords:** butyrolactone-I, anti-inflammatory, intestinal barrier, IBD, TLR4/NF-κB, MAPK

In the published article, there was an error in the legend for Figure 3 as published. This sentence previously stated:

“The consequences of BTL-1 on the expression of cytokines were determined by E 50 μM) for 24 h and then treated with LPS (50 μm/mL) for another 1 h.”

The corrected legend appears below.

“Impact of BTL-1 on inflammatory cytokines production in LPS-induced IPEC-J2 cell. The outcome of BTL-1 on IPEC-J2 cell viability **(A,B)**. The consequences of BTL-1 on the expression of cytokines were determined by ELISA **(C–F)** and qPCR **(G–J)**. The consequences of BTL-1 on the expression of cytokines were determined by ELISA. The cells were treated with BTL-1 (10, 20, and 50 μM) for 24 h and then treated with LPS (50 μg/mL) for another 1 h. The data shown are representative of three independent experiments. The results were expressed as the means ± SEM. ^#^*P* < 0.05, ^##^*P* < 0.01, compared to non-treated group, ^*^P < 0.05, ^**^P < 0.01 compared to LPS group.”

In the published article, there was an error in Table 1 as published. Since our qPCR primers are designed by Shanghai Sangong Biotechnology Co., Ltd., there were a large number of primer sequences in the same account, which led to our submission error. At the same time, we also found that the primer of β-actin gene was missing. The corrected Table 1 and its caption appear below.

**Table 1:** Primer sequence.

**Gene ID**	**Primer**	**Sequence (5'-3')**
414396	pig β-actin Forward	GCTGTCCCTGTATGCCTCT
	pig β-actin Reverse	GATGTCACGCACGATTTCC
399500	pig IL-6 Forward	ATAAGGGAAATGTCGAGGCTGTGC
	pig IL-6 Reverse	GGGTGGTGGCTTTGTCTGGATTC
397122	pig IL-1β Forward	CAAGCCAGAGAAGCAAGGTGTCC
	pig IL-1β Reverse	GCCGTCCTCAGCAGCAAGAAG
397106	pig IL-10 Forward	AGCCAGCATTAAGTCTGAGAACAGC
	pig IL-10 Reverse	GGTCAGCAACAAGTCGCCCATC
397086	pig TNF-α Forward	AAAGGACACCATGAGCACGGAAAG
	pig TNF-α Reverse	CGCCACGAGCAGGAATGAGAAG
399541	pig TLR4 Forward	GCCATCGCTGCTAACATCATC
	pig TLR4 Reverse	CTCATACTCAAAGATACACCATCGG
100135665	pig P65 Forward	CTGAGGCTATAACTCGCTTGGTGAC
	pig P65 Reverse	CATGTCCGCAATGGAGGAGAAGTC
100157586	pig IκB Forward	GGCAGTGACATGAGTGGCAGATAC
	pig IκB Reverse	CTTCCTTGAGGCTGTGCTTCTTCC
445013	pig ERK1 Forward	ACGTCATTGGCATCCGAGACATTC
	pig ERK1 Reverse	GAGGAAGTAGCAGATGTGGTCGTTG
100626611	pig P38 Forward	CGCCTGTGAAGACCTCCTTGAAC
	pig P38 Reverse	TTCCTGTCCTCCACCTTCCGAAG
396610	pig JNK Forward	ACTACAGAGCACCTGAGGTCATCC
	pig JNK Reverse	ATTTCTCCCATAATGCACCCCACAG
397236	pig Occludin Forward	CAGTGGTAACTTGGAGGCGTCTTC
	pig Occludin Reverse	CGTCGTGTAGTCTGTCTCGTAATGG
100736682	pig ZO-1 Forward	CCAGGGAGAGAAGTGCCAGTAGG
	pig ZO-1 Reverse	TTTGGTGGGTTTGGTGGGTTGAC
100625166	pig Claudin1 Forward	AGAAGATGCGGATGGCTGTCATTG
	pig Claudin1 Reverse	ACCATACCATGCTGTGGCAACTAAG
11461	mouse β-actin Forward	GTGACGTTGACATCCGTAAAGA
	mouse β-actin Reverse	GCCGGACTCATCGTACTCC
16193	mouse IL-6 Forward	CTCCCAACAGACCTGTCTATAC
	mouse IL-6 Reverse	CCATTGCACAACTCTTTTCTCA
16176	mouse IL-1β Forward	GAAATGCCACCTTTTGACAGTG
	mouse IL-1β Reverse	TGGATGCTCATCAGGACAG
16153	mouse IL-10 Forward	TTCTTTCAAACAAAGGACCAGC
	mouse IL-10 Reverse	GCAACCCAAGTAACCCTTAAAG
21926	mouse TNF-α Forward	ATGTCTCAGCCTCTTCTCATTC
	mouse TNF-α Reverse	GCTTGTCACTCGAATTTTGAGA
21898	mouse TLR4 Forward	GCCATCATTATGAGTGCCAATT
	mouse TLR4 Reverse	AGGGATAAGAACGCTGAGAATT
19697	mouse P65 Forward	TGCGATTCCGCTATAAATGCG
	mouse P65 Reverse	ACAAGTTCATGTGGATGAGGC
12675	mouse IκB Forward	TTGGGTTATGCCAAAGATGTTG
	mouse IκB Reverse	GCTGTGTACGGCTTATTTTCAA
26417	mouse ERK1 Forward	CAGCTCAACCACATTCTAGGTA
	mouse ERK1 Reverse	TCAAGAGCTTTGGAGTCAGATT
26416	mouse P38 Forward	AGGAATTCAATGACGTGTACCT
	mouse P38 Reverse	AGGTCCCTGTGAATTATGTCAG
26419	mouse JNK1 Forward	TTGAAAACAGGCCTAAATACGC
	mouse JNK1 Reverse	GTTTGTTATGCTCTGAGTCAGC
18260	mouse Occludin Forward	TGCTTCATCGCTTCCTTAGTAA
	mouse Occludin Reverse	GGGTTCACTCCCATTATGTACA
21872	mouse ZO-1 Forward	CTGGTGAAGTCTCGGAAAAATG
	mouse ZO-1 Reverse	CATCTCTTGCTGCCAAACTATC
12737	mouse Claudin1 Forward	AGATACAGTGCAAAGTCTTCGA
	mouse Claudin1 Reverse	CAGGATGCCAATTACCATCAAG

In the published article, there was an error in Figure 10 as published. Due to the poor shooting effect of some tissue sections, we adjusted the angle of the sections, and the remarks were not clear enough, resulting in mistakenly uploading two images of the same tissue section, namely, the HE staining sections of the low dose group and the medium dose group. The corrected Figure 10 and its caption appear below.

**Figure 10:**
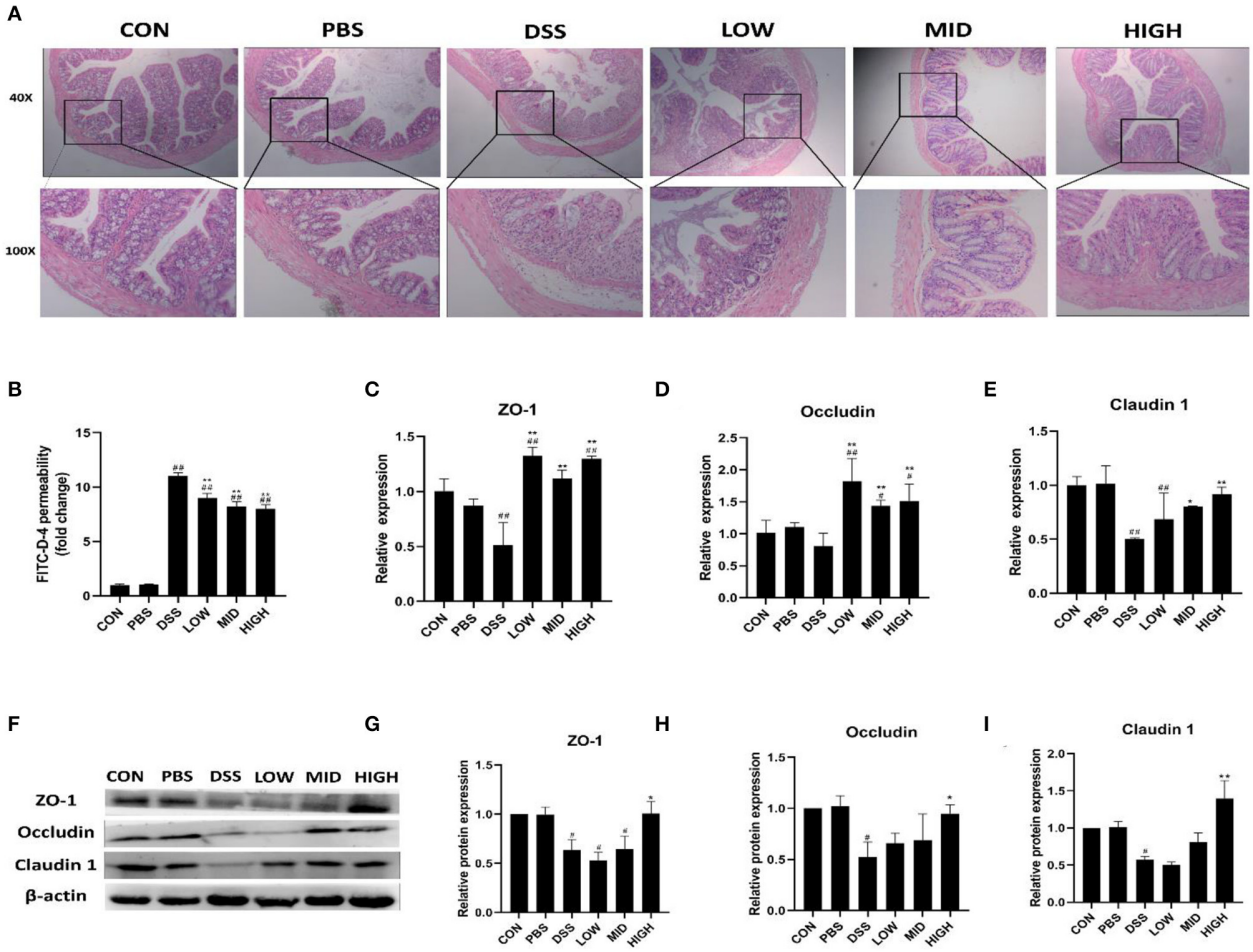
The effect of BTL-1 on the intestinal injury of IBD mouse model induced by DSS. Histopathological changes after DSS stimulation in the colon **(A)**. The *in vitro* intestinal barrier function evaluated by FITC-D fluorescence intensity **(B)**. Upregulation of BTL-1 on tight junction proteins as determined by qPCR **(C–E)** and western blotting **(F–I)**. The results were expressed as the means ± SEM of *n* = 3. ^#^*P* < 0.05, ^##^*P* < 0.01, compared to control group, ^*^P < 0.05, ^**^P < 0.01 compared to DSS group.

In the published article, there was an error in **Materials and Methods**, Subsection “*Induction of Experimental Colitis*.” This sentence previously stated:

“The animals were randomly allocated to one of the following five groups (*n* = 5): normal control (only given food and water without DSS), DSS model (administered DSS in drinking water), low-concentration BTL-1 (administered 10 mg/kg DSS, p.o), middle-concentration BTL-1 (taken 20 mg/kg DSS, p.o), and high-concentration BTL-1 group (given 50 mg/kg DSS, p.o) group.”

The corrected sentence appears below:

“The animals were randomly allocated to one of the following five groups (*n* = 5): normal control (only given food and water without DSS), DSS model (administered DSS in drinking water), low-concentration BTL-1 (administered 1 mg/kg BTL-1, p.o), middle-concentration BTL-1 (taken 2 mg/kg BTL-1, p.o), and high-concentration BTL-1 group (given 5 mg/kg BTL-1, p.o) group.”

The authors apologize for these errors and state that this does not change the scientific conclusions of the article in any way. The original article has been updated.

