# Supplementary material for: Coral-Derived Endophytic Fungal Product, Butyrolactone-I, Alleviates Lps Induced Intestinal Epithelial Cell Inflammatory Response Through TLR4/NF-κB and MAPK Signaling Pathways: An in vitro and in vivo Studies
Source: Front Nutr. 2021 Oct 1;8:748118. doi: 10.3389/fnut.2021.748118 (PMC8517189; doi:10.3389/fnut.2021.748118)
Supplement: Supplementary file 2 [file Data_Sheet_1.ZIP › Docking(Butyrolactone ó±-TLR4)/╫≈═╝/Butyrolactone ó±-TLR4 ╢╘╜╙╜ß╣√▒¿╕μ.docx]

1. 分子对接步骤

我们采用ChemBioDraw Ultra 14.0画出化合物Butyrolactone Ⅰ的结构（图1），然后用ChemBio3D Ultra 14.0转化为三维结构，并使用MMFF94力场进行优化。Toll样受体（TLR4）的三维结构（PDB ID: 2Z64）从RCSB Protein Data Bank (www.rcsb.org)下载得到。TLR4和化合物Butyrolactone Ⅰ均使用AutodockTools 1.5.6[1-2]转化为PDBQT格式。本课题采用Autodock vina 1.1.2[3]进行分子对接研究。TLR4活性位点的坐标为：center_x = -28.441，center_y = -17.705，center_z = -22.582；size_x = 20，size_y = 20，size_z = 20。为了增加计算的准确度，我们将参数exhaustiveness设置为100。除了特别说明，其他参数均采用默认值。最后，选取打分值最高的构象用PyMoL 1.7.6进行结果分析。


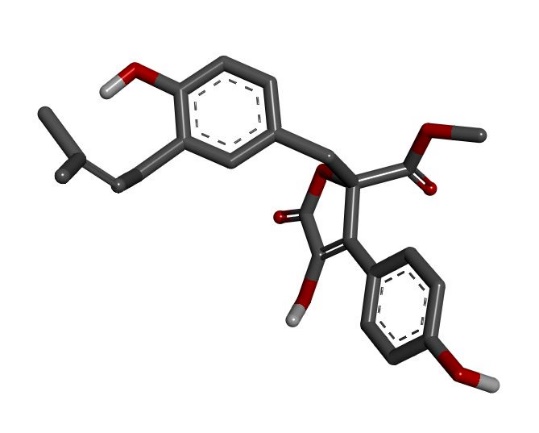


图1. Butyrolactone Ⅰ的结构

2. 分子对接结果分析

为了从分子水平阐明TLR4与化合物Butyrolactone Ⅰ的作用模式，我们将化合物Butyrolactone Ⅰ对接至TLR4的活性口袋，其亲和力为-8.8 kcal mol^−1^，理论结合模式如图2和图3所示。由图2可以看出，化合物Butyrolactone Ⅰ的活性口袋中呈现出紧凑的结合模式。由图3并结合2D图可知，Butyrolactone Ⅰ处于一个由氨基酸Ile-32、Ile-46、Ser-47、Ile-52、Val-61、Leu-78、Phe-121、Cys-133、Phe-151和Ile-153所组成的腔袋，形成强烈的疏水相互作用。重要的是，Butyrolactone Ⅰ的1个羟基可以与氨基酸Ser-47形成长为3.6 Å的氢键作用（图3），且与氨基酸Phe-151形成π-π键，这些是Butyrolactone Ⅰ和TLR4之间最主要的作用力。所有的这些相互作用使得Butyrolactone Ⅰ和TLR4形成稳定的复合物。

总之，上述的分子对接研究对Butyrolactone Ⅰ和TLR4的相互作用给予了合理的解释。


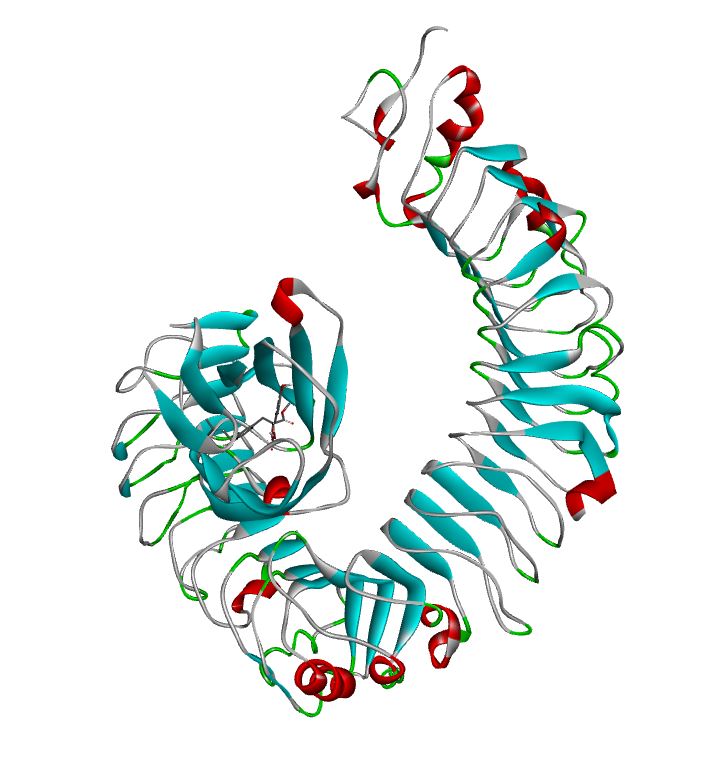


图2. Butyrolactone Ⅰ对接至TLR4的活性口袋（整体图）


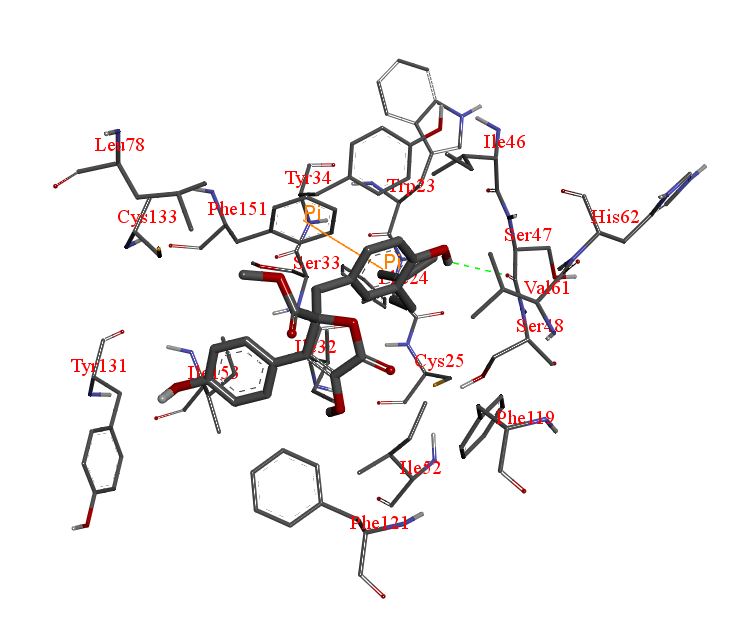


图3. Butyrolactone Ⅰ对接至TLR4的活性口袋（细节图）

参考文献：

1. Sanner, M. F., Python: a programming language for software integration and development. J Mol Graph Model 1999, 17 (1), 57-61.

2. Morris, G. M.; Huey, R.; Lindstrom, W.; Sanner, M. F.; Belew, R. K.; Goodsell, D. S.; Olson, A. J., AutoDock4 and AutoDockTools4: Automated docking with selective receptor flexibility. J Comput Chem 2009, 30 (16), 2785-91.

3. Trott, O.; Olson, A. J., AutoDock Vina: improving the speed and accuracy of docking with a new scoring function, efficient optimization, and multithreading. J Comput Chem 2010, 31 (2), 455-61.
