# Supplementary figures and images for: Coral-Derived Endophytic Fungal Product, Butyrolactone-I, Alleviates Lps Induced Intestinal Epithelial Cell Inflammatory Response Through TLR4/NF-κB and MAPK Signaling Pathways: An in vitro and in vivo Studies
Source: Front Nutr. 2021 Oct 1;8:748118. doi: 10.3389/fnut.2021.748118 (PMC8517189; doi:10.3389/fnut.2021.748118)

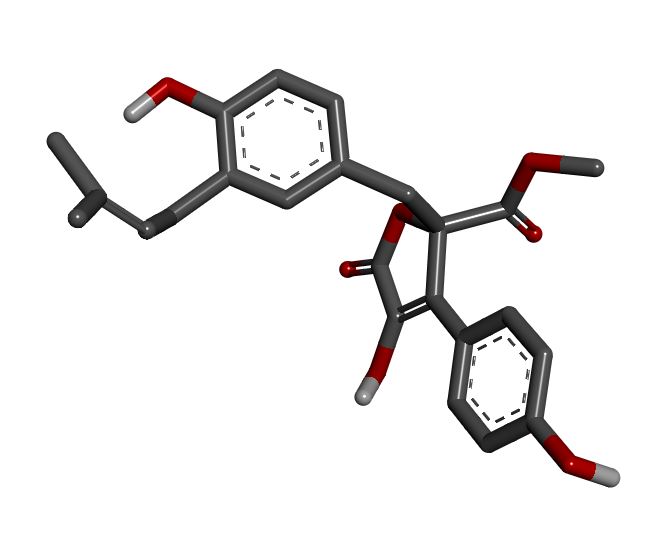

Supplement: Supplementary file 2 [file Data_Sheet_1.ZIP › Docking(Butyrolactone ó±-TLR4)/╫≈═╝/Butyrolactone ó±.JPG]

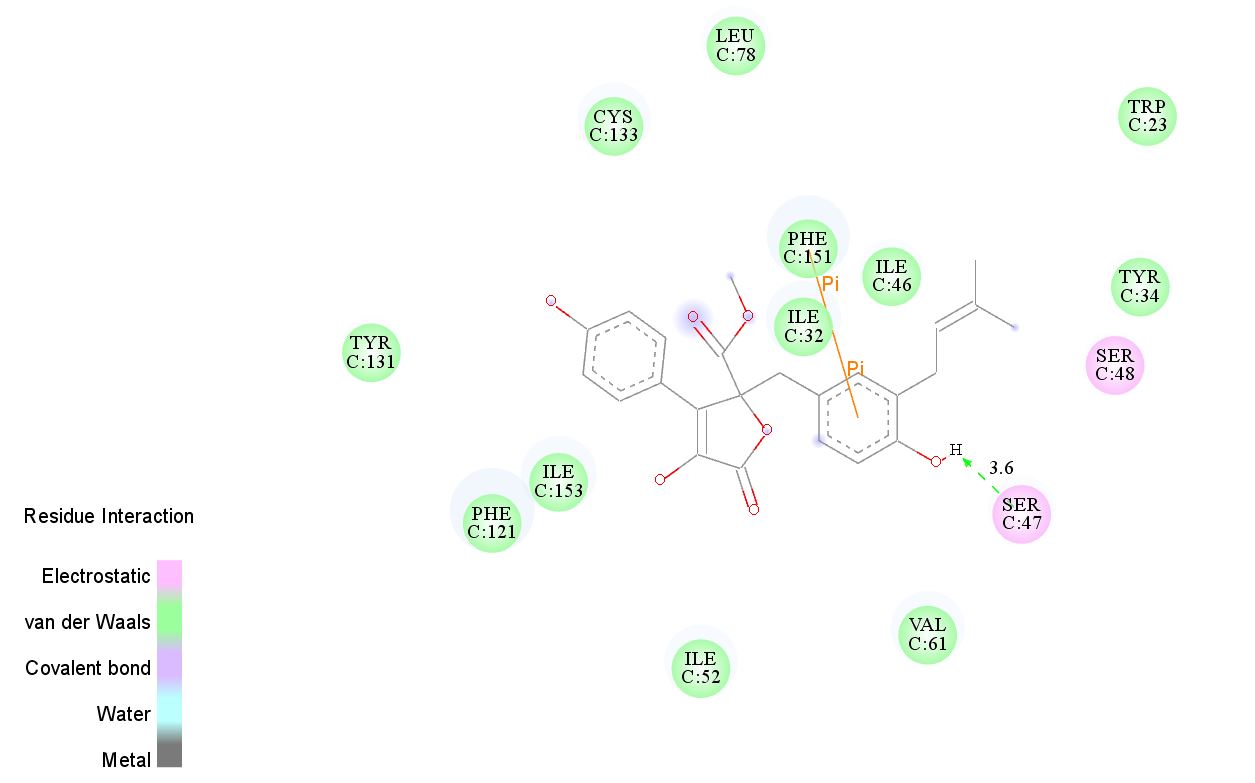

Supplement: Supplementary file 2 [file Data_Sheet_1.ZIP › Docking(Butyrolactone ó±-TLR4)/╫≈═╝/╢╘╜╙╜ß╣√-2D.JPG]

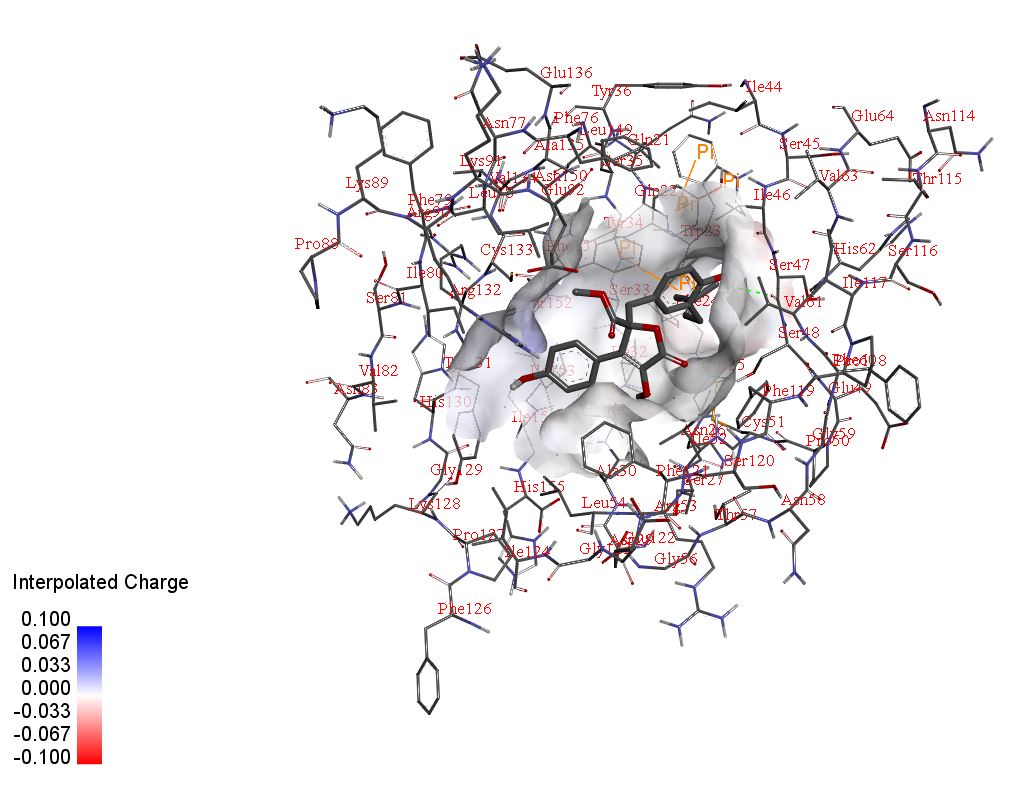

Supplement: Supplementary file 2 [file Data_Sheet_1.ZIP › Docking(Butyrolactone ó±-TLR4)/╫≈═╝/╢╘╜╙╜ß╣√-╛▓╡τ╧α╗Ñ╫≈╙├.JPG]

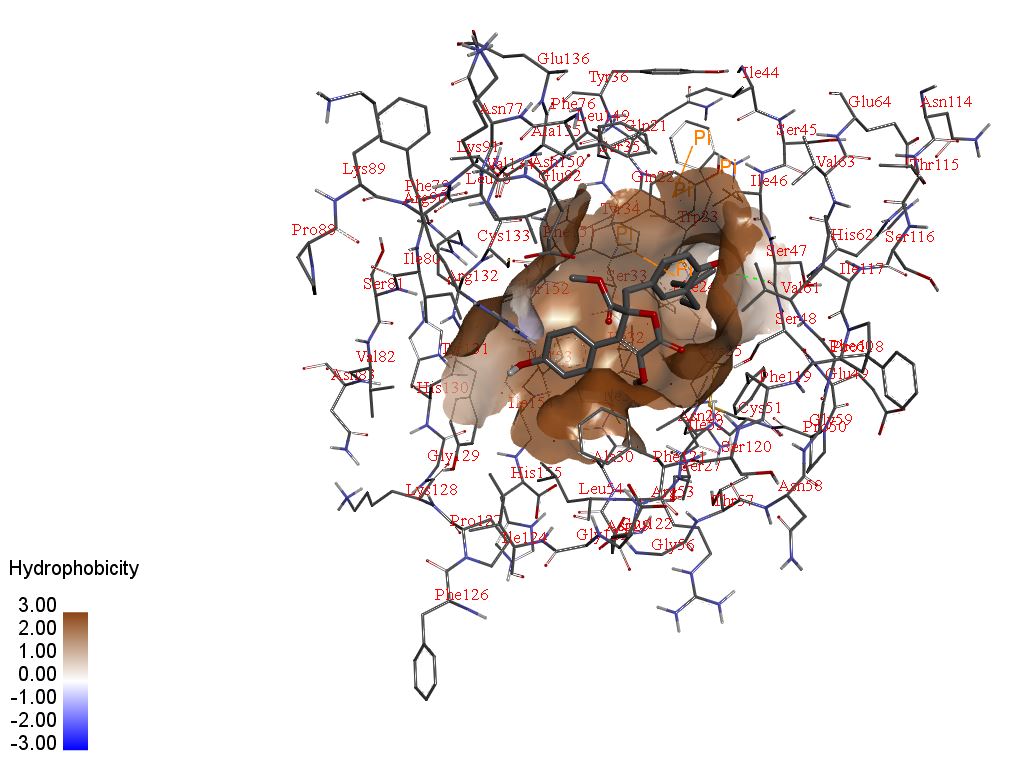

Supplement: Supplementary file 2 [file Data_Sheet_1.ZIP › Docking(Butyrolactone ó±-TLR4)/╫≈═╝/╢╘╜╙╜ß╣√-╟╫╦«╨╘.JPG]

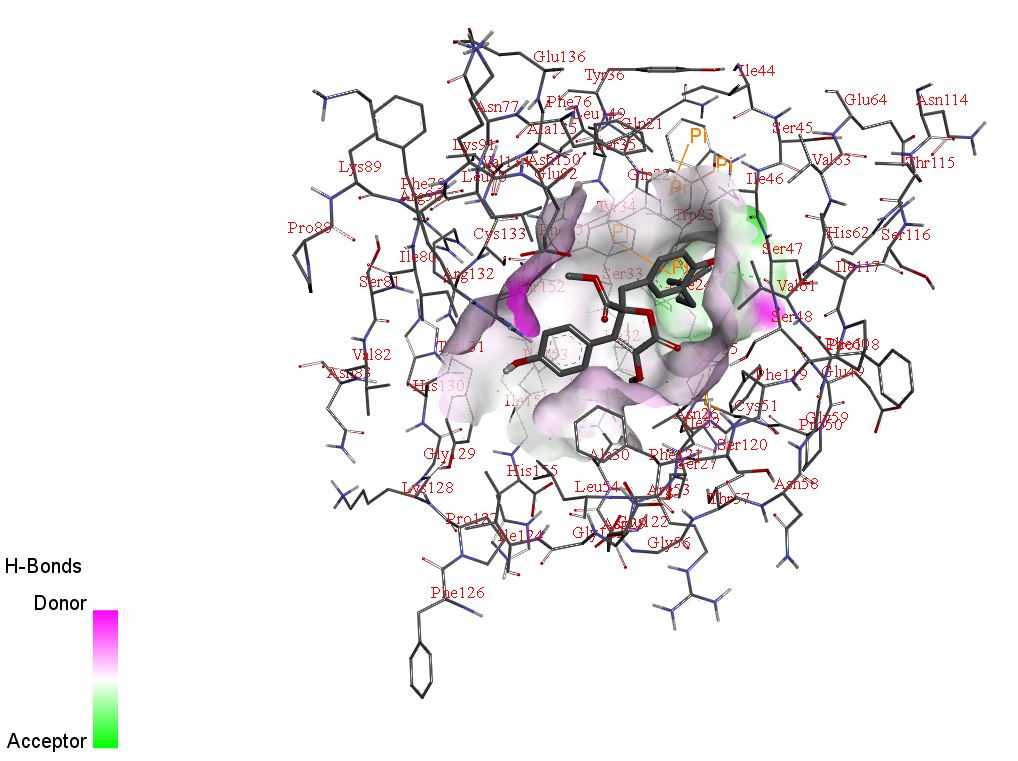

Supplement: Supplementary file 2 [file Data_Sheet_1.ZIP › Docking(Butyrolactone ó±-TLR4)/╫≈═╝/╢╘╜╙╜ß╣√-╟Γ╝n.JPG]

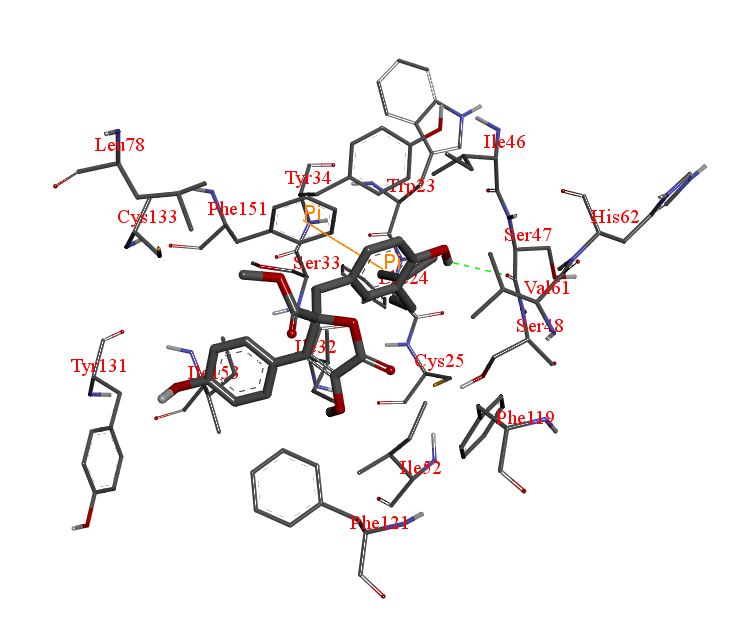

Supplement: Supplementary file 2 [file Data_Sheet_1.ZIP › Docking(Butyrolactone ó±-TLR4)/╫≈═╝/╢╘╜╙╜ß╣√-╧α╗Ñ╫≈╙├▓╨╗∙.JPG]

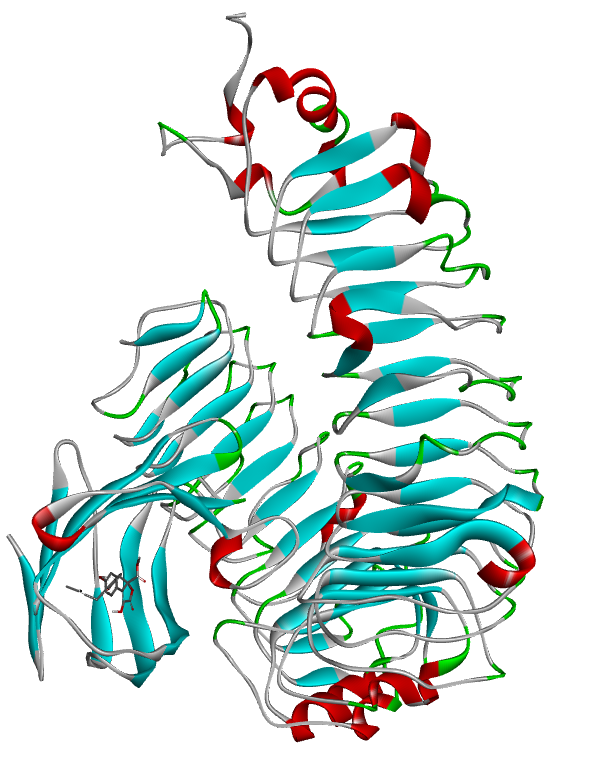

Supplement: Supplementary file 2 [file Data_Sheet_1.ZIP › Docking(Butyrolactone ó±-TLR4)/╫≈═╝/╢╘╜╙╜ß╣√-╒√╠σ.JPG]

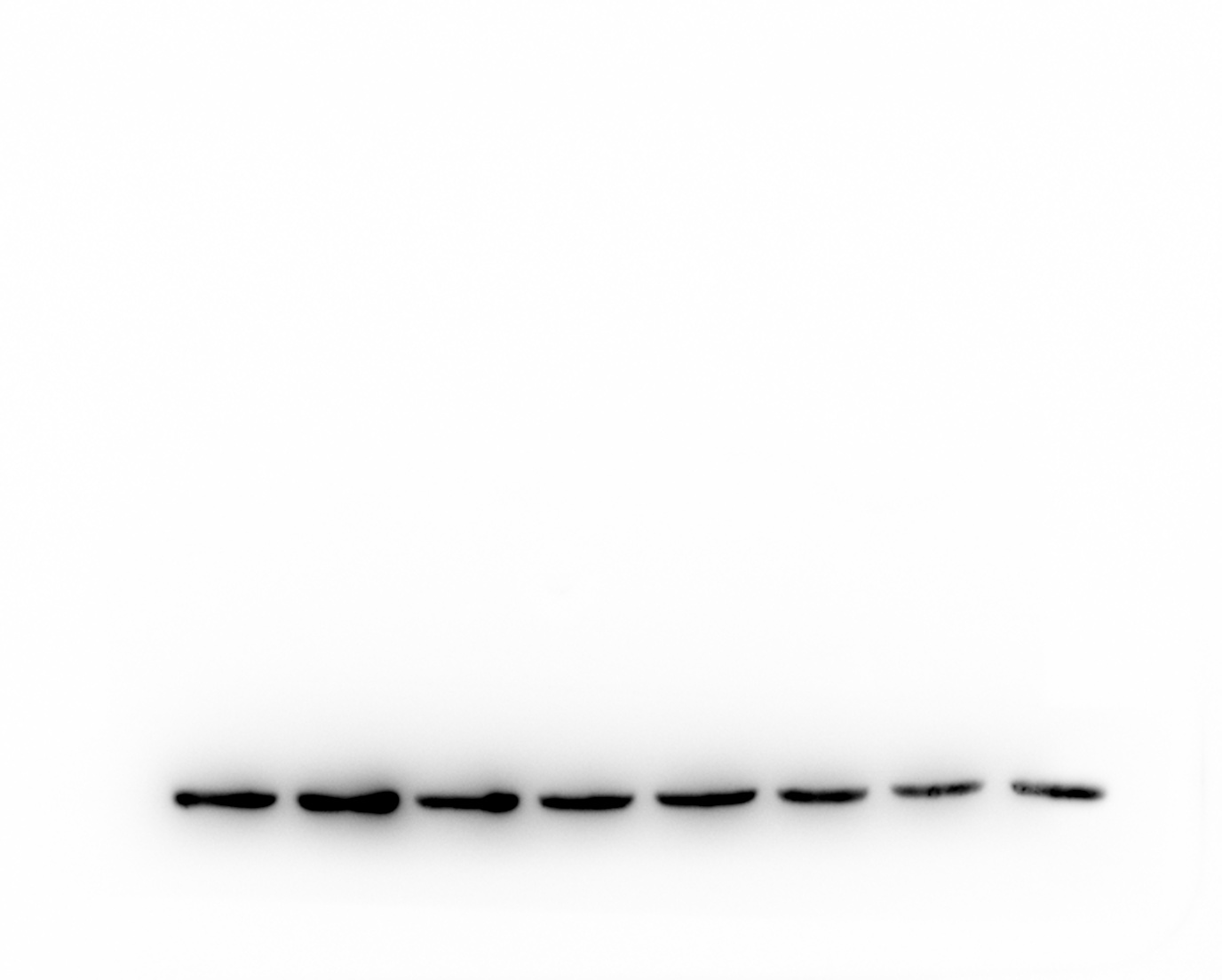

Supplement: Supplementary file 3 [file Data_Sheet_2.ZIP › fig5-wb/actin-3.tif]

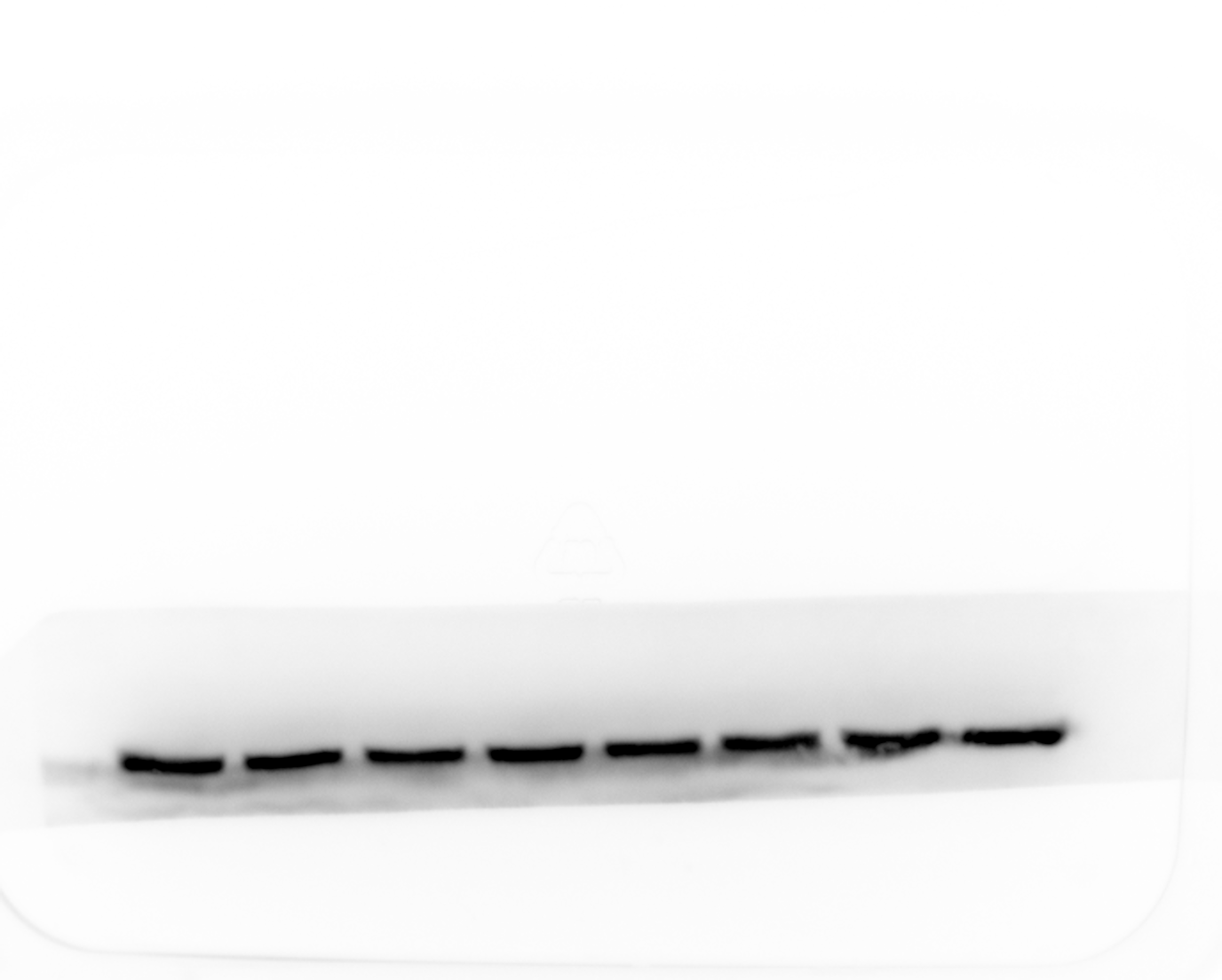

Supplement: Supplementary file 3 [file Data_Sheet_2.ZIP › fig5-wb/actin.tif]

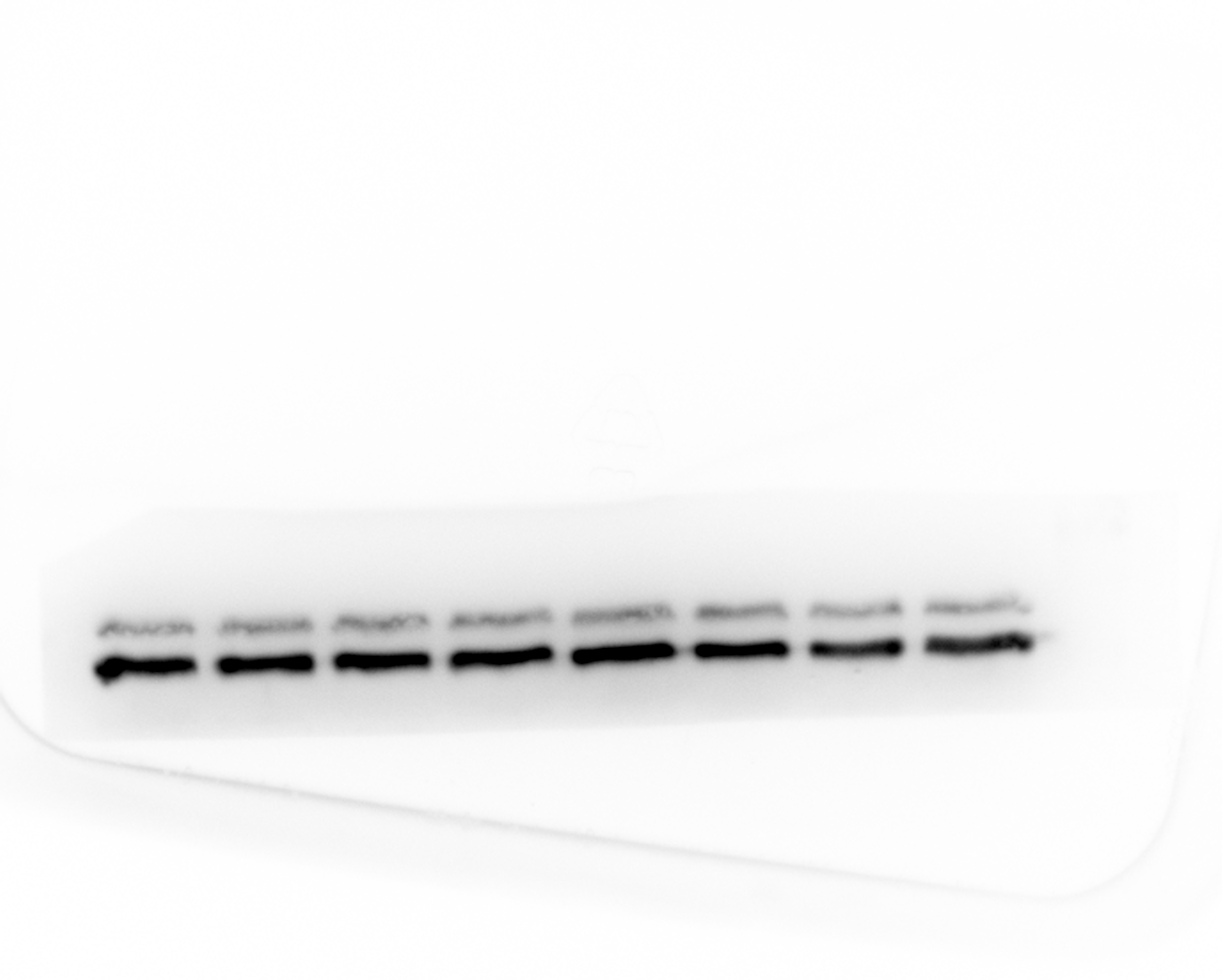

Supplement: Supplementary file 3 [file Data_Sheet_2.ZIP › fig5-wb/erk-2 (2).tif]

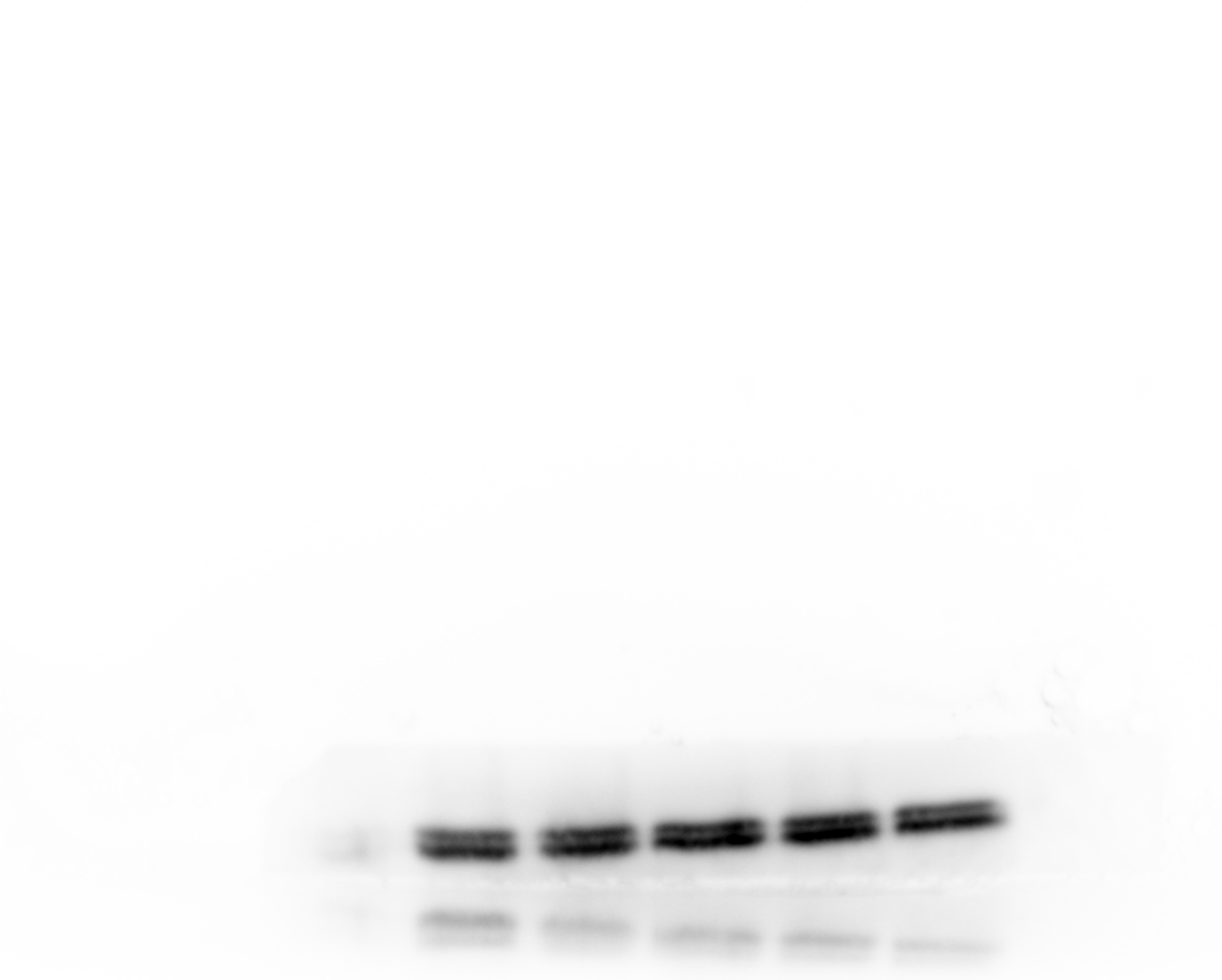

Supplement: Supplementary file 3 [file Data_Sheet_2.ZIP › fig5-wb/erk-2.tif]

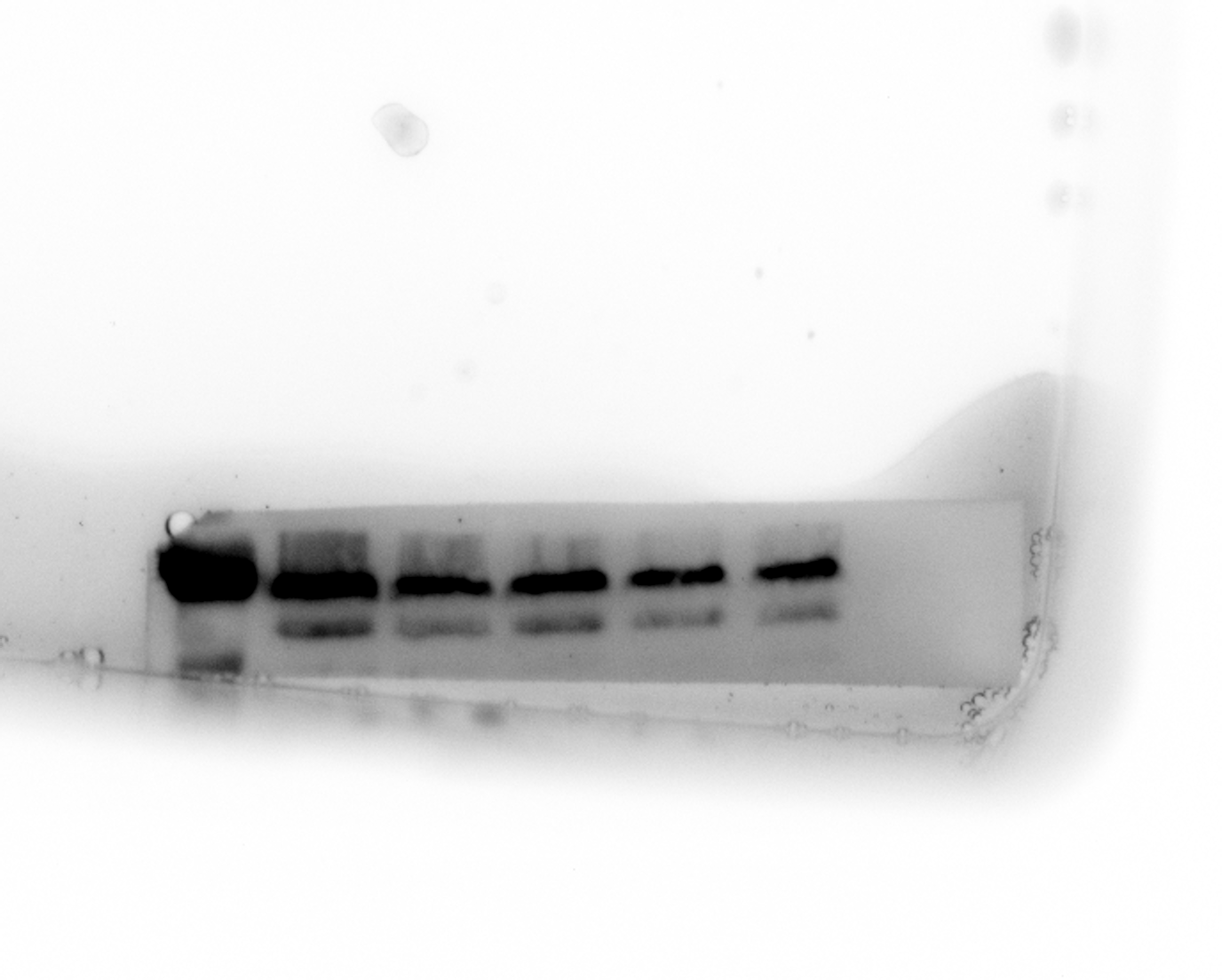

Supplement: Supplementary file 3 [file Data_Sheet_2.ZIP › fig5-wb/jnk-4.tif]

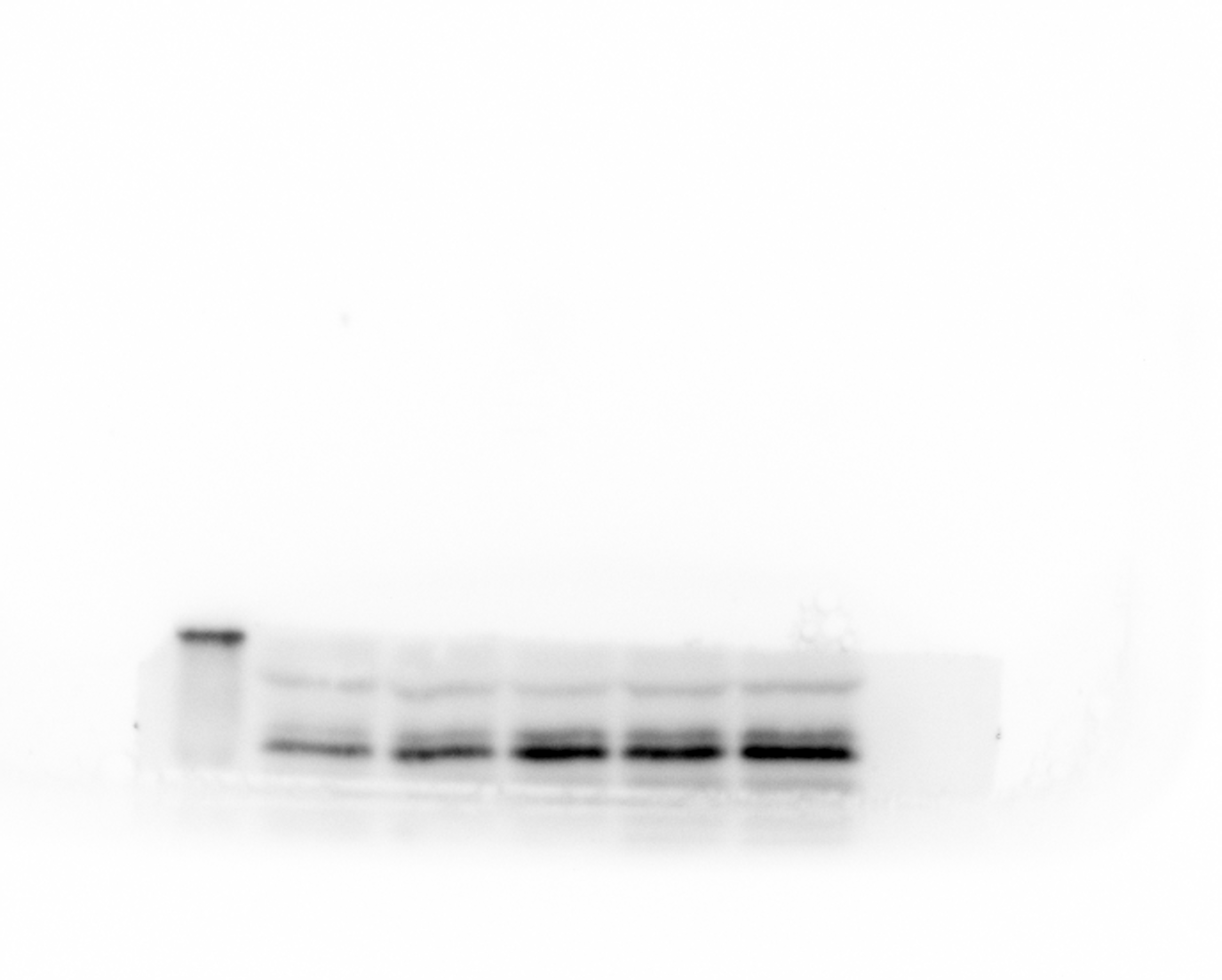

Supplement: Supplementary file 3 [file Data_Sheet_2.ZIP › fig5-wb/p-erk-2.tif]

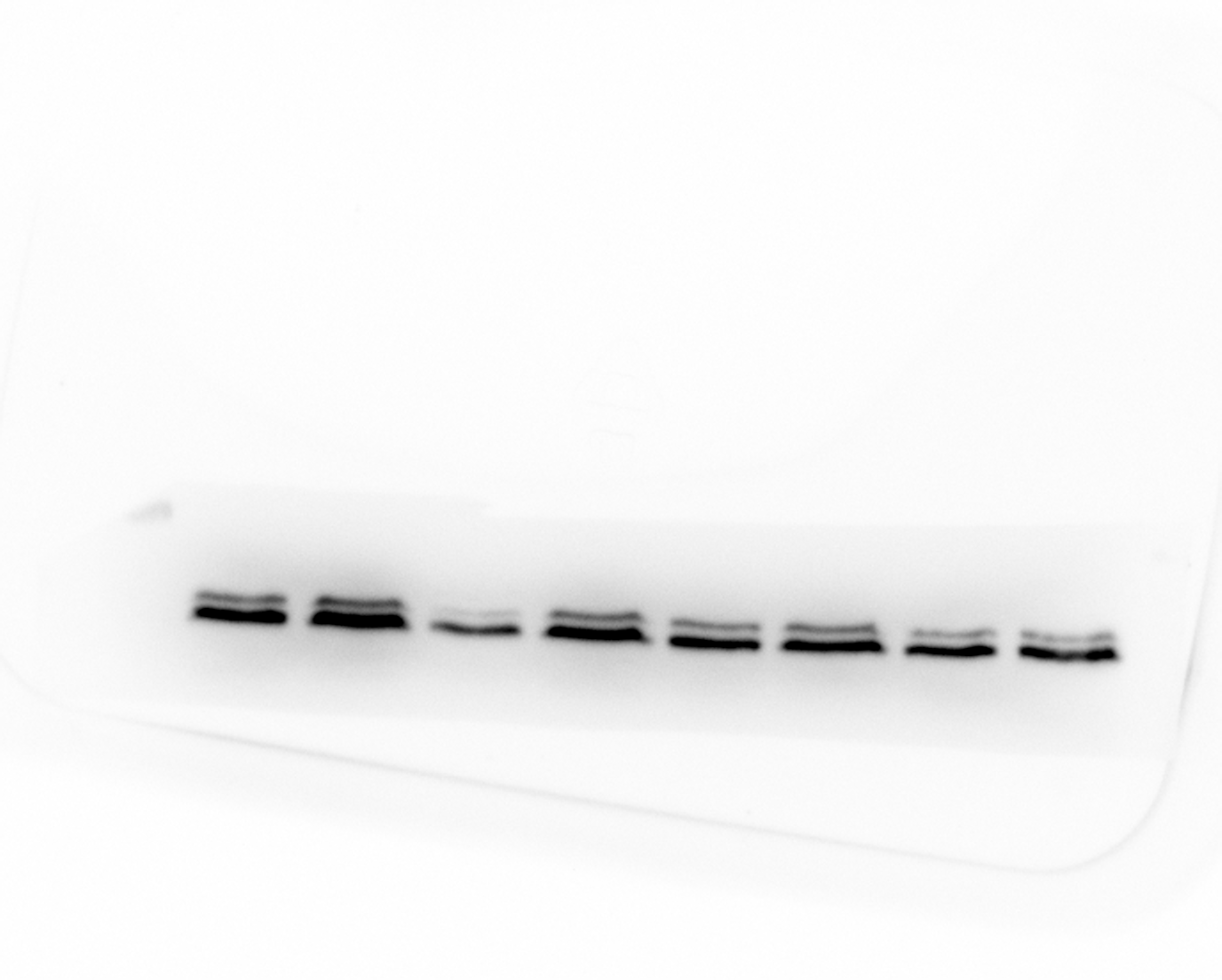

Supplement: Supplementary file 3 [file Data_Sheet_2.ZIP › fig5-wb/p-erk-3.tif]

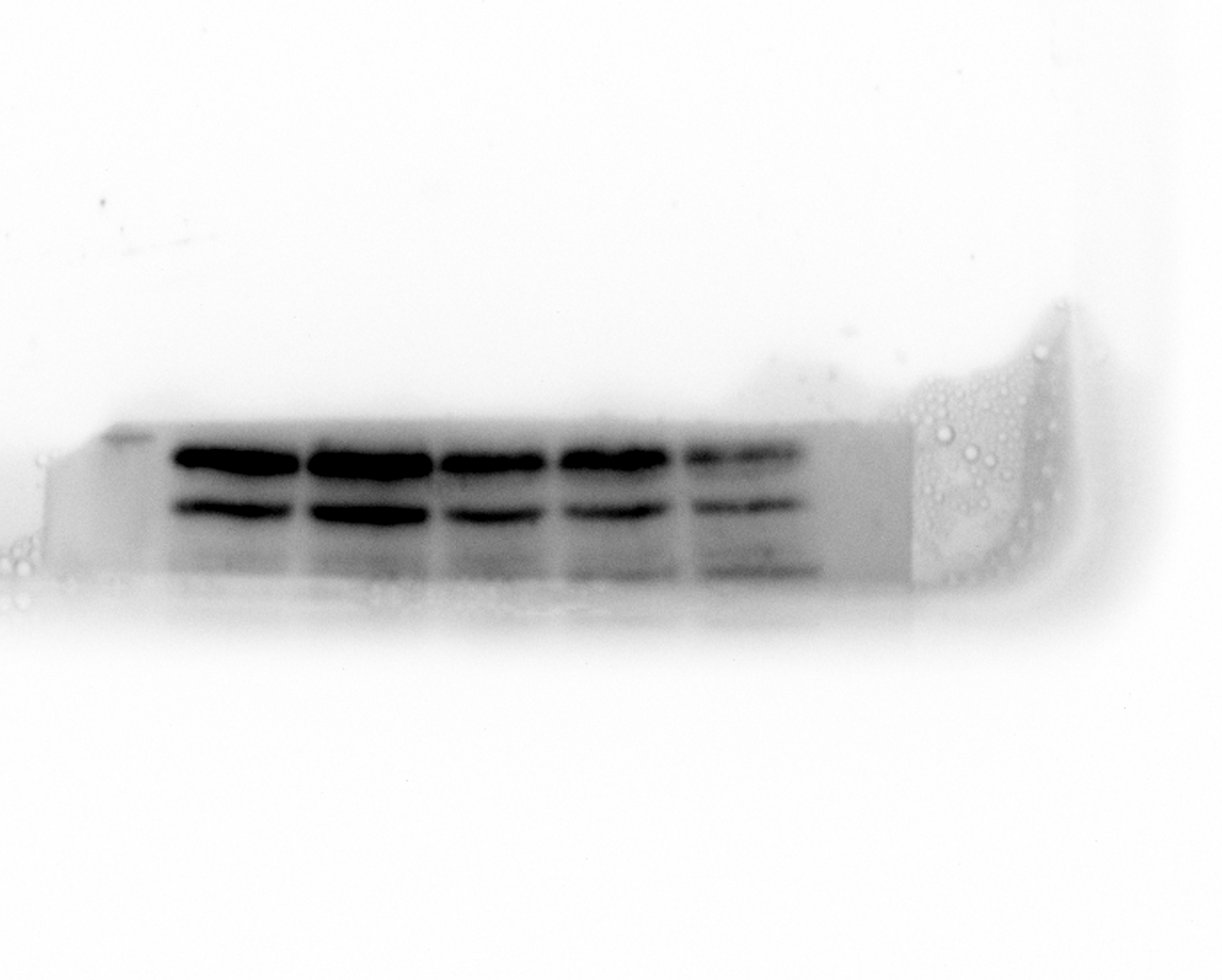

Supplement: Supplementary file 3 [file Data_Sheet_2.ZIP › fig5-wb/p-jnk.tif]

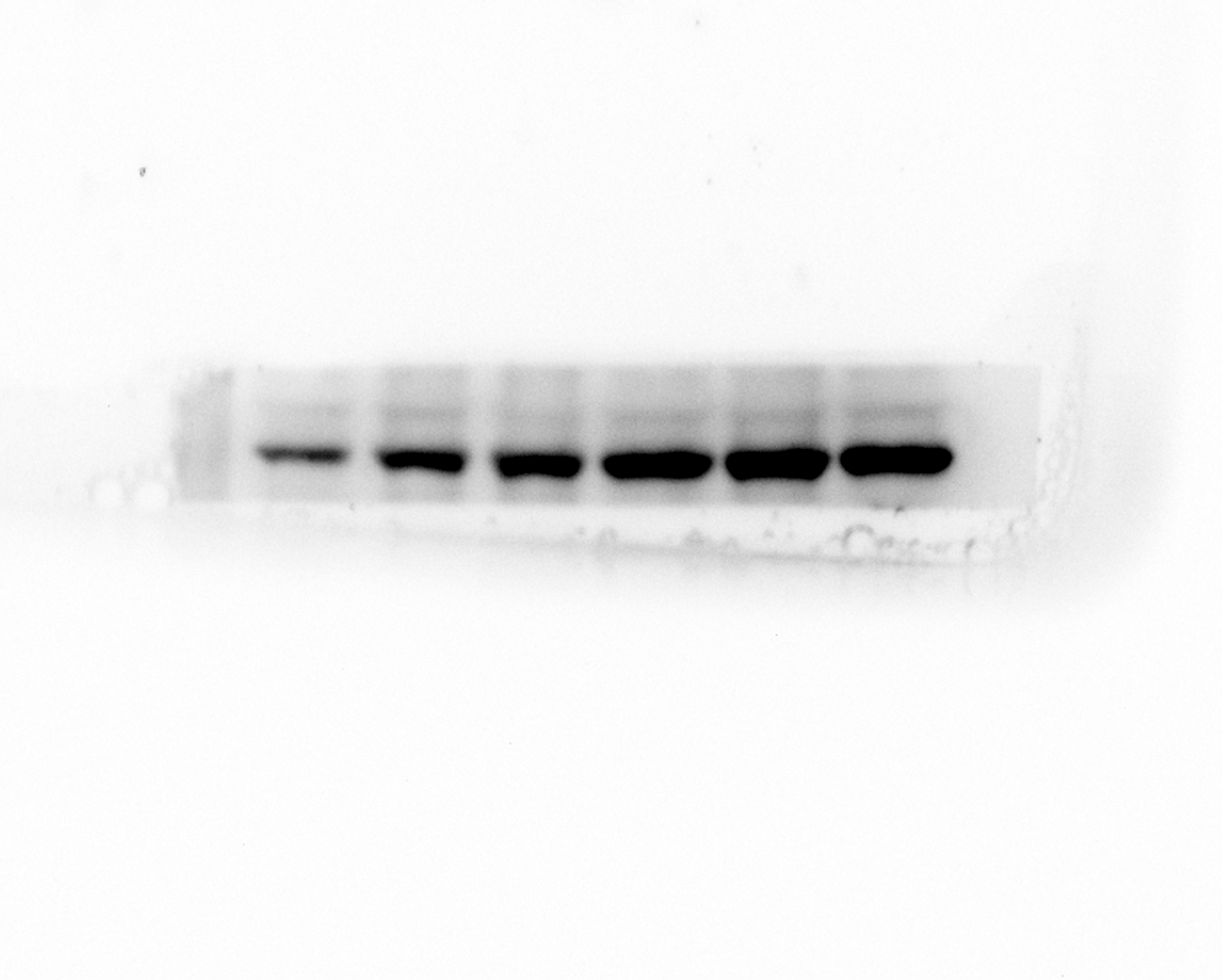

Supplement: Supplementary file 3 [file Data_Sheet_2.ZIP › fig5-wb/p-p38-2.tif]

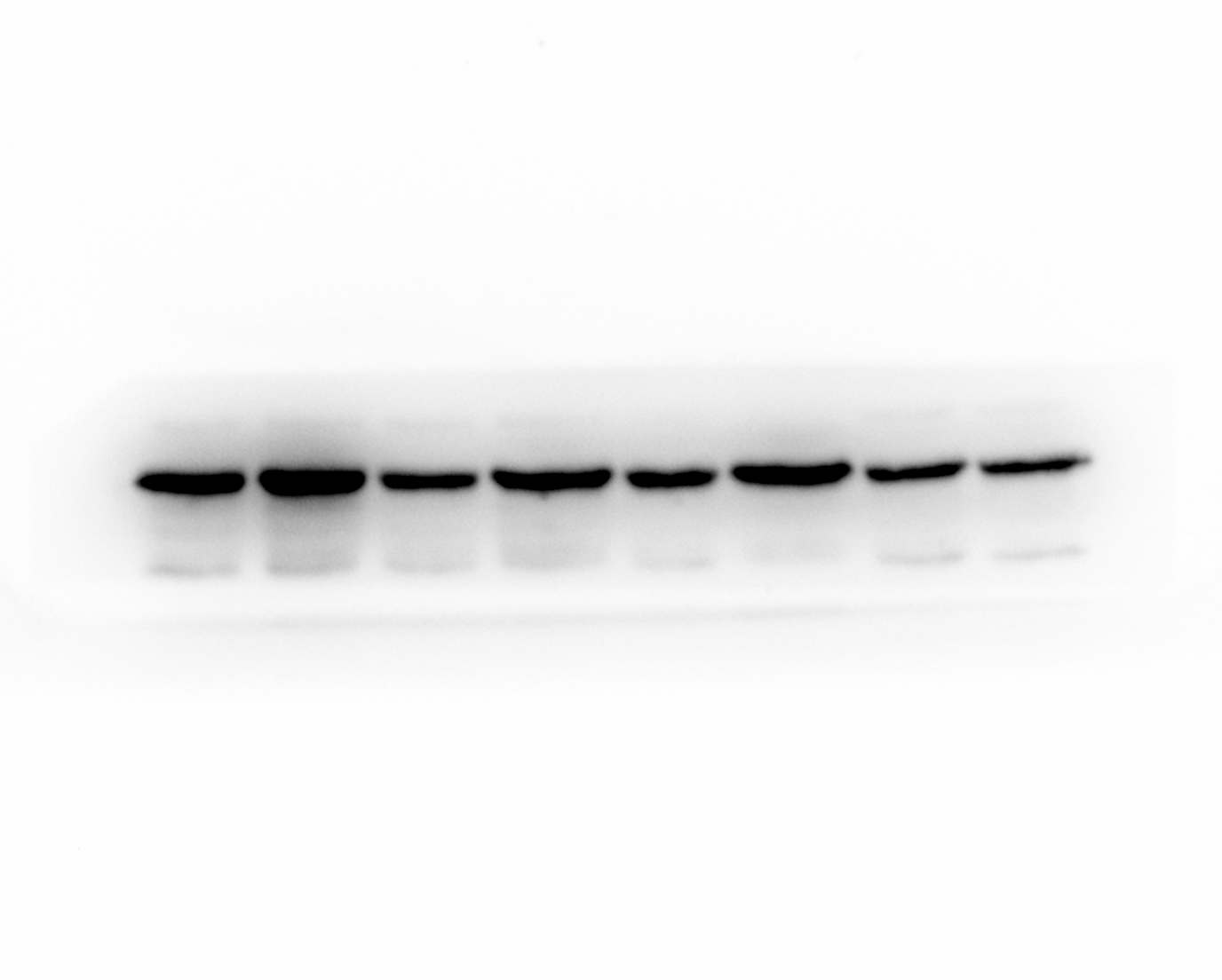

Supplement: Supplementary file 3 [file Data_Sheet_2.ZIP › fig5-wb/p-p38-3.tif]

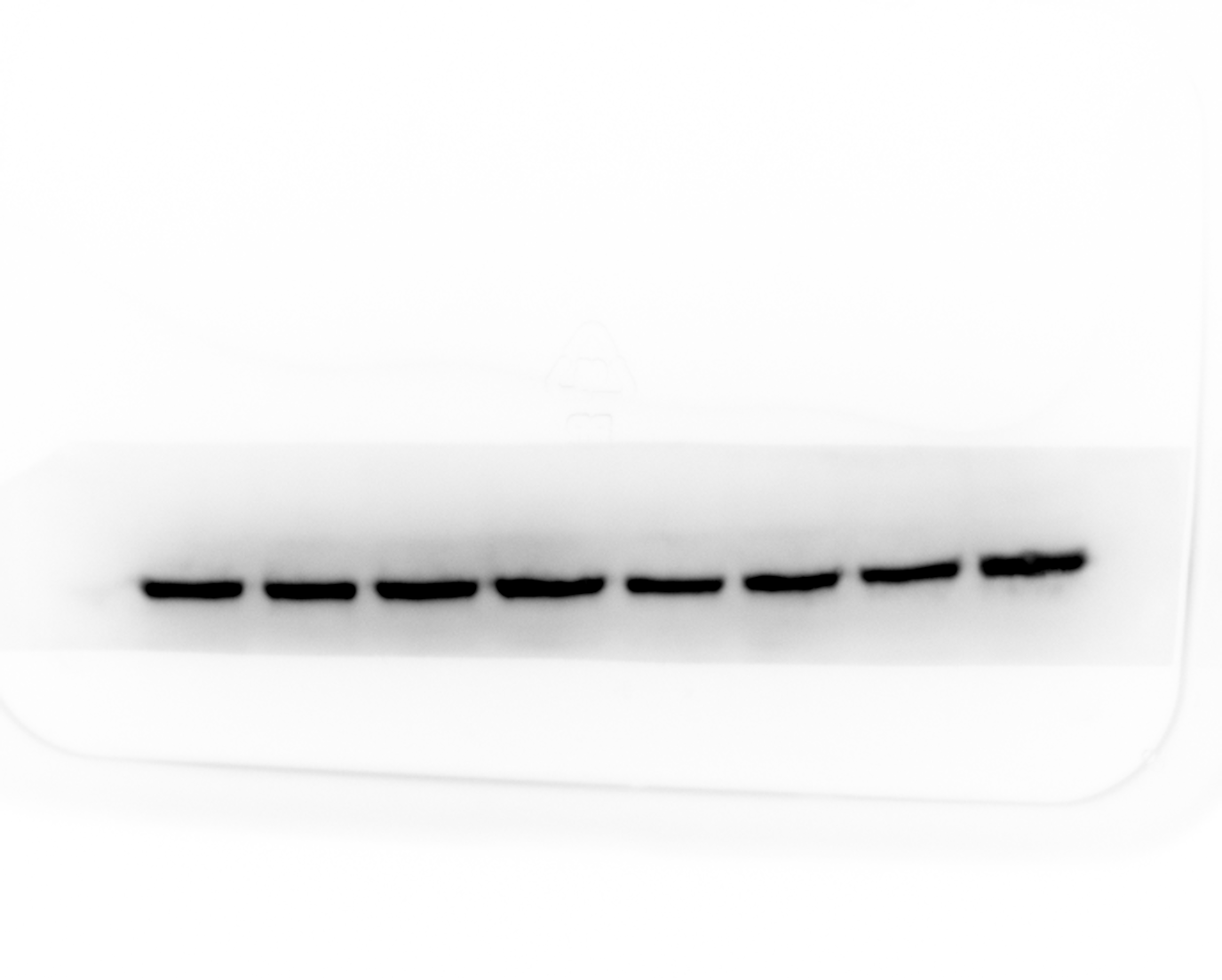

Supplement: Supplementary file 3 [file Data_Sheet_2.ZIP › fig5-wb/p38-5.tif]

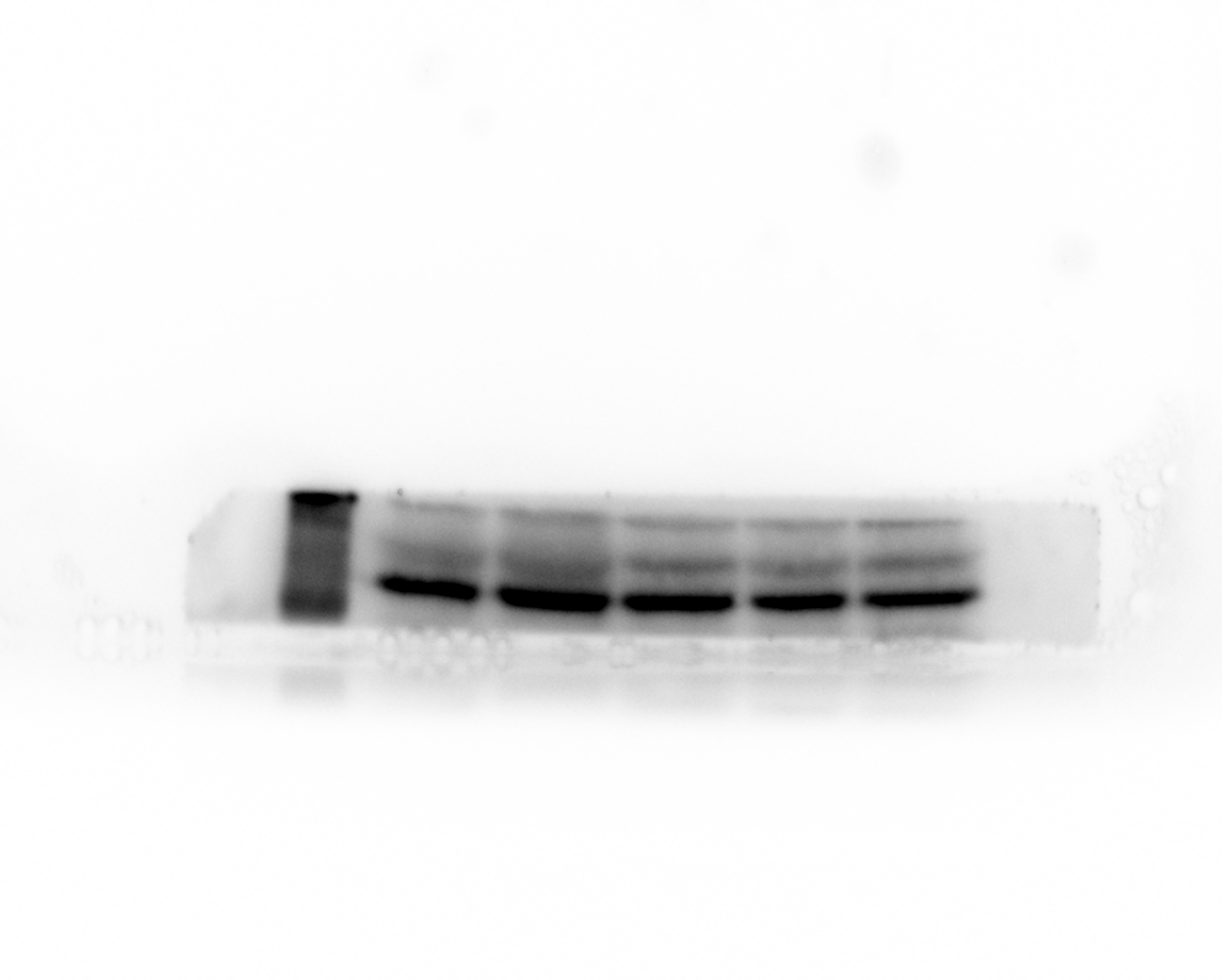

Supplement: Supplementary file 3 [file Data_Sheet_2.ZIP › fig5-wb/p38.tif]

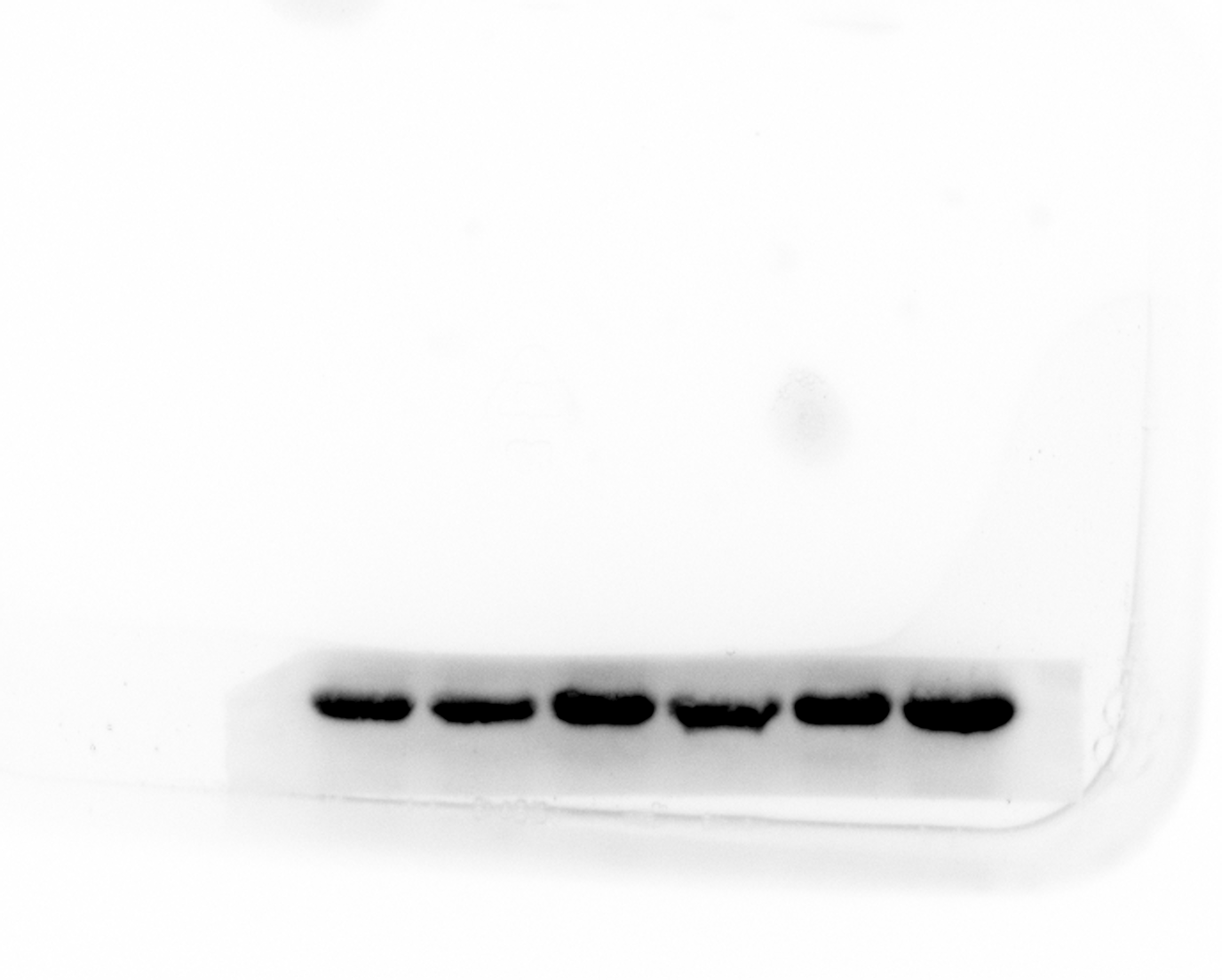

Supplement: Supplementary file 4 [file Data_Sheet_3.ZIP › fig10-wb/actin.tif]

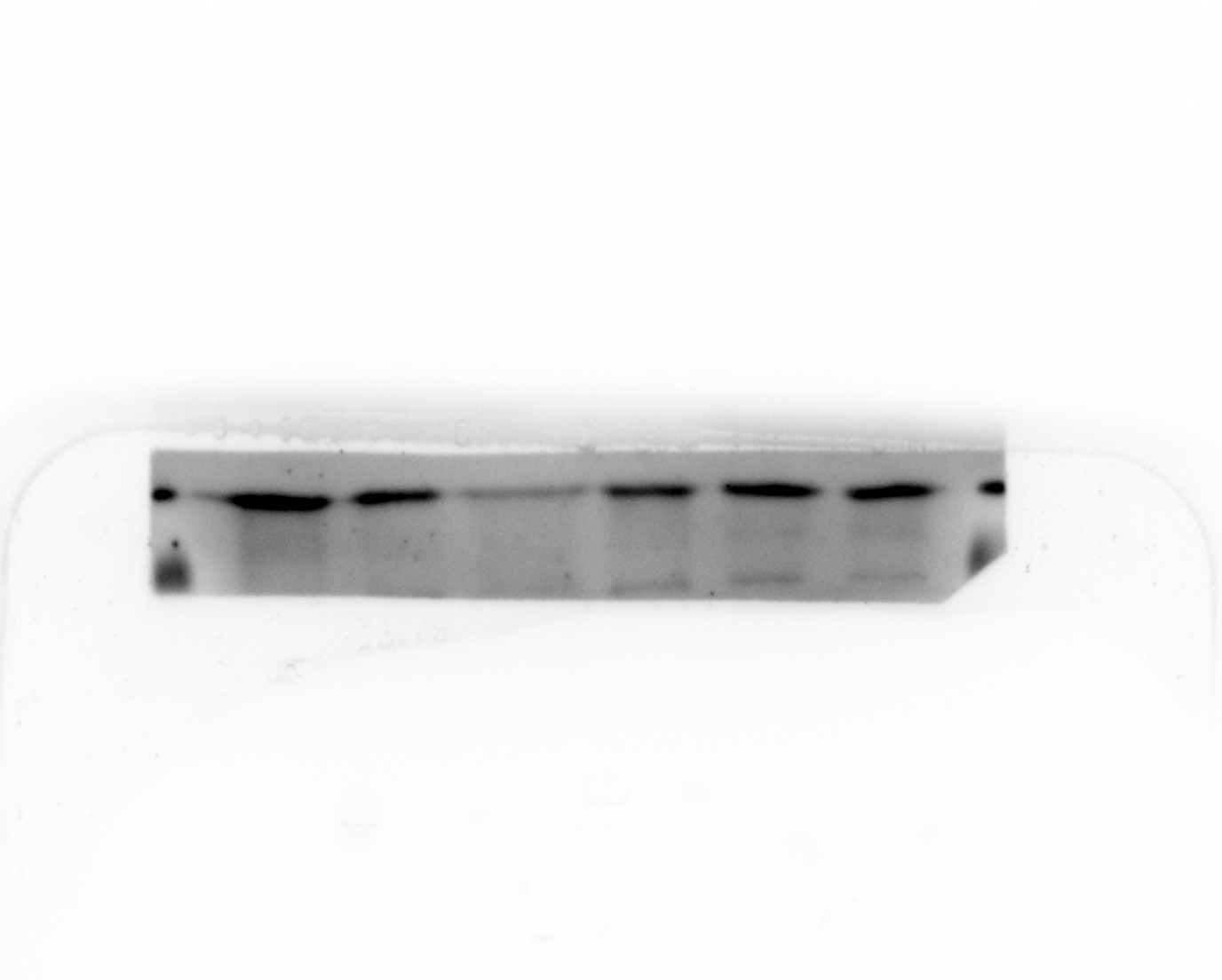

Supplement: Supplementary file 4 [file Data_Sheet_3.ZIP › fig10-wb/cl1.tif]

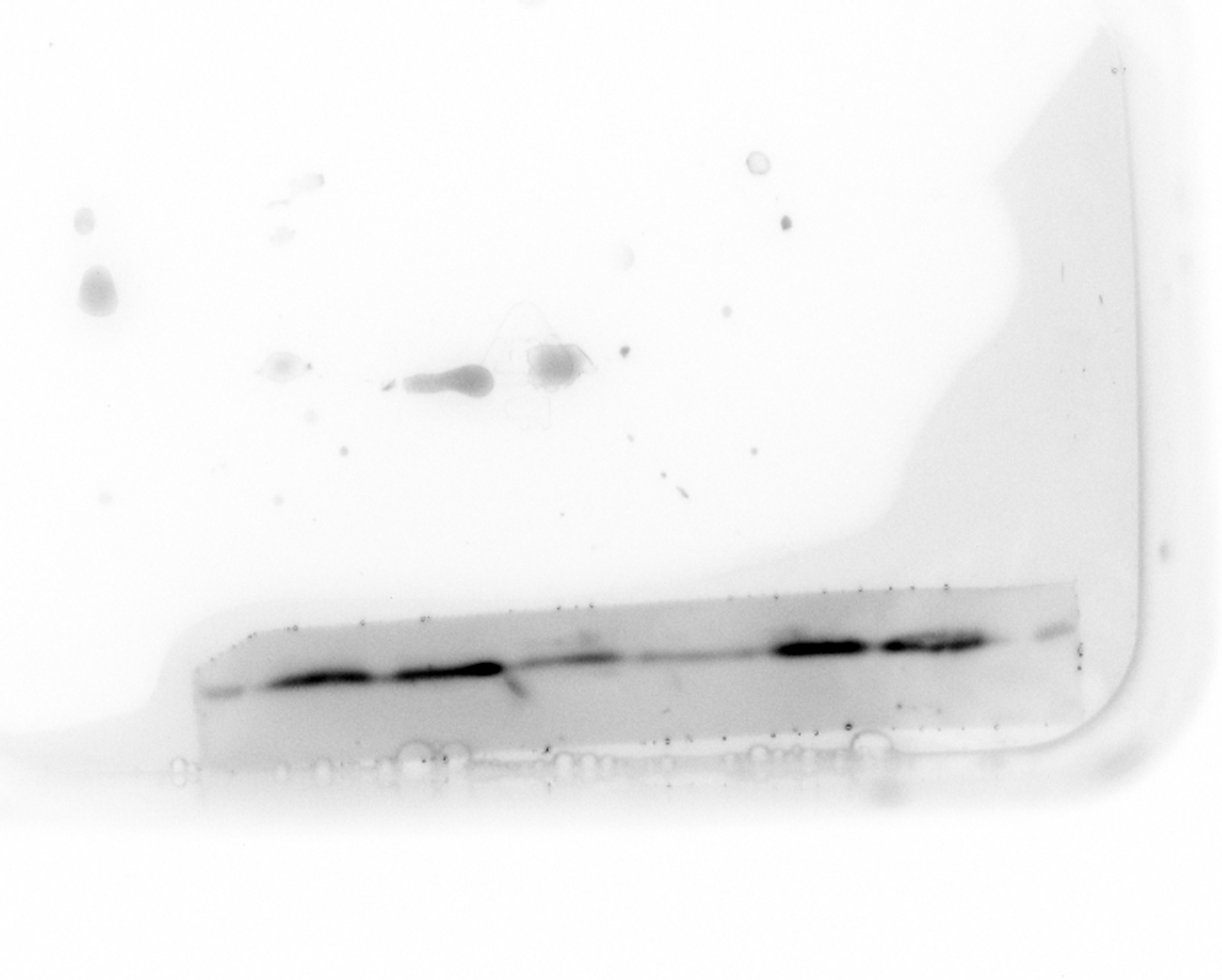

Supplement: Supplementary file 4 [file Data_Sheet_3.ZIP › fig10-wb/occ-╩Ñ═■.tif]

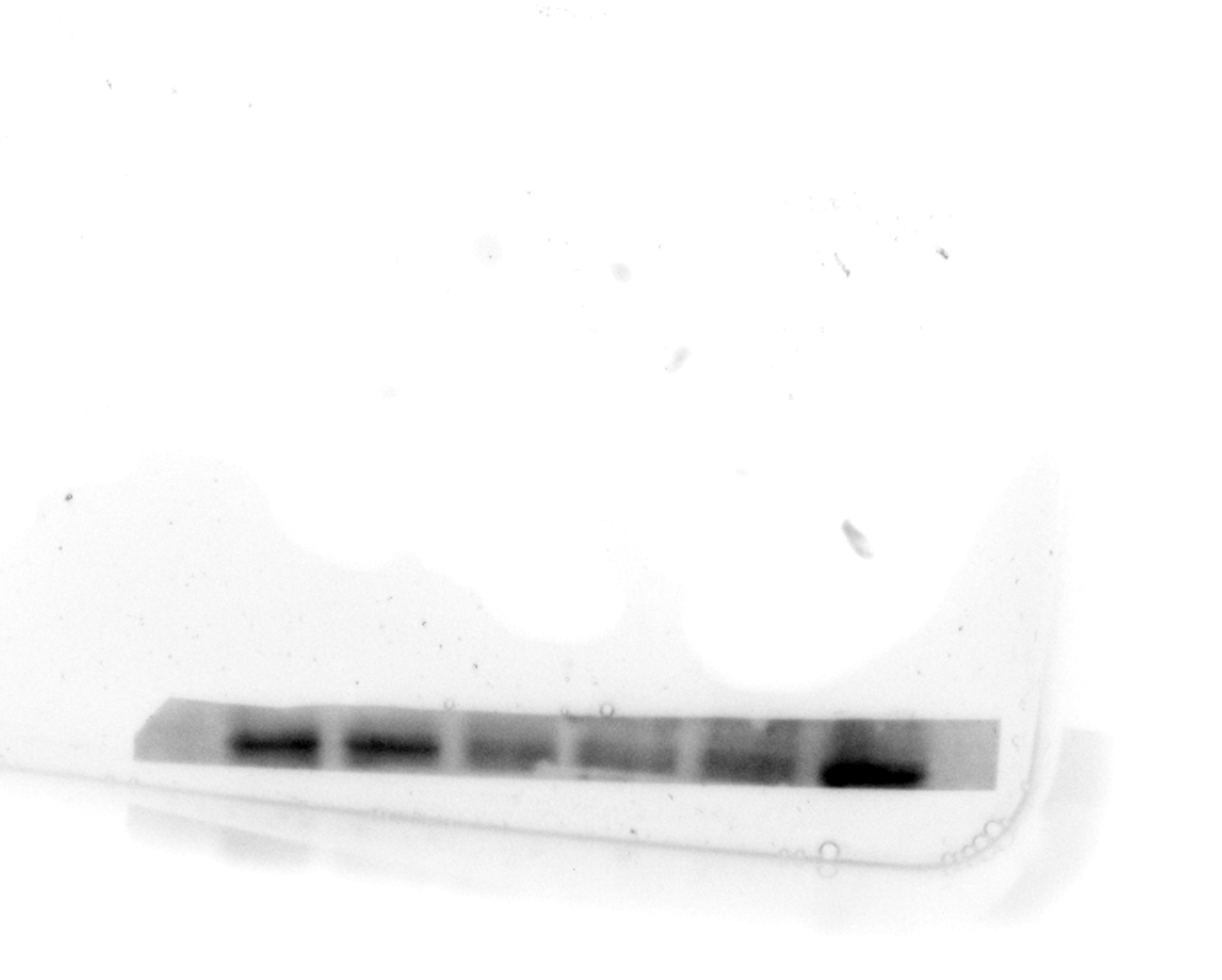

Supplement: Supplementary file 4 [file Data_Sheet_3.ZIP › fig10-wb/zo-1.tif]

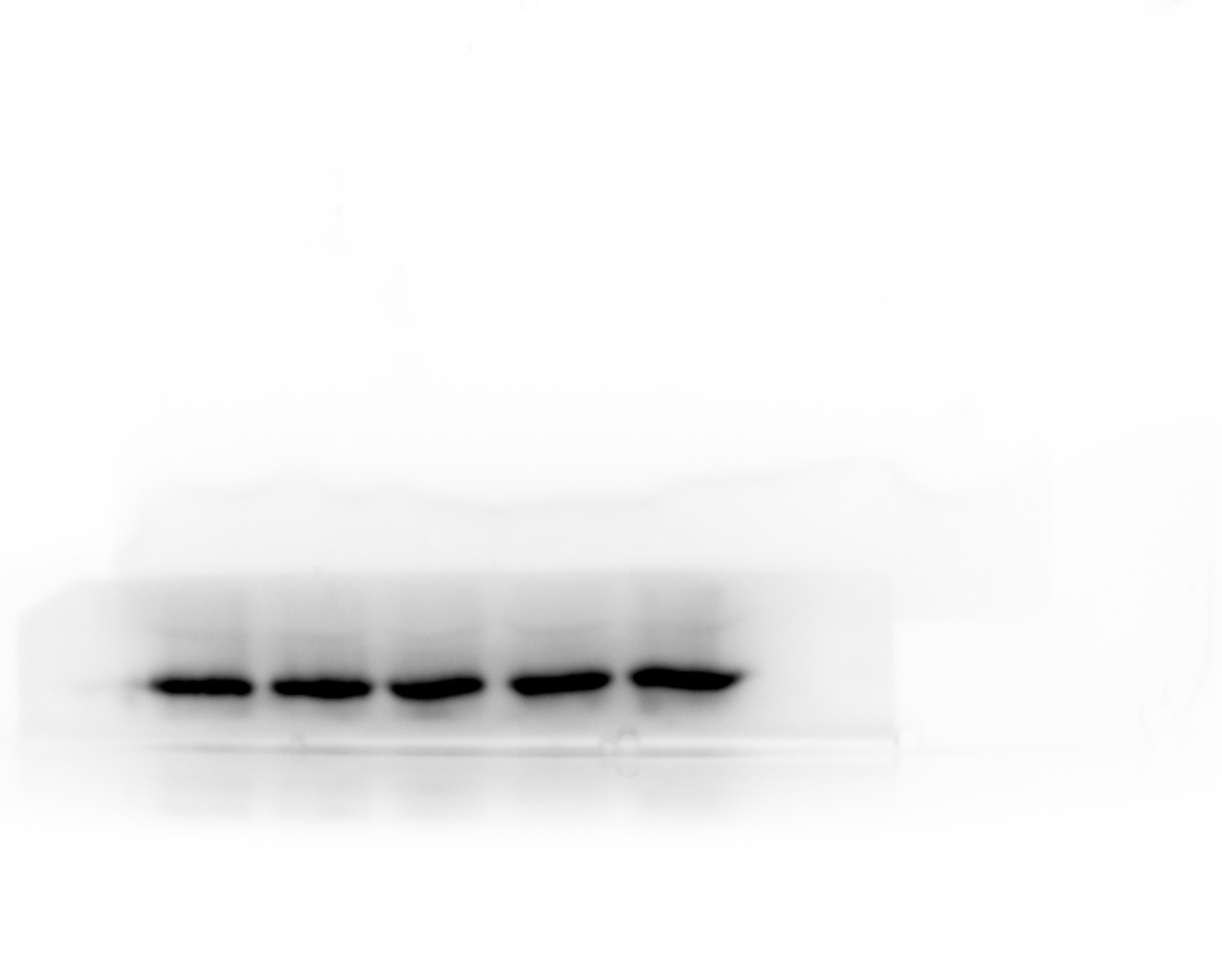

Supplement: Supplementary file 5 [file Data_Sheet_4.ZIP › fig4-wb/IKB.tif]

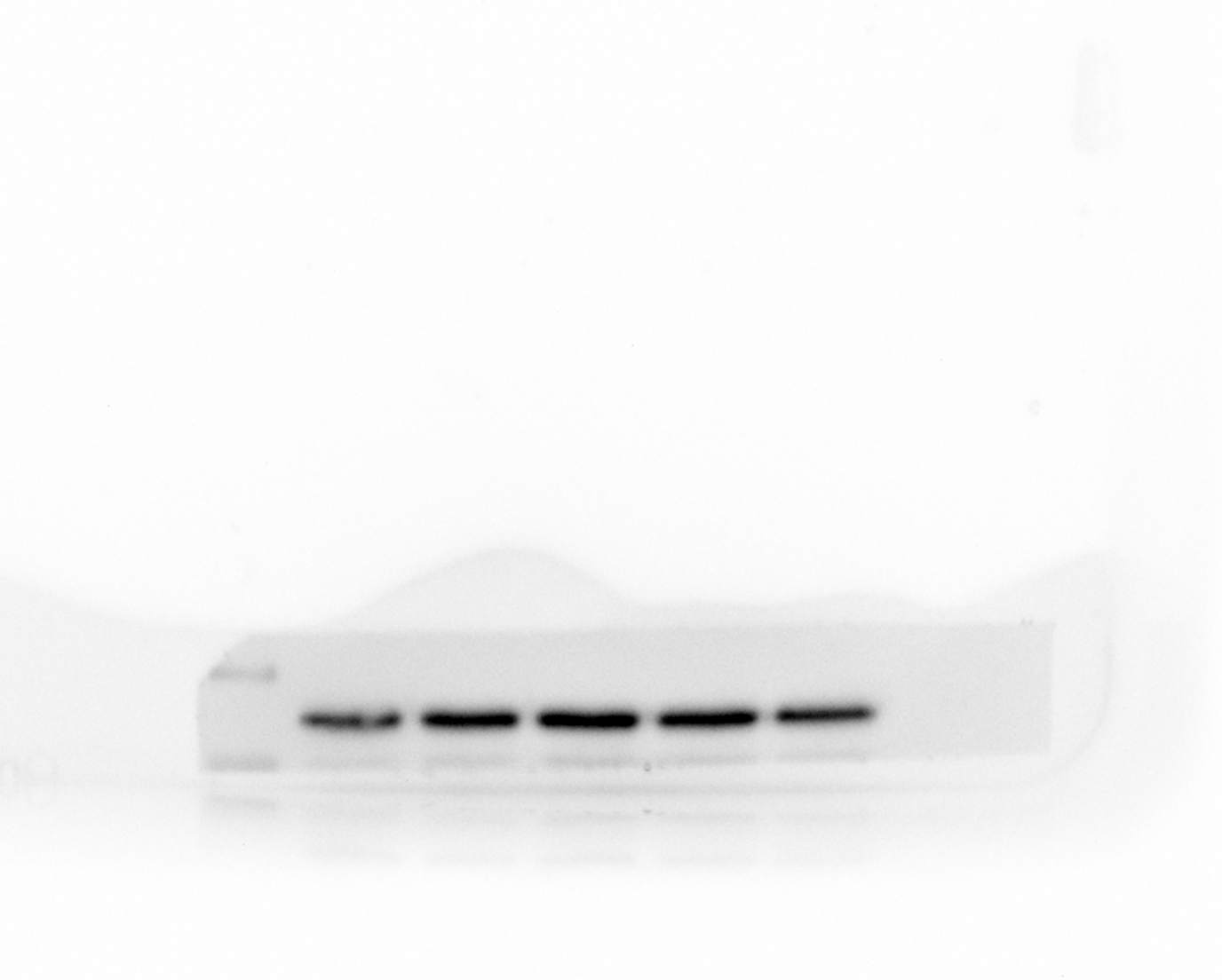

Supplement: Supplementary file 5 [file Data_Sheet_4.ZIP › fig4-wb/N-p-p65-4.tif]

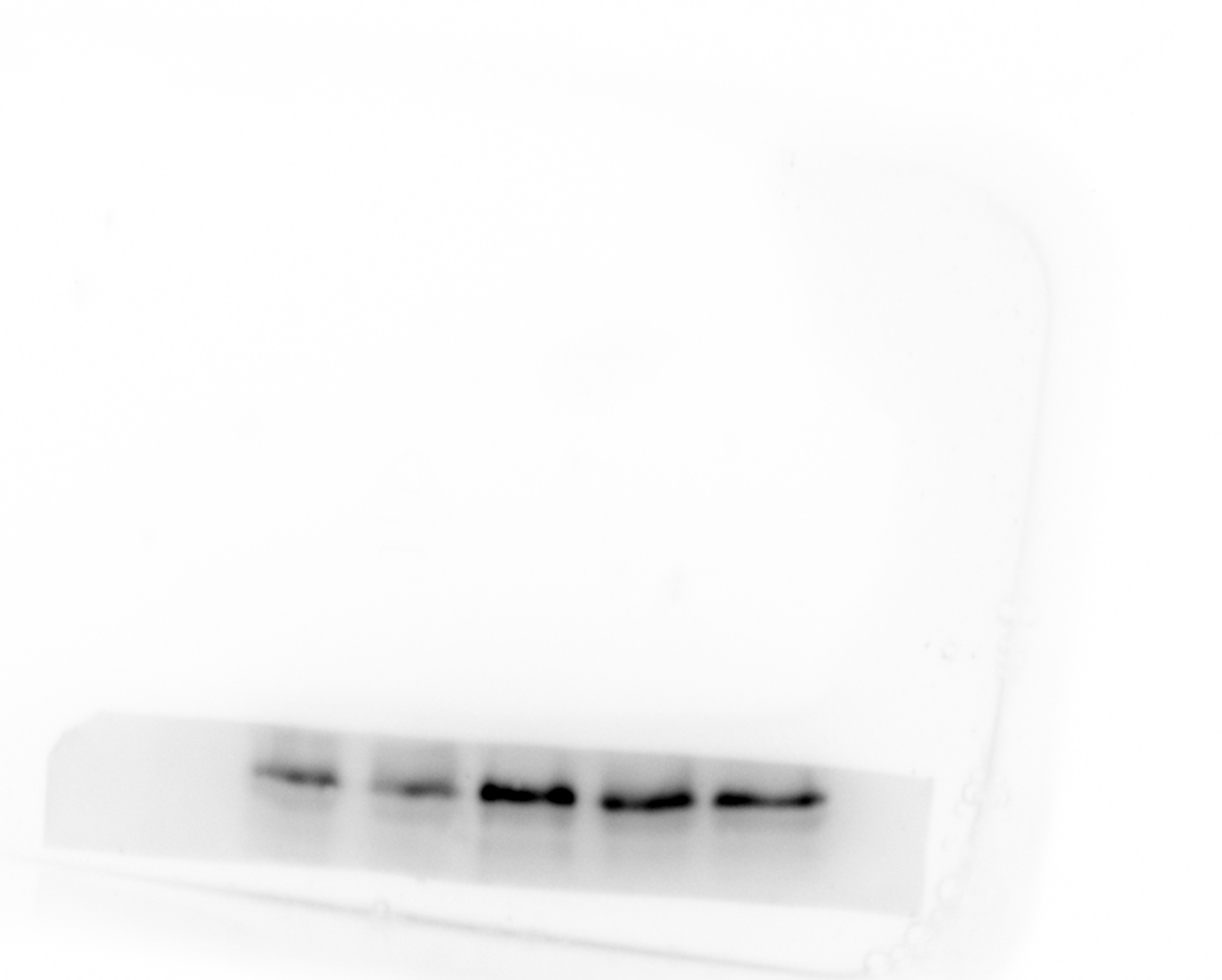

Supplement: Supplementary file 5 [file Data_Sheet_4.ZIP › fig4-wb/P65.tif]

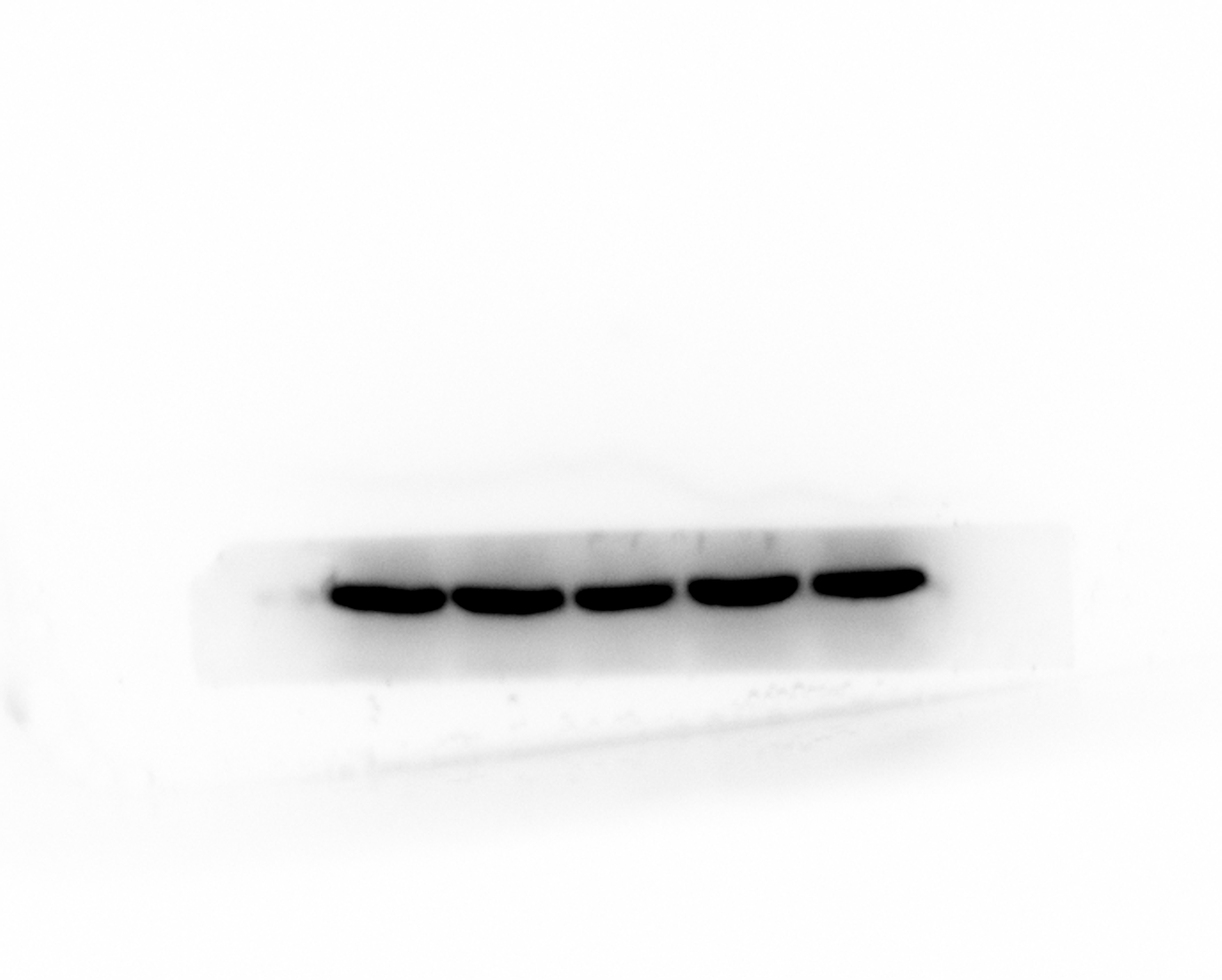

Supplement: Supplementary file 5 [file Data_Sheet_4.ZIP › fig4-wb/actin.tif]

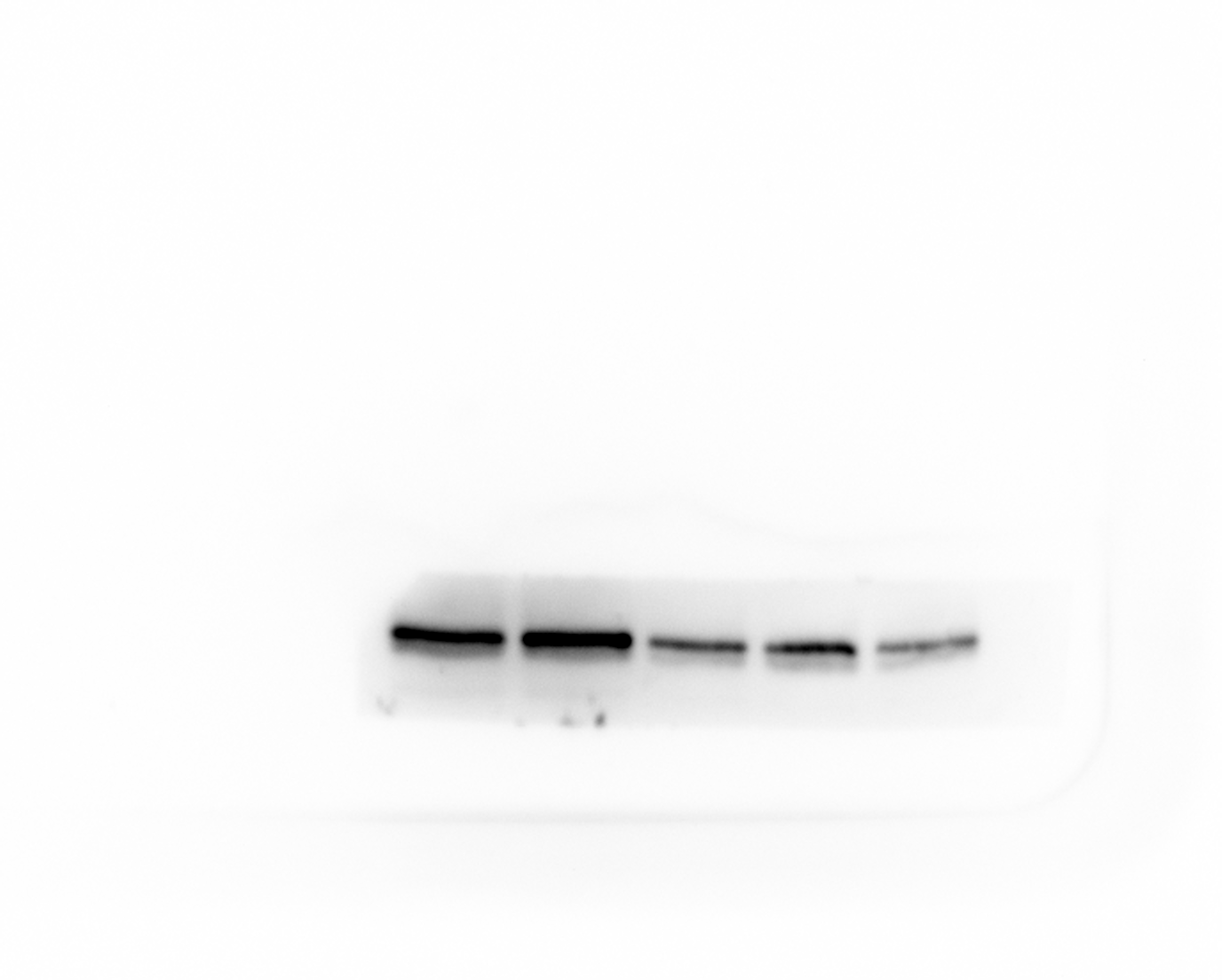

Supplement: Supplementary file 5 [file Data_Sheet_4.ZIP › fig4-wb/n-p-p65-3.tif]

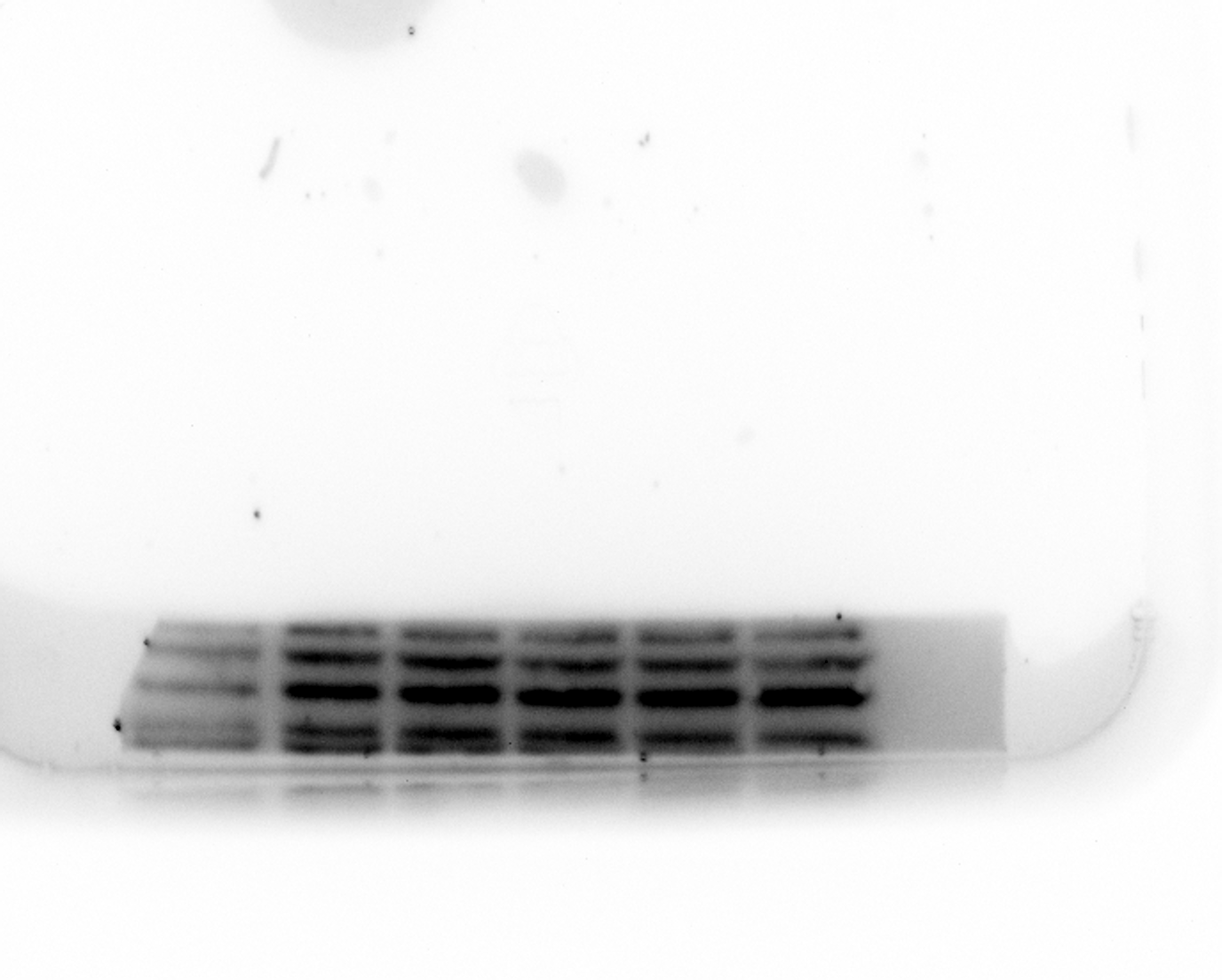

Supplement: Supplementary file 5 [file Data_Sheet_4.ZIP › fig4-wb/p-ikb.tif]

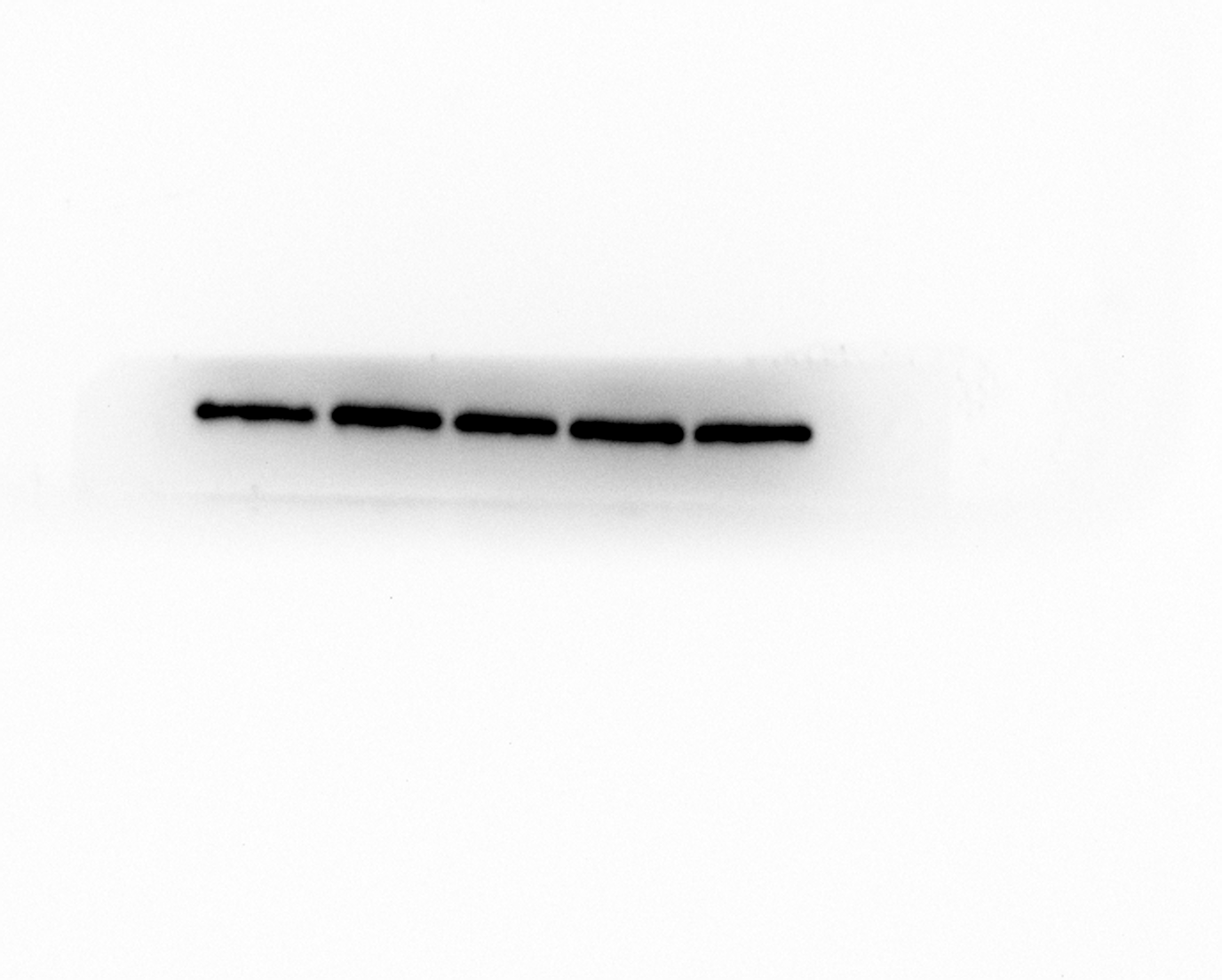

Supplement: Supplementary file 5 [file Data_Sheet_4.ZIP › fig4-wb/p-p65-2.tif]

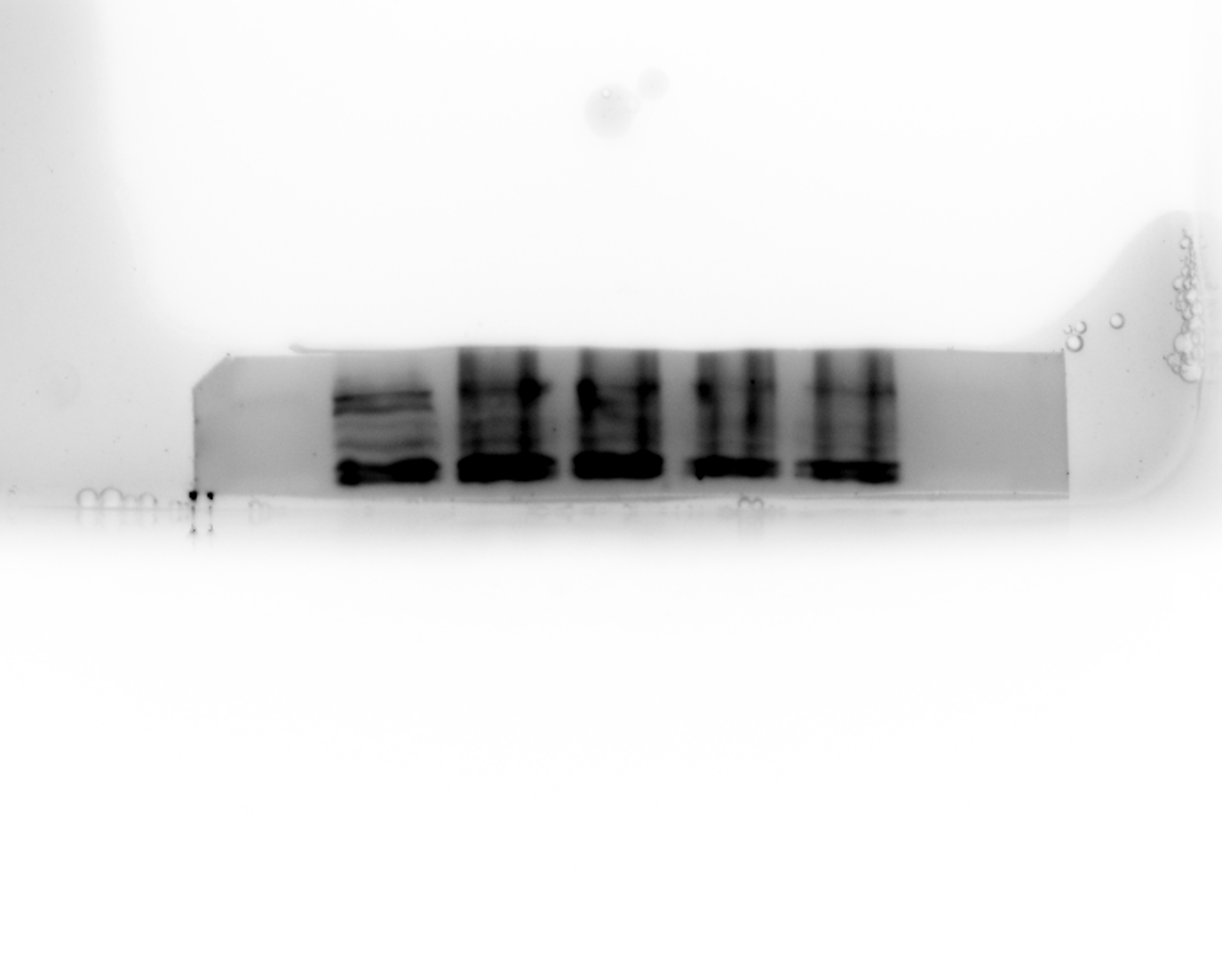

Supplement: Supplementary file 5 [file Data_Sheet_4.ZIP › fig4-wb/tlr4-3.tif]

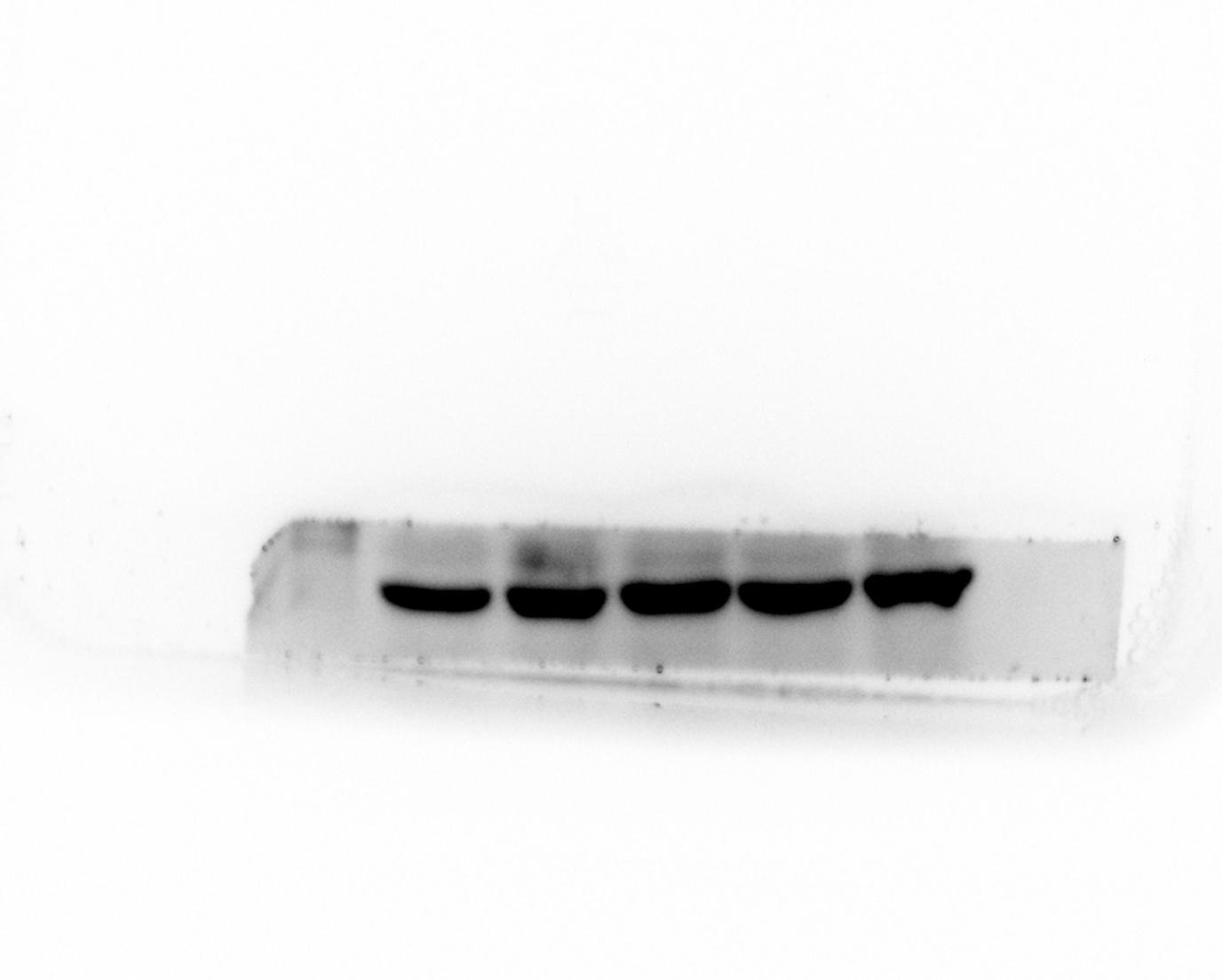

Supplement: Supplementary file 6 [file Data_Sheet_5.ZIP › fig6-wb/actin.tif]

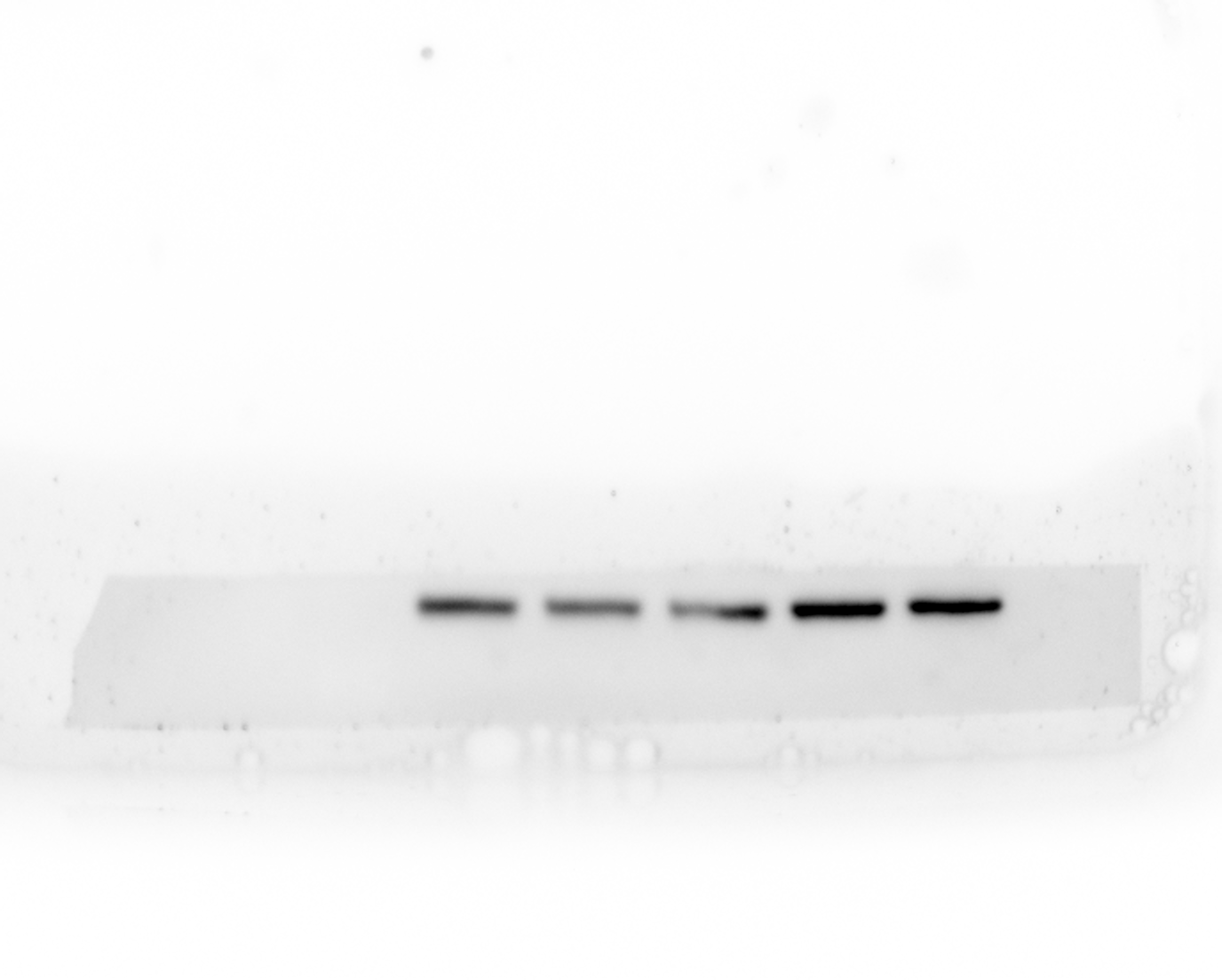

Supplement: Supplementary file 6 [file Data_Sheet_5.ZIP › fig6-wb/c1-23.tif]

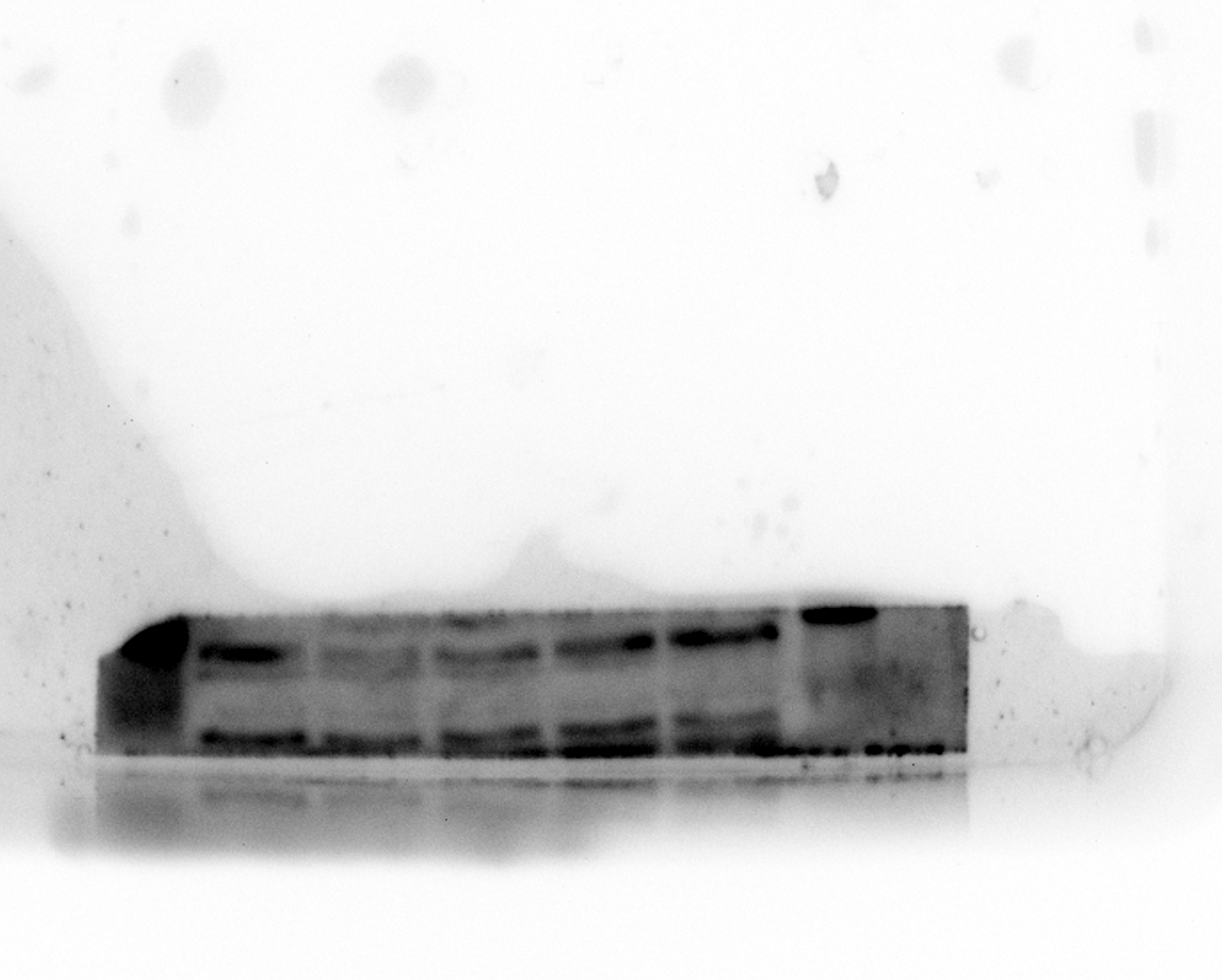

Supplement: Supplementary file 6 [file Data_Sheet_5.ZIP › fig6-wb/occ.tif]

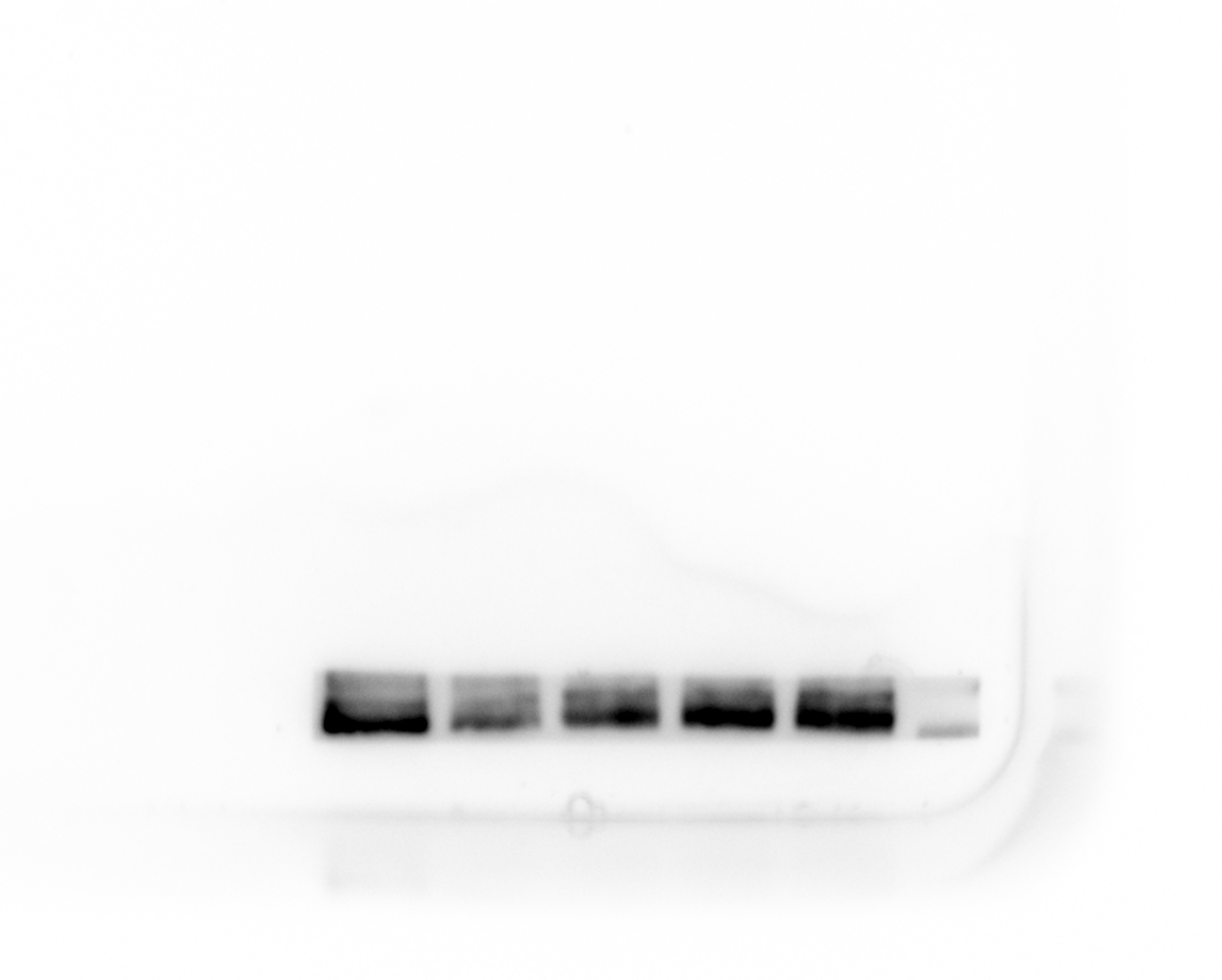

Supplement: Supplementary file 6 [file Data_Sheet_5.ZIP › fig6-wb/zo-1-4.tif]

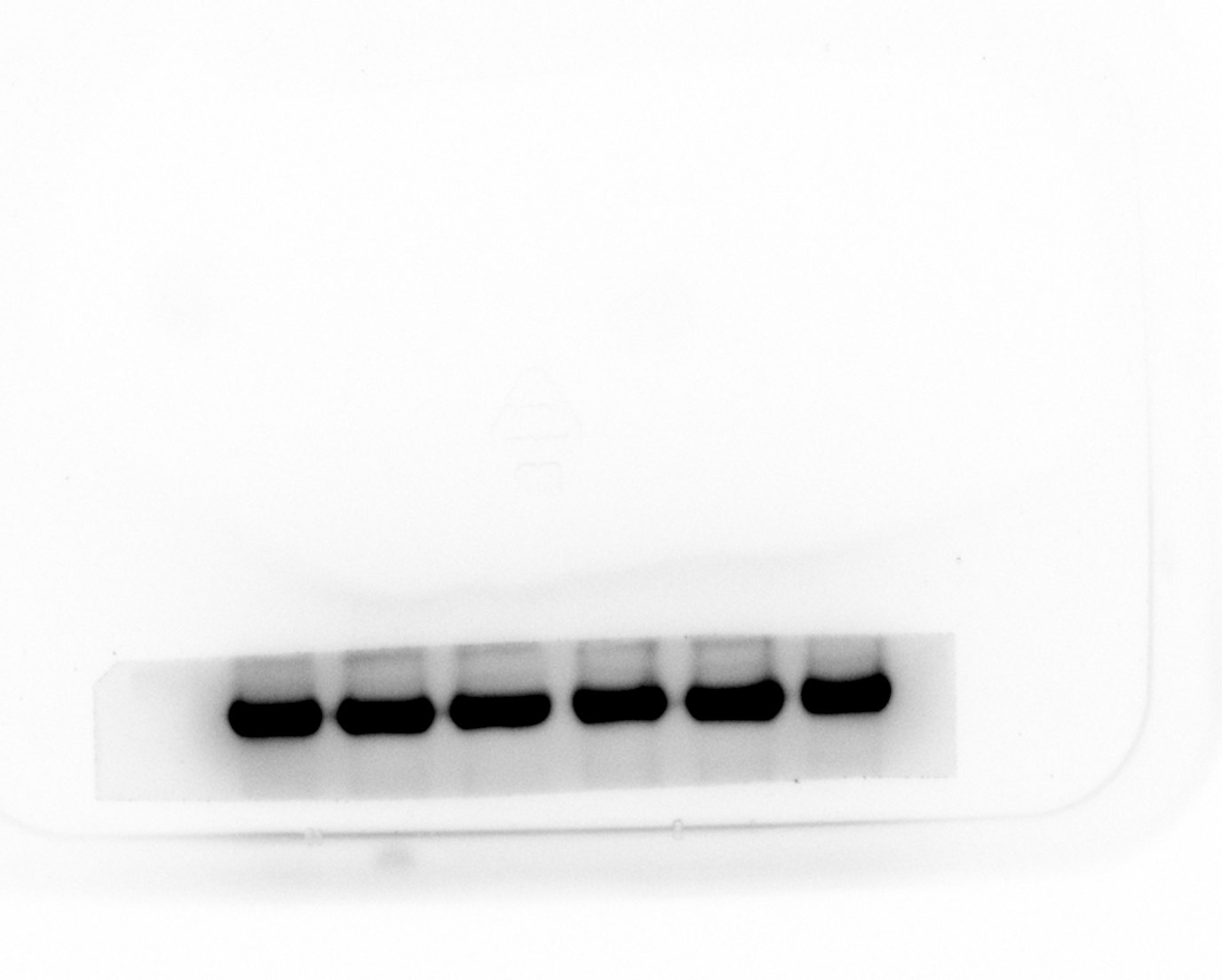

Supplement: Supplementary file 7 [file Data_Sheet_6.ZIP › fig9-wb/actin-.tif]

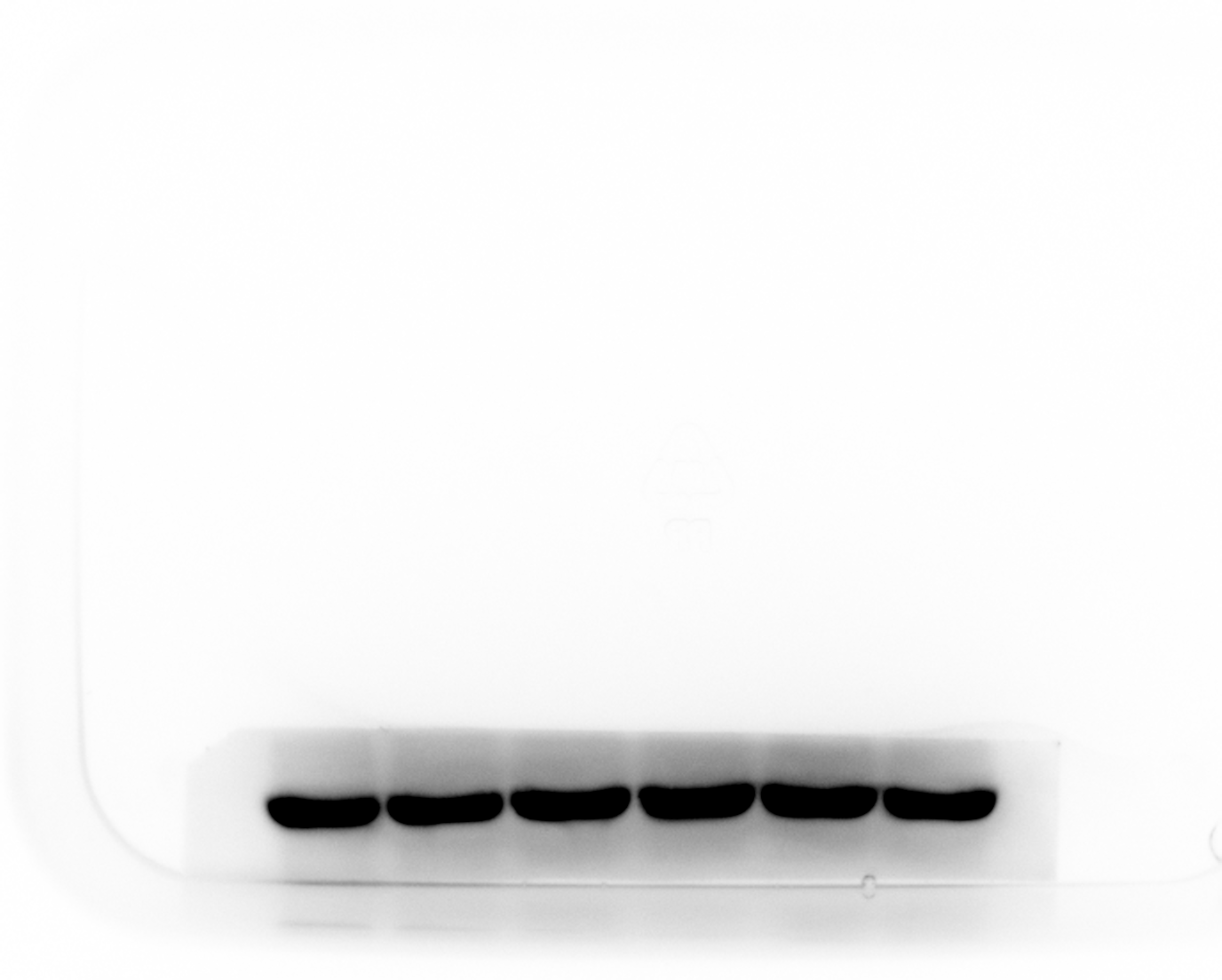

Supplement: Supplementary file 7 [file Data_Sheet_6.ZIP › fig9-wb/actin2.tif]

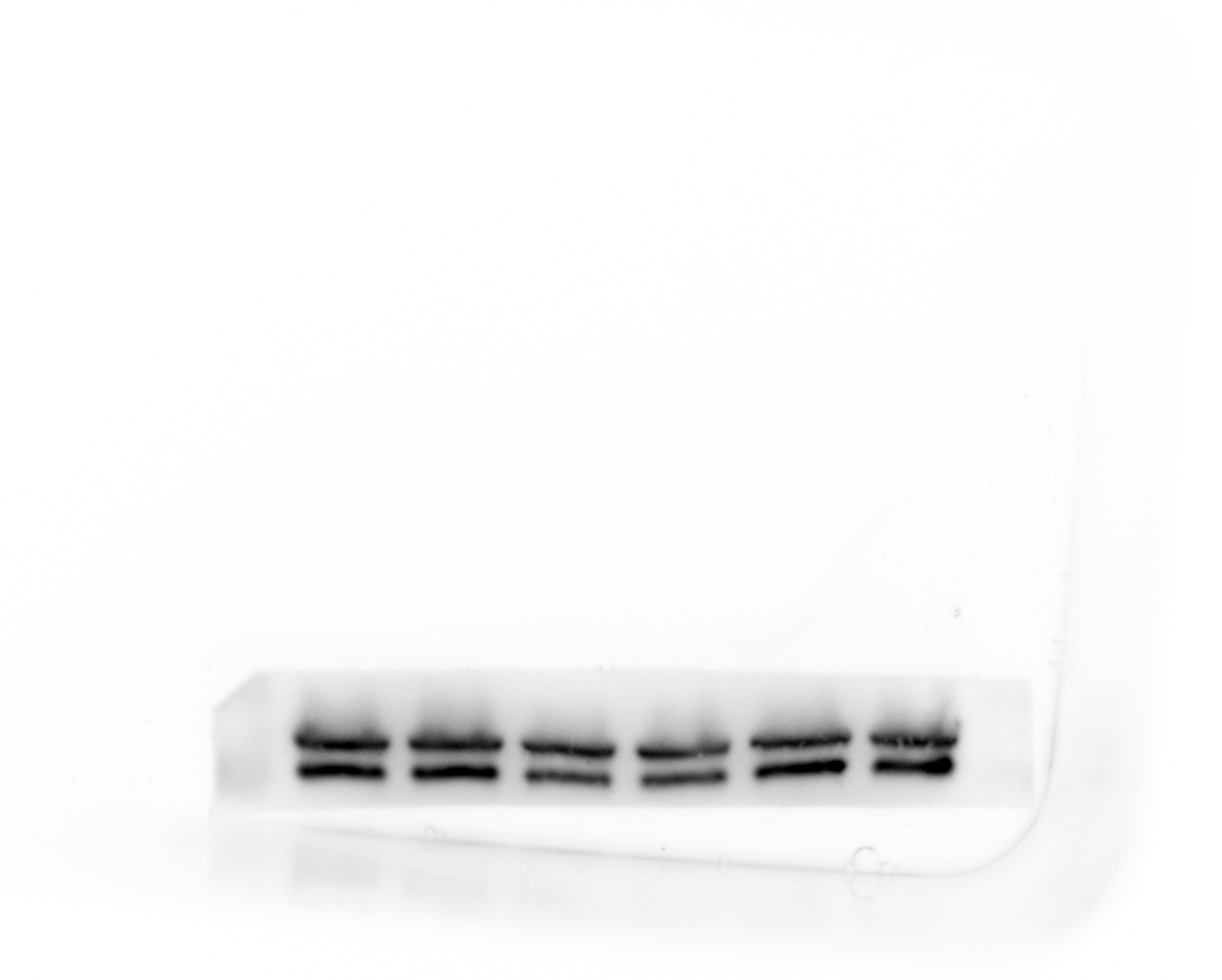

Supplement: Supplementary file 7 [file Data_Sheet_6.ZIP › fig9-wb/erk.tif]

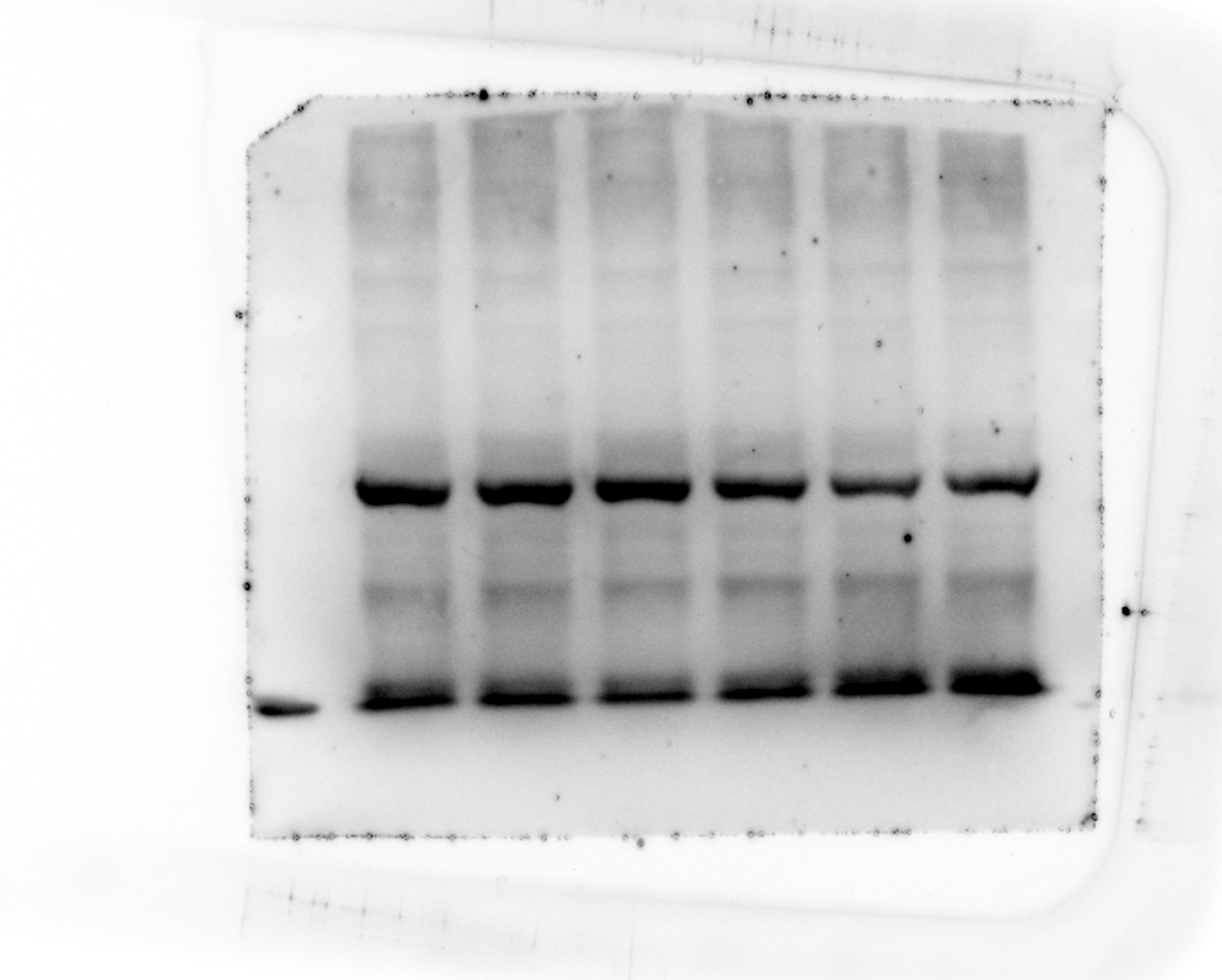

Supplement: Supplementary file 7 [file Data_Sheet_6.ZIP › fig9-wb/ikb.tif]

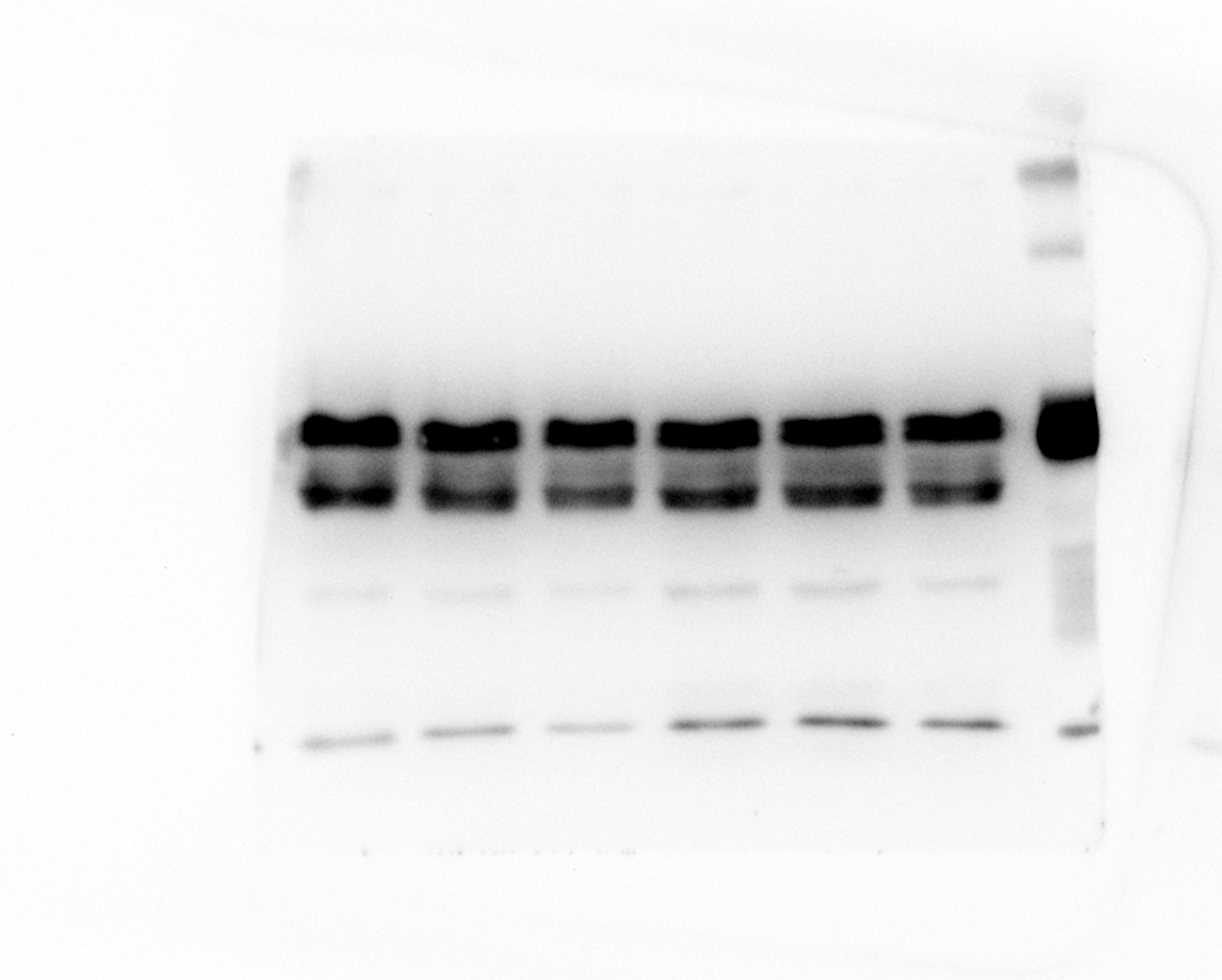

Supplement: Supplementary file 7 [file Data_Sheet_6.ZIP › fig9-wb/jnk------.tif]

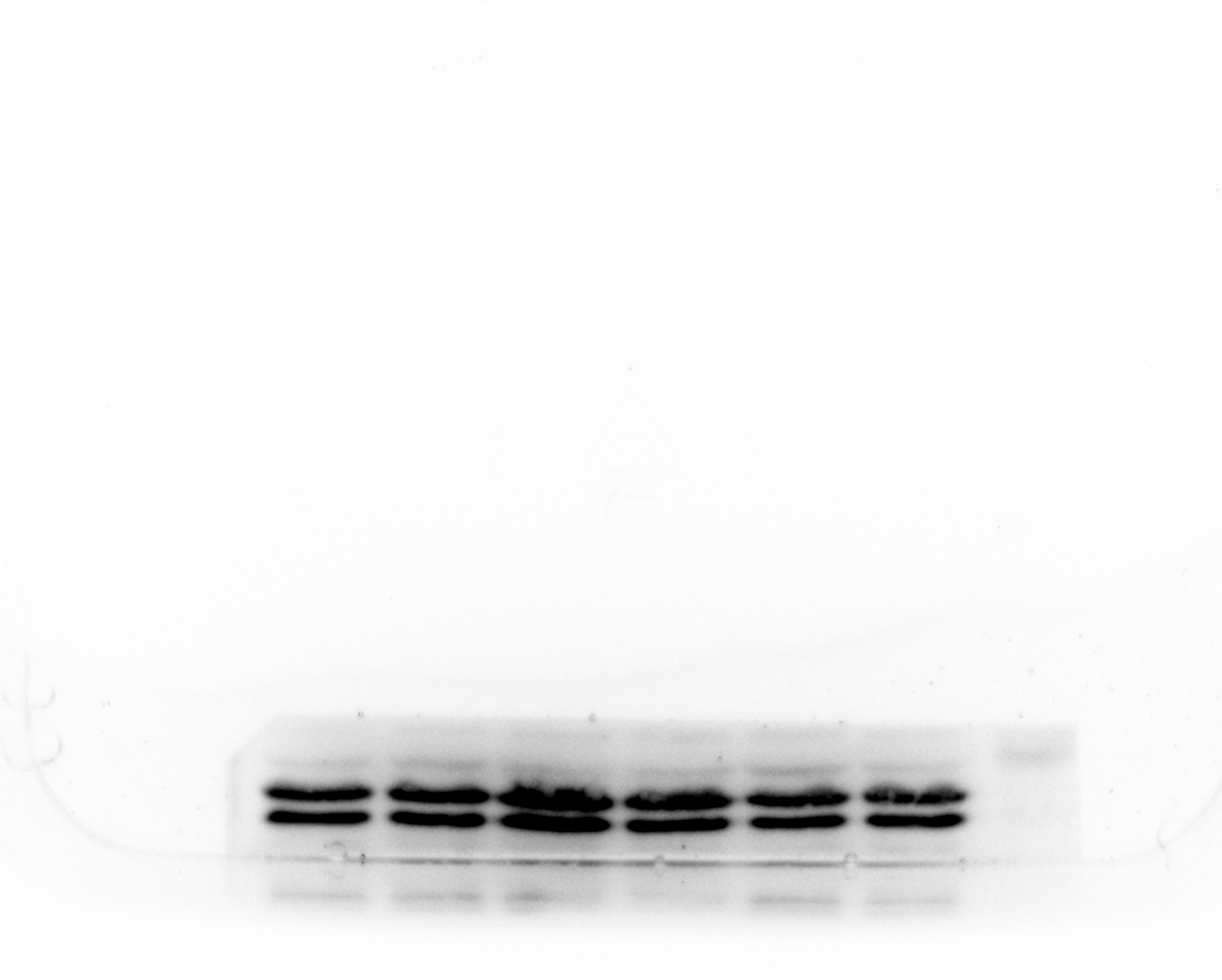

Supplement: Supplementary file 7 [file Data_Sheet_6.ZIP › fig9-wb/p-erk-2.tif]

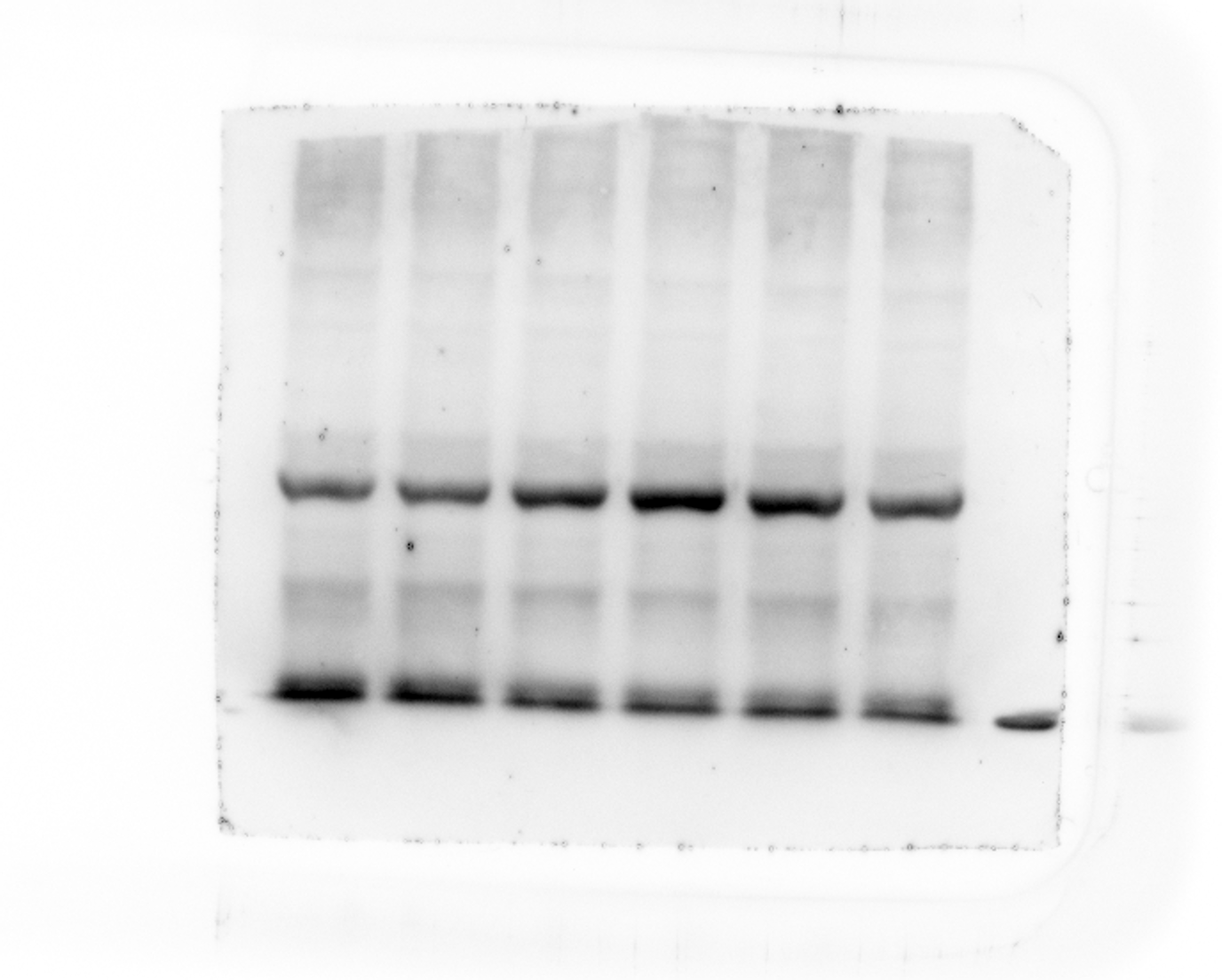

Supplement: Supplementary file 7 [file Data_Sheet_6.ZIP › fig9-wb/p-ikb-2.tif]

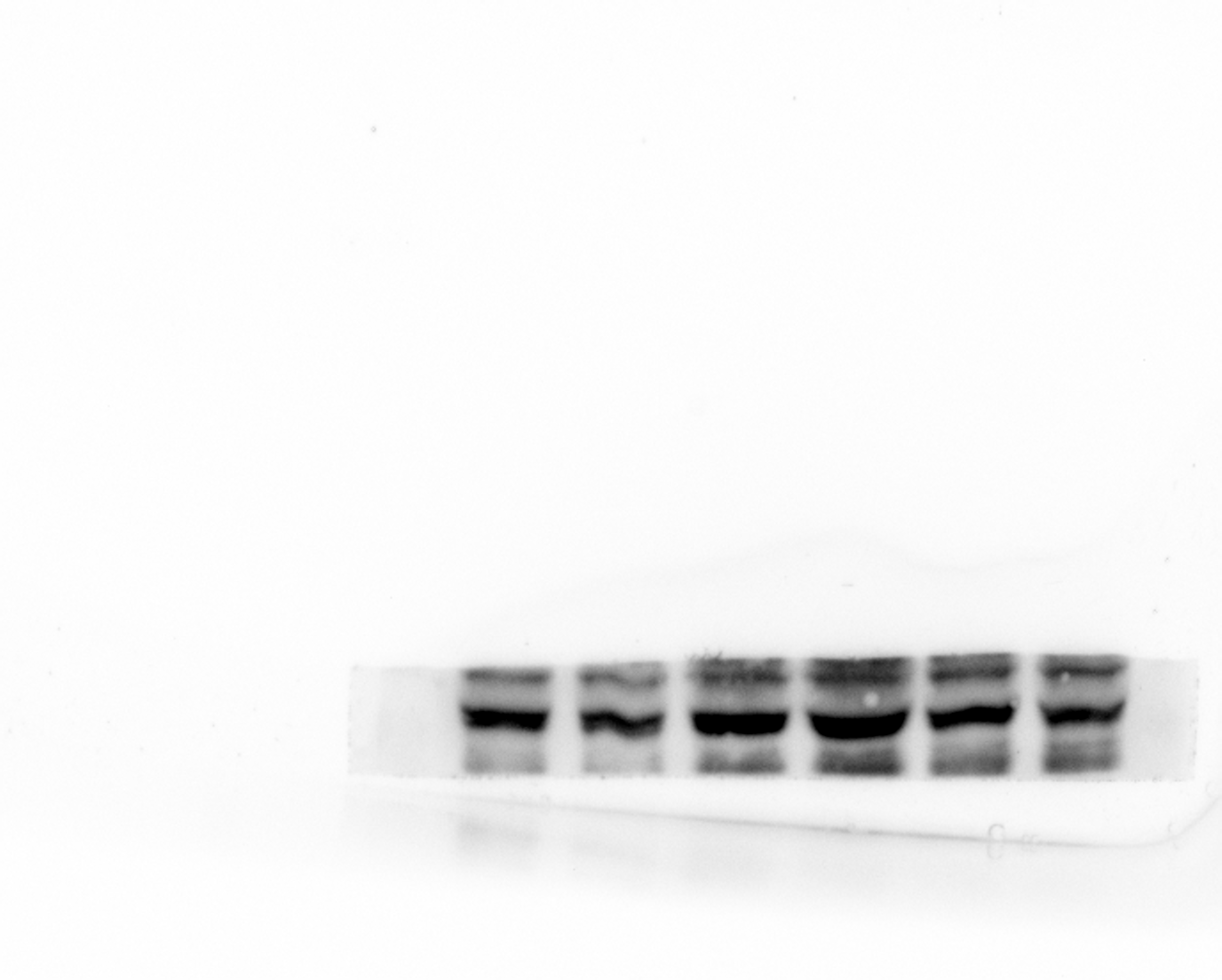

Supplement: Supplementary file 7 [file Data_Sheet_6.ZIP › fig9-wb/p-jnk-3.tif]

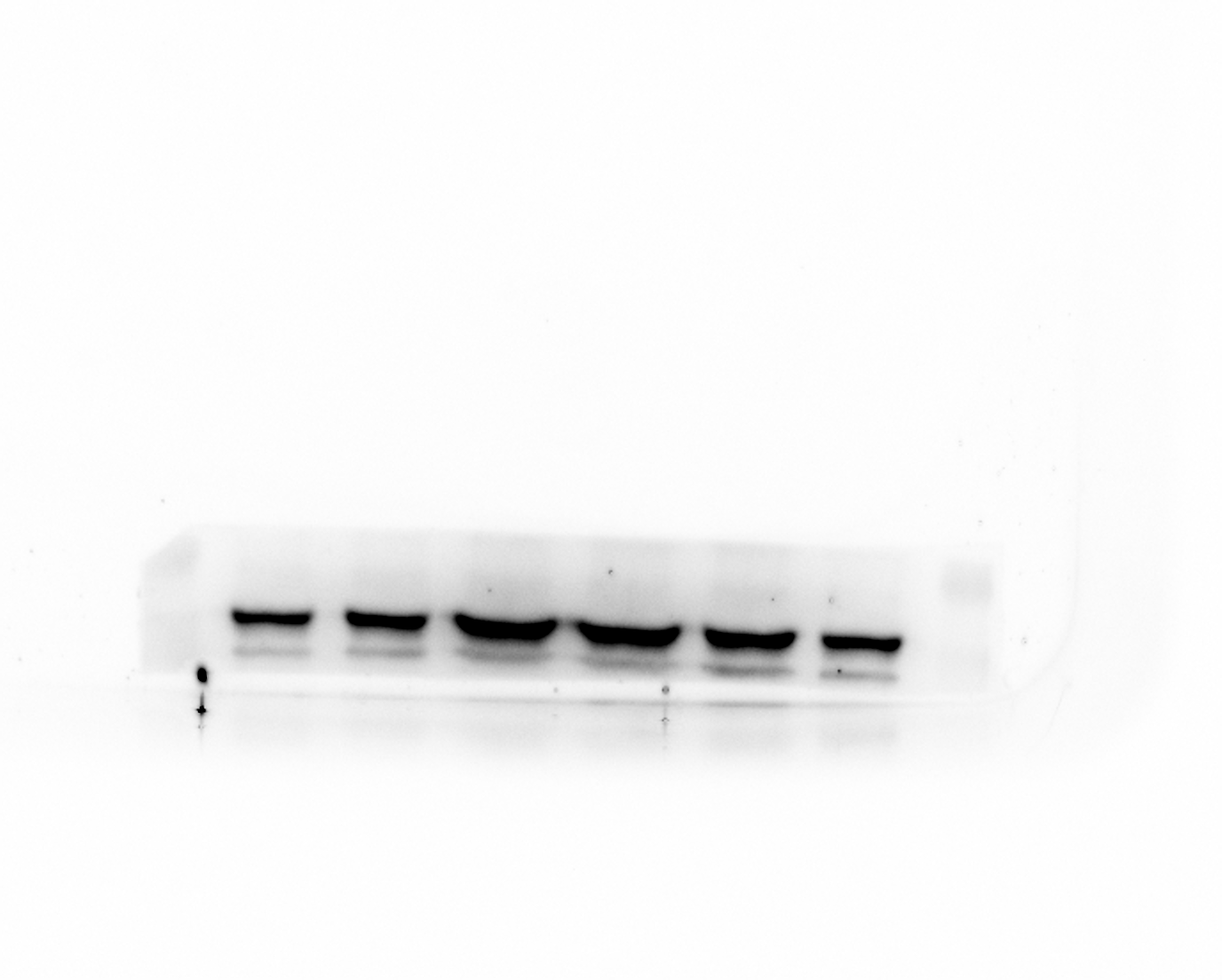

Supplement: Supplementary file 7 [file Data_Sheet_6.ZIP › fig9-wb/p-p38-2.tif]

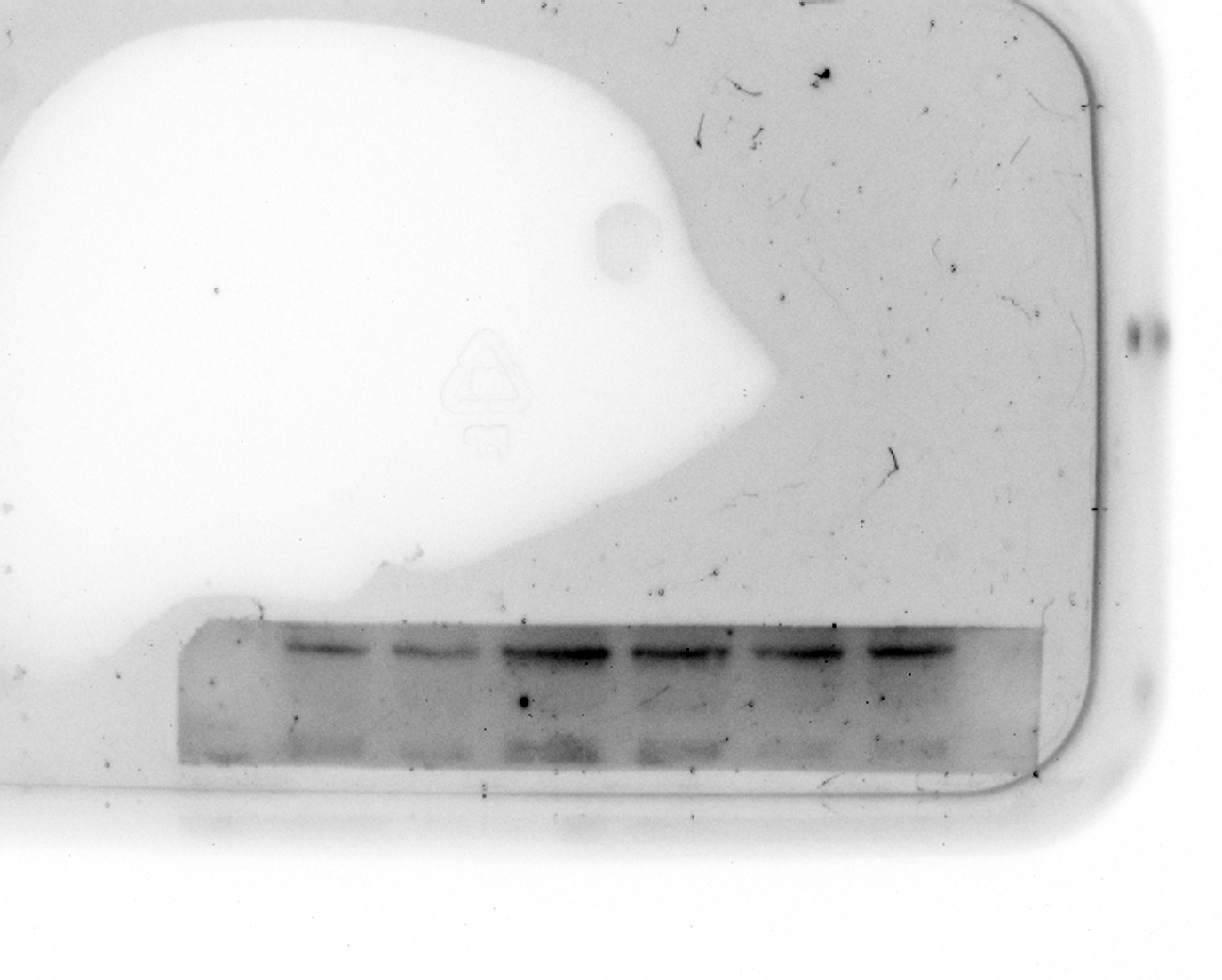

Supplement: Supplementary file 7 [file Data_Sheet_6.ZIP › fig9-wb/p-p65-4.tif]

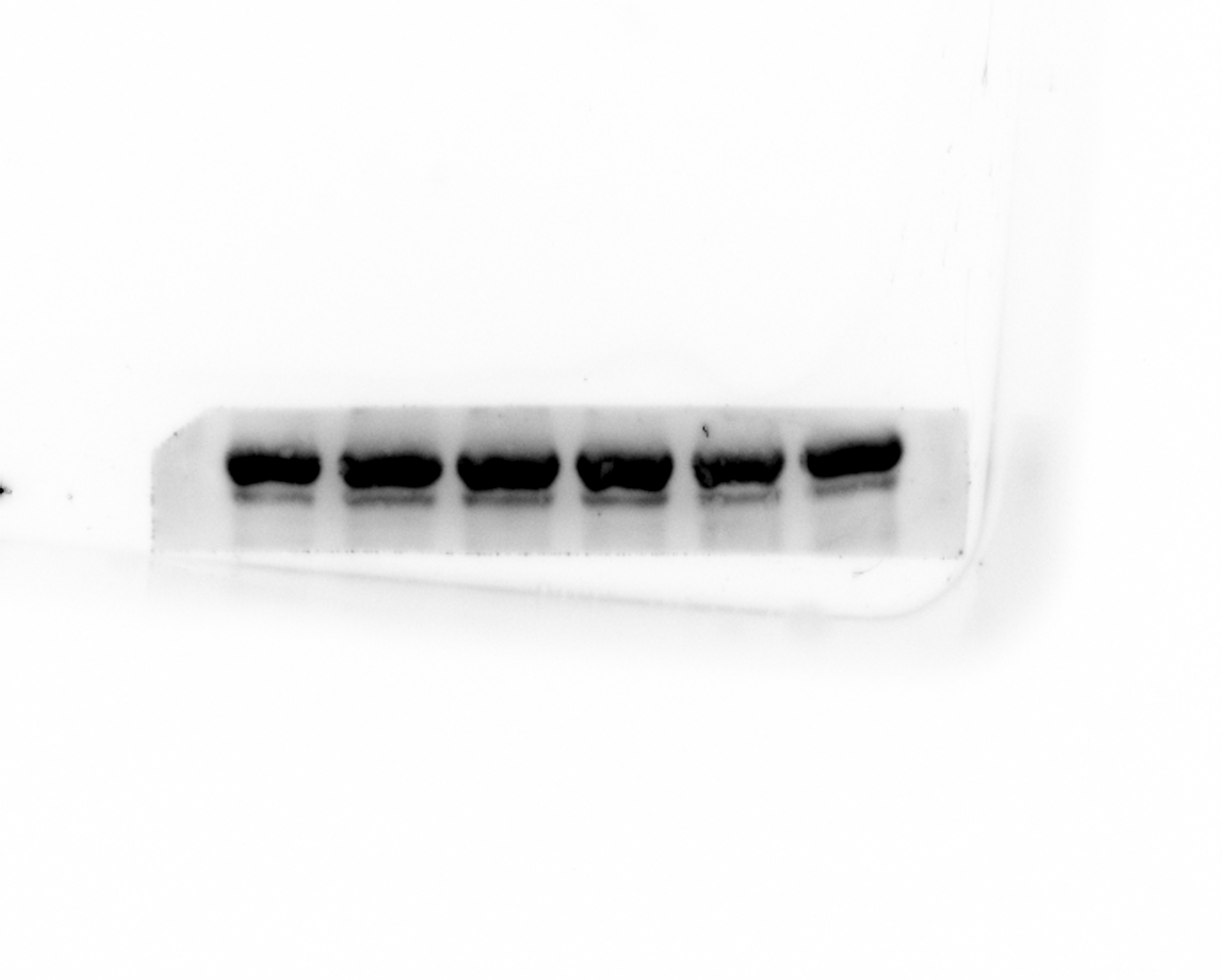

Supplement: Supplementary file 7 [file Data_Sheet_6.ZIP › fig9-wb/p38.tif]

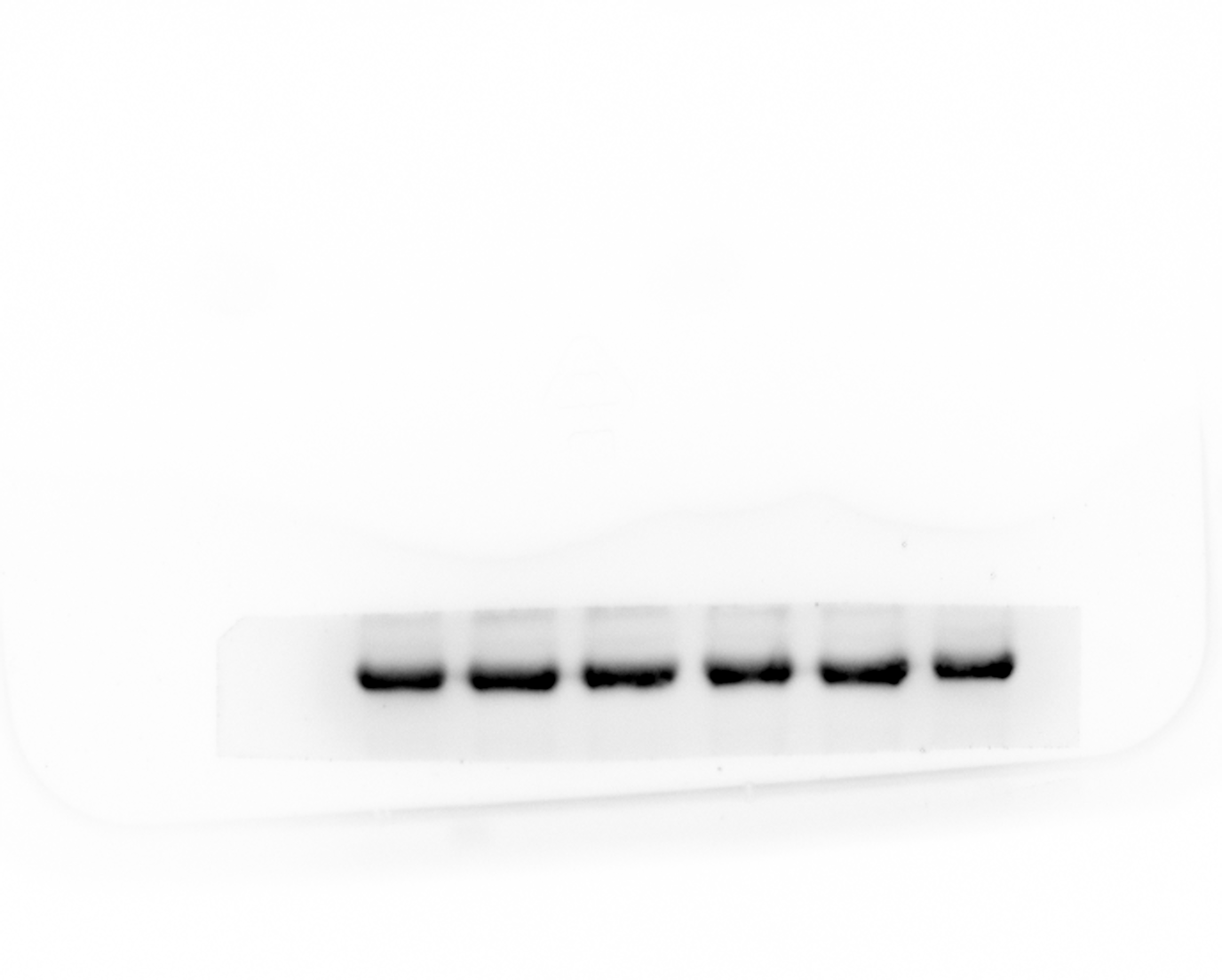

Supplement: Supplementary file 7 [file Data_Sheet_6.ZIP › fig9-wb/p65-2.tif]

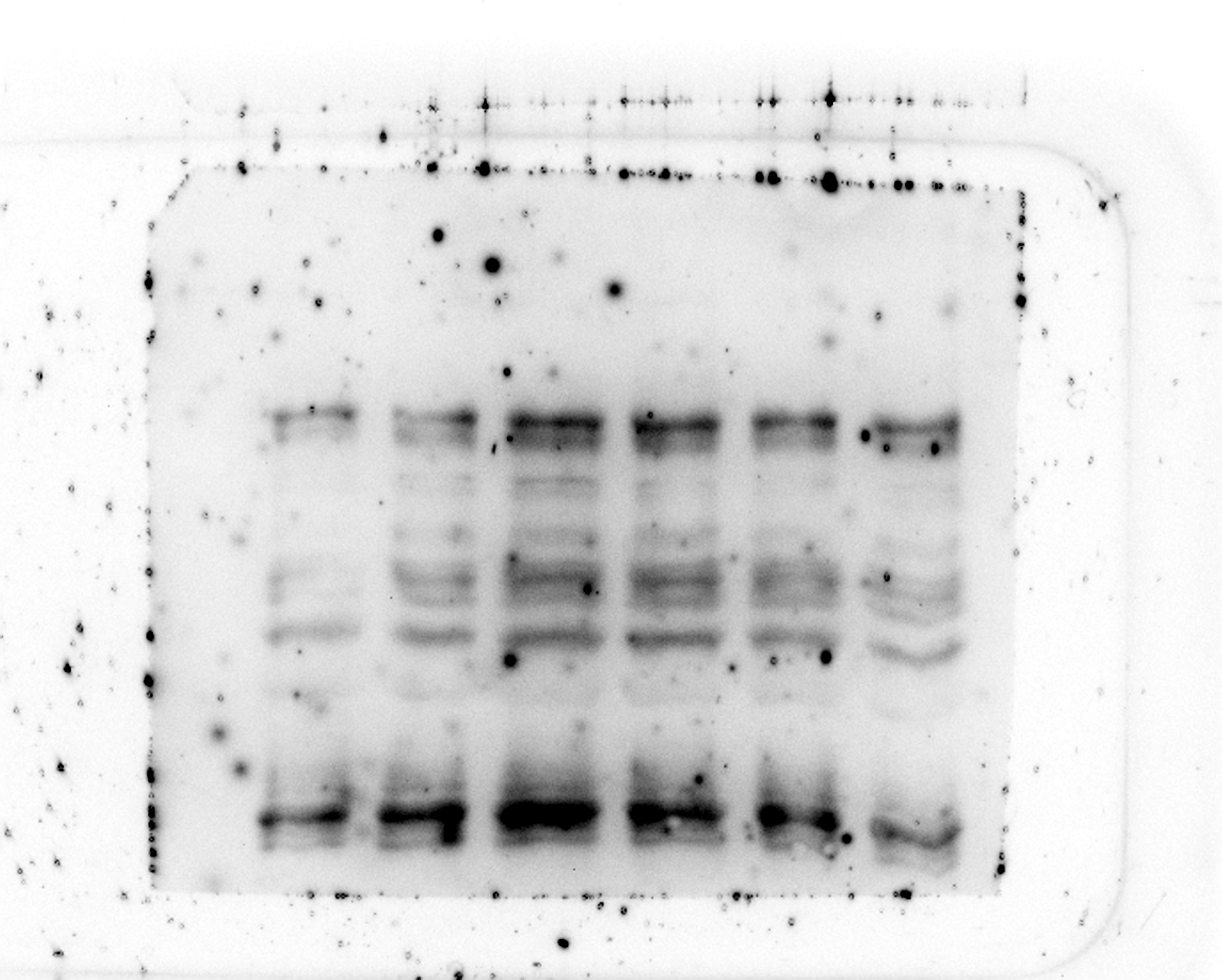

Supplement: Supplementary file 7 [file Data_Sheet_6.ZIP › fig9-wb/tlr4.tif]
